# High-Throughput Screening and Confirmation of 420 Hazardous Substances in Feed Based on Liquid Chromatography−High-Resolution Mass Spectrometry

**DOI:** 10.3390/foods15030502

**Published:** 2026-02-01

**Authors:** Jie Wang, Xu Gu, Ming Jia, Yunfeng Gao, Peng Wang, Wenlong Du, Qingshi Meng, Jing Li, Donghui Liu

**Affiliations:** 1 Department of Applied Chemistry, China Agricultural University, No. 2 Yuanmingyuan West Road, Beijing 100193, China; 2 Feed Research Institute, Chinese Academy of Agricultural Sciences, Beijing 100081, China; 3 Heilongjiang Province Agricultural Products and Veterinary Medicine Feed Technical Identification Station, Harbin 150090, China; 4 Institute of Animal Science, Chinese Academy of Agricultural Sciences, Beijing 100193, China

**Keywords:** screening, confirmation, hazardous substance, high-resolution mass spectrometry, feed

## Abstract

Detection of hazardous substances in feed is important for ensuring human health. A method based on liquid chromatography-high-resolution mass spectrometry (LC-HRMS) was developed and validated for the screening and confirmation of 420 hazardous substances, including pesticides, veterinary drugs, and mycotoxins commonly found in feed. The screening phase employed less stringent criteria to minimize false negatives caused by matrix effects. Subsequently, stricter identification criteria were applied for confirmation to avoid false positives from interfering compounds. The performance of the proposed method was verified by limit of detection (LOD, 5~500 μg/L), screening detection limits (SDL, 50~500 μg/L), matrix effect (ME, 36.12~121.16%), precision (0.02~14.98%), stability, and accuracy. The method was successfully applied to real feed samples, demonstrating its capability to detect the presence of the 420 target hazardous substances. We believe our method provides strong technical support for ensuring the quality and safety of feed.

## 1. Introduction

The continuous standardisation of the breeding industry and industrialisation of livestock husbandry have brought animal-origin foods to the forefront of food demand. However, animal products typically have long breeding cycles. To ensure disease-free production and satisfactory quality of animal-origin foods, veterinary drugs are widely used throughout different stages of animal growth. Rational use of veterinary drugs can prevent animal diseases, promote animal growth [1], improve the production and quality of animal-origin food, and meet the demand for meat-protein intake. However, driven by economic incentives, over- or extra-label use of veterinary drugs is frequent. This practice leads to excessive residues of various substances, including antibiotics, antiparasitics [2], hormones, and β-agonists-in the edible tissues of food-producing animals. These residues in animal-derived foods pose risks to human health [3,4] and contribute to environmental pollution [5,6]. Ultimately, such practices can also undermine the sustainability of the aquaculture industry itself. Continuous accumulation of veterinary drug residues in the human body beyond a certain concentration is extremely harmful. For example, chloramphenicol can cause aplastic anaemia and leukaemia. Excessively high concentrations of streptomycin can affect brain development in children and may even lead to hearing loss [7]. Excessive concentration of sulphonamide residues may adversely impact the kidneys [8]. Ectoparasite drugs, such as trichlorfon, can cross the blood–brain barrier and are even reported to accumulate in the brains of fish [9]. Clenbuterol can disrupt human hormones and cause arrhythmias, hyperthyroidism, and other toxic symptoms [10]. Hence, strengthening the regulation of drug use in the breeding industry is crucial.

Beyond the intentional addition of veterinary drugs to feed [11], pesticide residues represent another significant risk. During the cultivation of crops used as feed ingredients, banned or excessive pesticides—such as insecticides, fungicides, and herbicides—may be applied to ensure yield. Their residues can transfer into straws and grains, ultimately accumulating in compounded feed. When these residues exceed the Maximum Residue Limit (MRL), they pose direct dangers to human and animal health and may further impact other organisms in the ecosystem through the food chain. Furthermore, if crops are exposed to high temperature, high humidity, or poor ventilation during growth, harvest, storage, or processing, it can promote the proliferation of molds and the subsequent production of mycotoxins [12]. Mycotoxins adversely impact the growth of livestock and can even cause poisoning. When these livestock and poultry containing mycotoxin residues are consumed by humans, the drugs will not be excreted in time and will accumulate in the body. When the toxicity accumulation reaches a certain concentration, it can lead to various pathological reactions in the human body [13,14].

Feed may contain various hazardous substances harmful to livestock and poultry, including prohibited veterinary drugs, pesticide residues, and mycotoxins generated during breeding-related processes. Many countries and organisations have developed various policies to regulate the use of drugs in both animals and crops. The European Union (EU) has established Commission Regulation 37/2010 [15] and lists the MRLs of 511 pesticides commonly used in beef products through EC Regulation 396/2005 [16]. The US Food and Drug Administration has set the MRLs for veterinary drugs in animal-origin foods. The Chinese government had published a list of 151 illegal additives in food and feed, and the Maximum Residue Limits of Veterinary in Food under the National Standards for Food Safety [17]. In the face of such a large number of hazardous substances, it is urgent to develop high-throughput analysis methods to ensure food quality and safety.

A high-throughput analytical method is needed to ensure food quality and safety. At present, many analytical methods have been used to detect drug residues in animal-origin foods. Liquid chromatography-triple quadrupole tandem mass spectrometry (LC-QqQ-MS) is the preferred method for confirmatory analysis because of its high sensitivity and low detection limits. This technique is still considered the gold standard for the multi-residue analysis of targeted pesticides and veterinary drugs [18,19]. However, the use of triple quadrupole mass spectrometry (QqQ) also has some limitations. Because multi-reaction monitoring (MRM) only monitors predefined transitions, it overlooks non-target or unknown analytes, increasing the risk of false negatives. This method tends to overlook compounds not included in the target list, which may lead to false-negative results. In addition, the sensitivity of an MRM method is highly dependent on the dwell time. Consequently, as the number of monitored compounds increases, the dwell time available for each decreases, leading to reduced overall sensitivity [20]. The number of hazardous substances to be monitored is continuously increasing, but the resolution of QqQ remains low and the matrix of feed or livestock meat is complex. As a result, the detection of hazardous substances is easily disturbed by the matrix, leading to false-positive results. In addition, the structure of the compound must be characterised before the MRM method could be used. Moreover, the development of a targeted method is time-consuming and requires the use of standards to optimise the instrument conditions for a specific compound, including transition selections, ion-source voltages, and collision energies. Therefore, it is necessary to develop a new rapid screening method that exhibits high accuracy, high resistance to matrix interference, and high throughput.

High-resolution mass spectrometry (HRMS) has been widely used as a detection technology in recent years. It could simultaneously collect target and non-target mass spectrum data, thereby enabling the simultaneous analysis of an unlimited number of compounds in full-scan mode. During the retrospective evaluation of the data, compounds that were not initially on the target list can be screened without reinjecting the sample. Full-scan detection modes can also provide abundant information for characterising unknown compounds, such as information about the exact mass, retention time, isotope peaks, and fragment ionic intensity, thereby reducing the possibility of false negatives and providing a solid and reliable foundation for establishing more accurate drug-screening methods [21]. Feeds exhibit high diversity and complex matrix compositions. Compared to other commonly used detection methods, HRMS can identify more trace-level compounds in complex mixtures in a single analysis. The technique can provide extremely reliable analysis results without affecting the sensitivity, mass resolution, or quantitative reproducibility of mass spectrometry and can also reduce false negatives. HRMS has already been applied to food safety [22,23], metabolomics [24], forensic toxicology [25,26], clinical diagnosis [27], environmental monitoring [28], structural analysis [29], and component analysis [30,31]. However, only a few HRMS methods have been developed for detecting drug residues in feed. For example, Mehl et al. [32] developed a detection method for 66 multiclass antibiotic residues in animal-origin foods. Luca et al. [33] established a method for detecting four aminoglycoside antibiotics and streptomycin in feed. Currently, most available methods target only a single class of contaminants. However, in practical production, pollutants from different sources—such as pesticides, veterinary drugs, and mycotoxins—can co-exist, making these methods fall short of the need for comprehensive screening. Moreover, existing methods can detect fewer than 200 compounds [34], revealing a lack of high-throughput methods capable of simultaneously covering all these categories. Furthermore, the complexity of the feed matrix itself presents a significant challenge, often leading to unreliable results in the form of false positives and false negatives. Therefore, there is an urgent need to develop high-throughput detection methods that can simultaneously detect multiple hazardous substances, as well as data processing procedures that can reduce false positives or false negatives. In the field of residue analysis, the most widely used are Q-TOF and Orbitrap. Orbitrap provides higher resolution (up to 140,000) and mass accuracy, making it ideal for precise structural elucidation of individual compounds, but its slower scanning speed limits efficiency in multi-contaminant detection. In contrast, Q-TOF offers faster scanning speed, enabling simultaneous qualitative and quantitative analysis in a single run, and is easier to operate for large-scale screening. Thus, for rapid screening of diverse pollutants in feed, Q-TOF demonstrates greater practical efficiency and applicability.

This study aims to develop a novel screening method for 420 hazardous substances commonly found in feed samples, based on the EU 2021/808 and SANTE/11312/2021 standards [35,36]. The selection of 420 compounds for routine screening was based on substances explicitly banned or strictly regulated in feed by the Ministry of Agriculture and Rural Affairs of China, the European Union, and the U.S. FDA. This list also encompasses veterinary drugs frequently abused or overused in farming, as well as pesticide residues and mycotoxins commonly introduced during the cultivation and storage of feed ingredients. The selected hazardous substances include pesticides: Insecticides (e.g., propetamphos, fenthion, trichlorfon, diazinon), fungicides (e.g., thiabendazole), and herbicides (e.g., sodium pentachlorophenate, rimsulfuron), veterinary drugs: antibiotics (e.g., enrofloxacin, ciprofloxacin, tetracycline, tylosin, sulfamethoxazole), anti-inflammatory drugs (e.g., ketoprofen, carprofen, flunixin, meloxicam), hormones (e.g., testosterone, estradiol, progesterone, dexamethasone, prednisolone), β-agonists (e.g., clenbuterol, ractopamine, salbutamol, propranolol), sedatives (e.g., xylazine, diazepam, analgin), and antiparasitics (e.g., albendazole, fenbendazole, closantel, levamisole), and mycotoxins: toxic metabolites produced by molds, such as zearalenone, which can contaminate feed. This comprehensive profile covers prohibited substances, restricted compounds, and high-risk contaminants potentially present throughout the feed supply chain, thereby ensuring the regulatory relevance and practical value of the screening method. The workflow includes the establishment of a database and the acquisition and processing of data. The data are acquired using a non-targeted full-scan mode. The potential hazardous substances in feed samples are screened and confirmed to verify the effectiveness of our method. This study focuses on 6 core feed types with the highest consumption and most concentrated risk exposure in China’s breeding industry, including formula feed, concentrate supplement feed, concentrated feed, composite premix feed, feed additive and feed material. These 6 types of feed are directly related to the safety of major animal-derived foods such as chicken, eggs, pork, and beef. Their matrix characteristics (e.g., high protein in broiler compound feed, high fiber in ruminant concentrate) pose core challenges to the anti-interference ability of detection methods, and they are also key targets for daily sampling and inspection by regulatory authorities. Unlike previous methods which are often limited in scope (targeting either pesticides, veterinary drugs, or mycotoxins alone) or scale (typically <200 compounds), our approach integrates three key innovations: (1) an extensive database covering a wide spectrum of multi-class contaminants; (2) a streamlined “single-step extraction, no purification required” sample preparation protocol designed to minimize analyte loss; and (3) a rigorous two-step “screening-then-confirmation” workflow to effectively balance the minimization of false negatives and false positives.

## 2. Materials and Methods

### 2.1. Materials and Reagents

All the standards for the 420 hazardous substances were purchased from Alta Technology Co., LTD (Tianjin, China). HPLC-grade methanol and acetonitrile (ACN) were obtained from Fisher Scientific (Geel, Belgium). HPLC-grade formic acid was obtained from Dima Technology Inc. (Wuhan, China). Ammonium formate was purchased from Aladdin (Shanghai, China). Ultrapure water was prepared using a Milli-Q system (Milford, MA, USA). The feed samples were obtained from routine checks.

The solid standards were accurately weighed to prepare 1.0 mg/mL standard stock solution using the corresponding solvent, with solvent information provided in Table S1. A liquid standard (single or mixed) was prepared directly by diluting the solvent to a standard stock solution (1.0 mg/mL). The stock solutions were sealed and stored at −18 °C or below in the dark for up to 6 months. Then, accurately remove 1.0 mg/mL of the same type of standard stock solution, 0.10 mL to prepare a mixed standard intermediate solution (10 μg/mL). These solutions were sealed and stored at −18 °C or below in the dark for up to 1 week. Then, we accurately measured 0.5–1 mL of one or more mixed standard intermediate solutions, diluted them to 10 mL using ACN, and used them to prepare a mixed standard working solution (0.5–1 μg/mL). These solutions were stored under −18 °C away from light and were used immediately after preparation.

### 2.2. Sample Preparation

The feed samples were crushed using a grinder (TAISITE, Tianjin, China) and passed through a 0.425 mm aperture to ensure the uniformity of the samples. An accurately weighed 2.0 ± 0.1 g portion of the homogenized sample was transferred into a 50 mL polypropylene centrifuge tube. The extraction was performed by adding 20.0 mL of extraction solvent (acetonitrile/water, 75:25, *v*/*v*) containing 0.1% (*v*/*v*) formic acid. The mixture was immediately vortex-mixed for 1 min (MX-S, Scilogex, Berlin, CT, USA) to ensure complete sample wetting and dispersion, followed by mechanical shaking for 5 min on a horizontal shaker (VX-III, Anjian techology Co., Ltd., Beijing, China). It was followed by sonication for 10 min using an ultrasonic cleaner (50 kHz). The extract was then centrifuged at 8000× *g* for 5 min at 4 °C. The resulting supernatant was carefully collected and filtered through a 0.22-μm nylon syringe filter directly into an LC vial for analysis.

### 2.3. UHPLC–HRMS Analysis

The compounds were separated using UHPLC (Agilent 1290 Infinity, Santa Clara, CA, USA) and Luna Omega Polar C18 (100 mm × 2.1 mm, 1.6 µm) (Agela & Phenomenex) columns. In the positive-ion mode, mobile phase A was an aqueous solution containing 0.1% formic acid and mobile phase B was an ACN solution containing 0.1% formic acid. In the negative-ion mode, mobile phase A was water and mobile phase B was ACN. The elution gradient was as follows: 2% of mobile phase B in the first 0.5 min, which was increased to 15% at 1 min and to 98% at 20 min, maintained constant for 2 min, decreased to 2% at 22.01 min and maintained at that level for 3 min. The injection volume was set to 3 µL using a partial loop mode. The autosampler tray temperature was maintained at 10 °C to ensure sample stability throughout the sequence. The chromatographic separation was performed at a constant temperature of 40 °C. A constant flow rate of 0.4 mL/min was employed throughout the 25 min gradient program. To assess system suitability and retention time stability, a quality control (QC) standard containing 20 representative compounds spanning the entire polarity and retention time range was injected at the beginning and after every 10 samples in the batch (the 20 compounds are the same as those in Table S2). The observed retention time shifts for these QC compounds were less than 0.1 min throughout the sequence, demonstrating excellent chromatographic stability.

Compounds eluted from the UHPLC column were detected using a Sciex 6600 Q-TOF mass spectrometer (Framingham, MA, USA) equipped with electron spray ionisation. The instrument was calibrated every five samples as recommended. Data were acquired in both positive and negative electrospray ionization modes using information-dependent acquisition (IDA). The full-scan MS survey (TOF-MS) was performed over the mass range of *m*/*z* 50–1000. The top 10 most intense ions exceeding 500 cps from the survey scan were selected for subsequent MS/MS fragmentation. The MS/MS experiments were performed using a collision energy spread of 20, 40, and 60 V, providing comprehensive fragment ion information for confirmation. The scan time for full-scan mode was set to 0.1 s per scan to ensure each chromatographic peak contained at least 5 data points, meeting the requirements for peak identification; the MS/MS scan time was 0.05 s per scan, ensuring the integrity of fragment ion information without missing target ions. The instrument was calibrated every five injections using an automated calibration delivery system (CDS) with a proprietary mixture to maintain mass accuracy below 5 ppm.

### 2.4. Data Processing

The data were processed using Sciex OS software (version 2.1). Peak finding parameters were set as follows: minimum peak width of 3 s, noise threshold of 1000 counts, and S/N threshold of 3. Gaussian smoothing was applied when necessary to improve peak shape. For co-eluting peaks, the chromatographic deconvolution function of the software was utilized, primarily based on differences in accurate mass and fragment ion spectra. After setting parameters, the entire workflow could be run automatically and provide an Excel table including *m*/*z*, RT, and peak response of extracted compounds for further differential analysis. The screening phase employed lenient criteria: a mass tolerance of 10 ppm for precursor ions and 20 ppm for fragment ions, to minimize false negatives. Suspect peaks meeting these criteria were flagged as “potential positives.” For confirmation, stricter criteria were applied: mass tolerance of 5 ppm (precursor) and 10 ppm (fragment), retention time matching within ±0.2 min, and fragment ion abundance ratios within allowed deviations. Only peaks satisfying all confirmation criteria were reported as positively identified. Detailed screening and confirmation conditions are described in Section 3.1.6.

### 2.5. Method Validation

To align with the EU SANTE/11312/2021 [36] guidelines for qualitative screening methods, the validation assessed the SDL and accuracy. It is explicitly noted in the guideline that analyte recovery is not a requirement under such a context. To uphold a more comprehensive standard of scientific rigor, we voluntarily included an assessment of other critical validation metrics, namely the LOD, precision, ME, and stability.

To evaluate the LOD, target compounds diluted to 5, 10, 50, and 100 μg/L were detected, respectively. The lowest detectable concentration (95% confidence limit) was used as the LOD of the compound. 10, 50, 100 and 500 μg/L of the target compound were added to the blank feed matrix (*n* = 12). The minimum concentration that can be screened and confirmed was used as the SDL for compounds in the matrix, each in triplicate. To determine intra- (*n* = 3) and inter-day (*n* = 3, 3 days) precisions, mixed standard working solutions of 50, 100, and 500 μg/L (1, 2, and 10 times the SDL) were added to each sample, respectively. Precision was expressed as the relative standard deviation (RSD, %).

To investigate the ME, we examined the signal intensity of the drug in six different matrix samples (*n* = 3). ME was evaluated by comparing the peak areas of target compounds in post-extraction spiked matrix samples to those in pure solvent at equivalent concentrations. To investigate the stability of the compounds, the standard stock solution of 1.0 mg/mL was stored at −18, 4, and 18 °C for one, three, and six months, respectively, and then prepared into 50 µg/L of a mixed standard working solution. Next, 10 μg/mL of the mixed standard intermediate solution was stored at −20 °C for one week. After the detection, the concentration difference was calculated based on the detection results (*n* = 3, per storage condition).

According to the SANTE-11312-2021 [37], the validation of a screening method based on an SDL can be focused on detectability. For each commodity group, a basic validation should involve analysis of at least 20 samples spiked at the estimated SDL. Therefore, to evaluate the accuracy of the method, we randomly selected 20 hazardous substances and spiked them into 20 different feed samples, respectively. The sample information is shown in Table S2. Each compound was spiked at the level of its SDL, and the detection rate for each compound was calculated.

### 2.6. Real Sample Collection

To evaluate the reliability and practicability of the method, we collected a total of 248 feed samples from different business premises and warehouses across the country. Including feed materials, vitamin premixed feed, trace element premixed feed, composite premix feed, concentrated feed, and feed additives. In addition, the samples covered 10 provinces in China, which has reference significance for the evaluation of feed samples in the whole country. The sample information is shown in Table S3, and the hazardous substances in the samples were detected using the established UHPLC-HRMS method. Samples were kept in airtight bags after grinding and stored at −20 °C until analysis.

## 3. Results

### 3.1. Development and Optimization Process of the Method

The method combines primary and secondary mass spectral information for the screening and confirmation of 420 hazardous substances by comparing information such as retention times, primary and secondary ion mass-to-charge ratios (*m*/*z*), and fragment ion relative ion abundances with those in databases.

#### 3.1.1. Establishment of a Database

First, from the regulatory lists of drugs that are added illegally, drugs that are often abused in different types of feed, and compounds that may be harmful to humans or animals detected through screening, we selected 420 potential hazardous substances that may be found in feed. We prepared a mixed standard working solution (0.5–1 μg/mL) of each compound and detected by UHPLC–HRMS using established methods to obtain primary and secondary mass spectral information. Detailed information on the 420 compounds is presented in Table S1.

A database was established for the identification of the potential hazardous substances using LibraryView software (1.0). This database mainly included the retention time, common name, molecular formula, CAS number, exact *m*/*z* of the parent ion and at least two characteristic fragment ions, and the relative abundance ratio of the characteristic fragment ions. Among them, the adduct with the highest signal intensity was selected as the protonated molecule as the precursor ion of MS2 experiment. After fragmentation of the precursor ions, all fragment ions were added to the database (Table S1 presents the top 3 fragment ions with the highest signal intensity). To minimize the risk of false negatives, secondary mass spectra were obtained for each compound at collision energies of 20, 40 and 60 V, respectively. The database includes several isomeric compounds. Differentiation was primarily based on retention time matching and characteristic fragment ion patterns. In cases where isomers co-elute or exhibit similar fragmentation, confirmation with authentic standards or orthogonal techniques (e.g., NMR) is recommended.

#### 3.1.2. Optimisation Process of Sample Preparation

**Figure 1:**
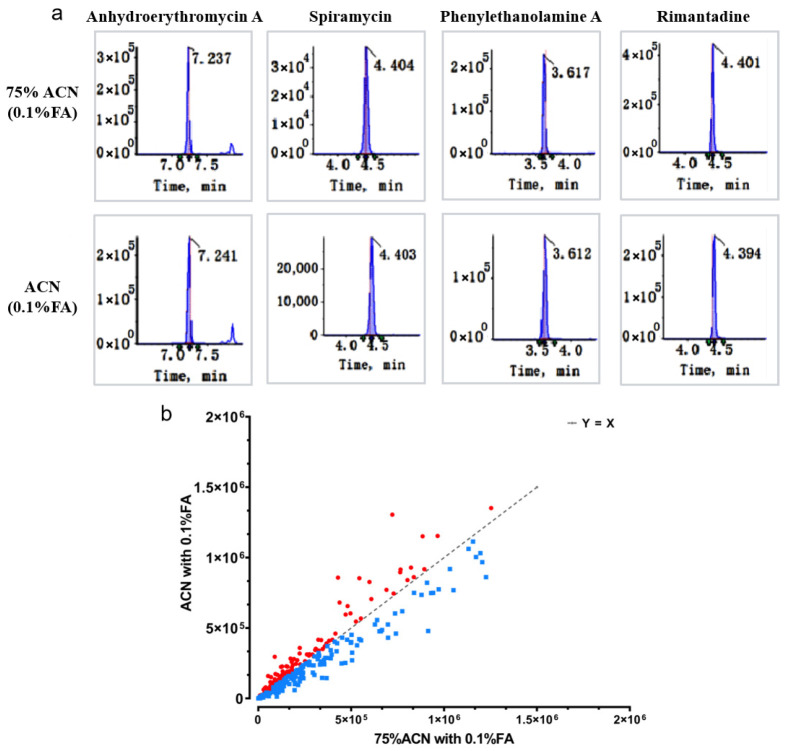
The compounds were detected in different extraction solvents (**a**) (top: 75% ACN containing 0.1% FA, bottom: ACN containing 0.1% FA) and statistical results (**b**) (Each point represents a target compound. The *X*-axis represents the response intensity in 75% ACN with 0.01% FA, and the *Y*-axis represents the response intensity in ACN with 0.01%FA. Blue points represent higher responses in 75% ACN with 0.01% FA, and red points represent higher responses in ACN with 0.01% FA).

To meet the requirements of high-throughput screening, the extraction solvent should be able to extract compounds of different polarities while avoiding co-extraction of matrix-interfering components. We first selected ACN, which has better solubility and could be used for a relatively wide range of polar targets. A certain amount of water in the extraction solution is conducive to the dispersion of the matrix, which enhances extraction efficiency. In addition, a higher proportion of the organic phase is beneficial for the precipitation of proteins in the matrix. Therefore, we compared three extraction solvents: 50% ACN, 75% ACN, and ACN containing 0.1% FA. After extraction with 50% ACN, fatty substances precipitated when the sample was stored at 4 °C for 12 h. The chromatographic peaks were extracted, and the signal intensity was calculated by Sciex OS software. The results showed that, compared with pure ACN, the signal strength of 236 compounds in 75% ACN is higher than that in ACN., such as anhydroerythromycin A, spiramycin, phenylethanolamine A, and rimantadine (Figure 1a). We measured the signal intensities of these compounds in 75% ACN and ACN extracts containing 0.1% FA, and found that 344 compounds exhibited a higher signal intensity in the 75% ACN extract than they did in the ACN extract. In summary, we selected 75% ACN with 0.1% FA as the extraction solution (Figure 1b). The signal intensity of 344 compounds in 75% ACN was increased by an average of 28.6% (range: 12.3–56.8%) compared to pure ACN.

#### 3.1.3. Selection of Columns

**Figure 2:**
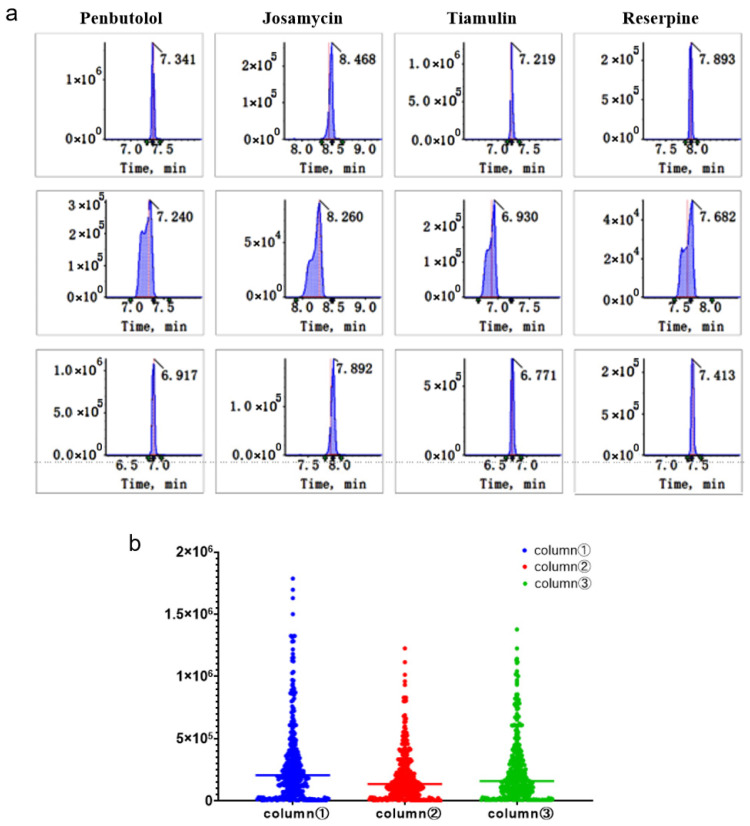
The compounds were detected in different chromatographic columns (**a**) and statistical results (**b**) (Each point represents a target compound; Blue, red, and green dots correspond to columns 1, 2, and 3, respectively. The *Y*-axis shows the peak areas of the compounds, and the distribution of points reflects the differences in the response of the compounds to the different columns). column 1: Luna Omega Polar C18 (100 mm × 2.1 mm, 1.6 μm); column 2: Eclipse Plus C18 (100 mm × 2.1 mm, 1.8 μm); column 3: Kinetex Polar C18 (150 mm × 2.1 mm, 2.6 μm).

The selection of appropriate columns is crucial for the analysis of compounds. To choose the most suitable column, we compared the peak shapes and responses of the compounds across different types and manufacturers of columns. We analysed 420 compounds using three different C18 columns: Eclipse Plus C18 (100 mm × 2.1 mm, 1.8 μm, Agilent, Santa Clara, CA, USA), Luna Omega Polar C18 (100 mm × 2.1 mm, 1.6 μm, Phenomenex, Torrance, CA, USA), and Kinetex Polar C18 (150 mm × 2.1 mm, 2.6 μm, Phenomenex, Torrance, CA, USA). In the Eclipse Plus C18 column, some compounds, such as penbutolol, josamycin, tiamulin, and reserpine, did not exhibit a well-formed peak (Figure 2a). The signal intensity of compounds was higher in the Luna Omega Polar C18 column than it was in the Kinetex Polar C18 column. Based on the statistical results shown in Figure 2b, the Luna Omega Polar C18 column (100 mm × 2.1 mm, 1.6 μm) was selected as the unified chromatographic column for the simultaneous analysis of all 420 target compounds in the final method. This selection provided the best overall performance in terms of peak shape and response across the entire analyte panel.

#### 3.1.4. Selection of Mobile Phase

We compared the separation of the 420 compounds using four different mobile phases: water-ACN containing 0.1% FA, water-ACN containing 0.5 mmol/L ammonium formate, water-methanol containing 0.1% FA, and water-methanol containing 0.5 mmol/L ammonium formate. It was found that, the retention time of the compound was uniform in water and ACN. Some compounds, such as flucloxacillin, were not retained in the water-methanol mobile phase system, while cephalosporins and antibiotics were unstable in methanol and underwent irreversible alcoholysis. Therefore, we selected water and ACN as the mobile phases. We compared two modifiers, 0.1% formic acid and 0.5 mmol/L ammonium formate. Both of these modifiers afforded similar detection results for the compounds, because there was no significant difference in the shape and intensity of the compounds in the two mobile phases. Finally, we selected formic acid as the mobile phase modifier.

#### 3.1.5. Selection of Purification Materials

We compared the purification effects of five purification materials on feed samples: 50 mg C18, 10 mg PC, 50 mg C18, 50 mg PSA, Agilent Captiva EMR-Lipid, and Phenomenex Strata-X PRO. However, the purification effectiveness was not significant in relation to that of the unpurified samples. The signal intensity obtained using the direct dilution method was generally higher than that obtained using the five purification methods. By sequentially comparing the target compounds, we found that some of the target compounds could not be detected after purification, including six β-receptor agonist compounds (cimaterol, clenbuterol, tobuterol, penbuterol, propranolol, and clenpropro). This could be because the feed sample matrices were too complex, causing the loss of target compounds while removing impurities. After careful consideration, we decided not to purify the samples to process them.

**Figure 3:**
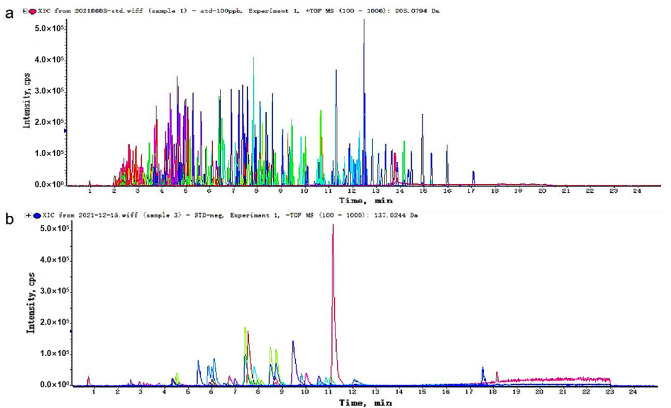
Extraction ion chromatograms (EIC) of 384 hazardous substances in positive ion mode (**a**) and 36 hazardous substances in negative ion mode (**b**). Each color peak represents a compound. Due to the large number of compounds, the colors of some compounds are quite similar. Some peaks overlap, but they can still be characterized based on the exact mass.

Following the optimization of the sample preparation and analytical conditions, this method successfully separated the 420 hazardous substances within a 30 min chromatographic run. The detection results demonstrated well-resolved separation for the majority of compounds, with symmetrical and sharp ion peaks, underscoring the high-throughput capability of the method. Among them, 373 compounds were analyzed in positive ion mode, encompassing most veterinary drugs (e.g., antibiotics, hormones, antiparasitics) and some pesticides. The remaining 47 compounds were analyzed in negative ion mode, including acidic non-steroidal anti-inflammatory drugs, mycotoxins (e.g., zearalenones), and other acidic pharmaceuticals. The screening and identification of these hazardous substances were successfully accomplished by matching their mass-to-charge ratios (*m*/*z*) and retention times against the established database. Figure 3 presents the extracted ion chromatograms (XICs) demonstrating the detection of all 420 target compounds.

#### 3.1.6. Screening and Confirmation Process

By referring to the ‘Guidance document on analytical quality control and method validation procedures for pesticide residues and analysis in food and feed’ and other technical specification documents, we established a screening and confirmation process for hazardous substances. To avoid false-negative results during sample screening, the extraction window of the primary parent ions was adjusted to 10 ppm and that of the secondary fragment ions was adjusted to 20 ppm. Total ion chromatograms (TICs) were extracted using the full-scan mode. Discernible chromatographic peaks were selected and contrasted with the compound information available in the compound library. When the comparison results meet the following conditions, a suspected positive sample can be considered to be detected. (1) The peak of a single target compound is not less than five sampling points. (2) When the signal-to-noise ratio (S/N) exists, the S/N should be ≥3. In the absence of S/N, the detection limit is the lowest detectable concentration of the compound at the 95% confidence level. (3) When the *m*/*z* of a primary parent ion is ≥200, the relative deviation should be ≤10 ppm. When it is <200, the absolute deviation should be <1 mDa.

After a suspected positive sample was screened, control experiments were performed using reference standards whose concentration should be close to that of the target compound in the sample. If a blank matrix similar to that of the sample is available, the compounds should be added to the blank matrix for the experiment. The mass extraction window adjusted to the primary parent ion was 5 ppm and the secondary fragment ion was 10 ppm. The TIC was extracted using the full-scan mode and discernible chromatographic peaks were selected. A positive sample is confirmed to have been detected if the results of the controlled experiment meet the following requirements. (1) The retention time of the compound should be more than twice the dead time. The absolute deviation of the retention time between the target and reference standards under the same conditions should be no more than 0.2 min. (2) The relative deviation of the primary parent ion *m*/*z* should not exceed 5 ppm and that of the secondary fragment ion *m*/*z* should not exceed 10 ppm. When the *m*/*z* of a primary parent ion is less than 200, the absolute deviation should be less than 1 mDa. (3) The confirmed sample should be adjusted to the appropriate injection concentration to ensure that the major isotope peaks can be measured, and that the *m*/*z* of the isotope peaks should meet the quality accuracy requirements of the target confirmation. (4) Of the secondary fragments detected on the mass spectrum, two or more major fragment ions meet the aforementioned mass accuracy requirements. Their relative ion abundance ratio also meets the maximum allowable deviation requirements (when the relative abundance of fragment ions was ≥50%, the maximum allowable deviation was 20%. If it was >20% and <50%, the maximum allowable deviation was 25%. If it was >10% and <20%, the maximum allowable deviation was 30%. If the deviation is ≤10%, the maximum allowable deviation is 50%). Sample detection is considered negative when these conditions are not met.

### 3.2. Method Validatio n

**Figure 4:**
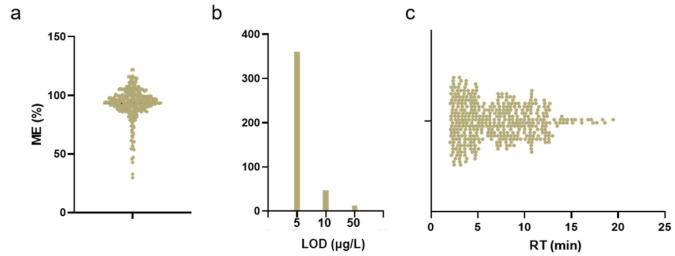
Overview plots for ME distribution (**a**), LOD distribution (**b**), and retention time distribution (**c**).

The method’s performance parameters are summarized in Tables S4 and S5. The LODs were established at three levels: 5 μg/L for 361 compounds, 10 μg/L for 47 compounds, and 50 μg/L for 12 compounds. For the SDLs were 50 μg/L (359 compounds), 100 μg/L (47 compounds), and 500 μg/L (14 compounds). The inter-day precision, expressed as the average coefficient of variation (CV), was <25%, 15%, and 10% at concentrations of 50, 100, and 500 μg/L, respectively. The intra-day CV was less than 10% for all compounds at all concentration levels. Further details are presented in Table S4. The ME was calculated as the ratio of the signal strength of the compounds in the solvent to that in different matrix samples. The ME of the 420 compounds ranged from 36.12% to 121.16%. More than 80% of the compounds had an ME of >60%. To verify the stability of these compounds, we stored the standard reserve solution (1.0 mg/mL) in different environments for 1–6 months and prepared it into a mixed standard working solution (50 µg/L) for detection. The detected concentrations were within the ±5% range. Then, the mixed standard intermediate solution (10 μg/mL) was stored at −20 °C for one week and used to prepare a mixed standard working solution (50 µg/L) for detection. The detected concentration was again in the range of ±5%. The solution was used within the validity period. The stability-evaluation results are listed in Table S5. In the accuracy assessment, all 20 target compounds were successfully detected in all 20 different matrices, and, importantly, no false-negative or false-positive results were observed, demonstrating the good accuracy of the method. The detection results of the compounds in 20 matrices are shown in Figure S1. The overview diagrams of the ME distribution, LOD distribution, and retention time distribution are shown in Figure 4. The validated method meets all criteria set by the newly issued GB/T 23182-2025 [37], which is scheduled for imminent implementation.

### 3.3. Real Sample Analysis

To further evaluate the reliability and practicality of the method, we collected 248 feed samples from diverse business premises and warehouses across China. These samples covered six categories to reflect real-world feed matrix diversity. Samples were detected as described above. Hazardous substances were detected in 53 samples, and up to 5 hazardous substances were detected in a single sample. 23 samples detected no less than two hazardous substances. Among them, the most detected hazardous substances were acetoquine and albendazole, which were mainly used for antibacterial and insecticidal activities in livestock and poultry. The detailed results are shown in Table S6. Notably, according to the “Catalog of Feed Additives” issued by the Ministry of Agriculture and Rural Affairs of the People’s Republic of China, neither acetoquine nor albendazole is approved for use as a feed additive. Therefore, their detection in any feed product constitutes prohibited addition. Analysis of the detection pattern suggests that the relatively high frequency of detection in Compound Premix Feed (a high-concentration core component used by farms to formulate complete feed) and Feed Material strongly implies purposeful addition, likely intended to achieve therapeutic effects such as deworming (albendazole) or control of diarrhea/growth promotion (acetoquine) via core ingredient carriers. Furthermore, among the 23 samples containing multiple hazardous substances, co-occurrence of albendazole with antibiotics such as tilmicosin was observed, reflecting the complex reality of potential concurrent use of multiple prohibited or restricted substances in farming practice. A quality control sample was inserted after every 10 samples. Through the analysis of RT, mass accuracy and response intensity in quality control samples, it was shown that the instrument is in good condition during the whole operation process. The results show that the established method is suitable for high-throughput screening of 420 hazardous substances in feed products.

## 4. Discussion

The UHPLC-Q-TOF HRMS method developed in this study enables the high-throughput screening and confirmation of 420 diverse hazardous substances in feed, filling a critical gap in comprehensive feed safety analysis. Compared to previous HRMS-based feed analysis methods, the advantages of our approach are threefold. First, in terms of compound coverage, our method includes 420 target substances–surpassing the 66 antibiotics covered by Mehl et al. [32] and the 22 prohibited veterinary drugs by Wang et al. [11]. Notably, we included different types of hazardous substances, including pesticides, veterinary drugs and mycotoxins, addressing the risk of “missed detection” caused by illegal additives introduced through different routes. Moreover, the in-house database, containing retention times, exact masses, characteristic fragments, and their relative abundances, is readily expandable with new reference standards, allowing continuous updates to align with evolving regulatory requirements.

Second, the sample preparation protocol was strategically simplified to a single-step extraction using 75% acetonitrile with 0.1% formic acid, deliberately omitting a cleanup step. This decision was validated by our findings that common purification sorbents led to significant and unacceptable losses of several critical analytes, such as specific β-receptor agonists. Consequently, this streamlined approach not only reduces time and cost but also enhances the overall recovery rates for a wider spectrum of compounds. In addition, the method allows simultaneous screening of 420 compounds within a single 30 min chromatographic run, with sample preparation requiring approximately 30 min per sample (including extraction, centrifugation, and filtration). Compared to other multi-class sequential detection approaches, this workflow reduces the total analysis time by about 60% and decreases reagent costs per sample by approximately 40% [38], making it suitable for high-throughput screening of large feed sample batches.

Third, the analytical workflow, which employs a sequential screening-then-confirmation process with tailored stringency criteria, effectively minimizes the risk of false negatives–a critical concern in food safety screening. This was achieved by initially applying less restrictive parameters to capture potential positives, followed by rigorous confirmation using precise mass accuracy, isotopic pattern matching, and fragment ion criteria. The accuracy of compound detection was greatly improved, and the false positive rate and false negative rate were reduced [39].

The practical applicability of the method was validated using 248 market samples covering 6 major feed categories in China, with 53 positive samples (21.4% positive rate). The most frequently detected substances–acetoquine (12 samples) and albendazole (9 samples)–are widely used for antibacterial and anthelmintic purposes in livestock and poultry, but their excessive residues pose risks: acetoquine metabolites can induce hepatocellular damage in humans via long-term exposure [8], while albendazole residues may cause reproductive toxicity in sensitive populations [10]. Importantly, our method detected co-occurrence of 2–5 hazardous substances in 23 samples (e.g., Albendazole + Tilmicosin in composite premix feed), highlighting the need for multi-residue screening–a capability that single-class residue methods (e.g., mycotoxin–only HPLC methods [13,14]) cannot fulfill. This finding aligns with the global trend of “integrated risk assessment” in feed safety (e.g., the EU’s “Farm to Fork” strategy), where our method can serve as a core tool for large-scale surveillance, enabling rapid identification of high-risk feed batches and traceability of contamination sources.

Despite its robustness, the study acknowledges certain limitations. The discrimination of isomeric compounds or co-eluting analytes remains challenging based solely on HRMS data, necessitating confirmation with authentic standards or orthogonal techniques such as nuclear magnetic resonance (NMR) spectroscopy. Moreover, the 75% ACN–0.1% FA extraction system exhibited suboptimal efficiency for some strongly polar or weakly ionizable compounds, suggesting a potential avenue for future optimization. Investigations into alternative extraction solvents, pH adjustments, or the use of ion-pairing reagents could further extend the method’s applicability. In the negative ion mode, the mobile phase consisted of water (A) and acetonitrile (B) without pH adjustment. It should be noted that the lack of pH control may affect the retention time reproducibility for ionizable compounds, such as acidic or basic analytes, potentially leading to reduced inter-day and batch-to-batch precision. This aspect should be considered when adapting the method to other matrices or instruments. Moreover, quantification of the identified compounds was not performed by this method. Subsequent quantitative analysis necessitates the use of authentic standards to establish a calibration curve.

## 5. Conclusions

In this study, a high-throughput method based on UHPLC-Q-TOF high-resolution mass spectrometry was successfully developed and validated for the simultaneous screening and confirmation of 420 hazardous substances, including pesticides, veterinary drugs, and mycotoxins, in feed. The method utilized an optimized “single-step extraction, no purification required” sample preparation protocol and a “screening-then-confirmation” data processing strategy, effectively addressing the dual challenge of false negatives and false positives in complex feed matrices. Method validation demonstrated that its performance in terms of LOD, SDL, precision, accuracy, and stability met the requirements for high-throughput screening.

The establishment of the method has made a significant contribution to ensuring the quality and safety of feed. First, it significantly expands the scope of simultaneous screening, increasing the number of target compounds from the tens or hundreds typically reported in the literature to 420, thereby providing the feed industry with its first truly broad-spectrum risk monitoring tool. Second, the established expandable database and systematic workflow offer regulatory authorities a flexible and powerful technological platform to address the future challenge of emerging illegal additives. Finally, in practical application, the method successfully identified 53 positive samples out of 248 market samples and revealed the co-occurrence of multiple hazardous substances. This finding provides critical data and practical evidence for implementing “integrated risk assessment,” directly serving feed and food safety strategies in China and globally.

Nevertheless, this study has certain limitations: Firstly, the HRMS data have a limited capability to distinguish co-eluting isomeric compounds; unambiguous identification in such cases still relies on authentic standards or orthogonal techniques like NMR. Secondly, the current 75% acetonitrile extraction system might exhibit suboptimal efficiency for some strongly polar or weakly ionizable compounds, indicating that the sample preparation protocol has room for further optimization. Furthermore, this study primarily focused on qualitative screening; accurate subsequent quantification requires the establishment of calibration curves using corresponding authentic standards, and the introduction of internal standards will be a key measure to improve the accuracy and reliability of quantitative results (especially for matrix effect correction).

This method provides a reliable platform for high-throughput screening of multi-class hazardous substances in feed. Future work will focus on three key directions: expanding method coverage by optimizing extraction systems (e.g., introducing ion-pairing reagents or pH adjustment) to improve recovery of strongly polar compounds; integrating this screening workflow with rapid quantification techniques, including the introduction of isotope internal standards for matrix effect correction, to establish an accurate and reliable calibration-based quantification method; and continuously updating the database while exploring intelligent data processing algorithms (e.g., machine learning-assisted peak annotation) to address emerging contaminants. These enhancements will further strengthen the feed safety risk monitoring system and contribute to global food safety governance.

## Data Availability

The data presented in this study are available on request from the corresponding author. The data are not publicly available due to Confidentiality.

## Supplementary Materials

The following supporting information can be downloaded at: https://www.mdpi.com/article/10.3390/foods15030502/s1, Figure S1. The XIC of 20 compounds in different matrices. (a,b): Pig formula feed; (c,d): Chicken formula feed; (e,f): Pig concentrated feed; (g,h): Chicken concentrated feed; (i,j): Cow concentrate supplement (k,l): Composite additive premix; (m,n): Food flavour; (o,p): Plant extracts (Radix Astragali); (q,r): Trace mineral feed; (s,t): Vitamin premix feed. Table S1. Retention time, Adduct, and mass-to-charge ratios of 420 risk substances. Table S2. Information of 20 compounds and 20 matrices. Table S3. Information of 248 real samples. Table S4. SDL and Precision test results of 420 risk substances. Table S5. Stability test results of standard reserve solution and mixed standard intermediate solution of 420 risk substances. Table S6. Information on samples containing risk substances.

## References

[1] Kirchhelle C. (2018). Pharming animals: A global history of antibiotics in food production. Palgrave Commun..

[2] Stolker A.A., Brinkman U.A. (2005). Analytical strategies for residue analysis of veterinary drugs and growth-promoting agents in food-producing animals—A review. J. Chromatogr. A.

[3] Lehmann S., Thomas A., Schiwy-Bochat K.H., Geyer H., Thevis M., Glenewinkel F., Rothschild M.A., Andresen-Streichert H., Juebner M. (2019). Death after misuse of anabolic substances (clenbuterol, stanozolol and metandienone). Forensic Sci. Int..

[4] Verma T., Aggarwal A., Singh S., Sharma S., Sarma S. (2022). Current challenges and advancements towards discovery and resistance of antibiotics. J. Mol. Struct..

[5] Gehring R., Baynes R.E., Riviere J.E. (2006). Application of risk assessment and management principles to the extralabel use of drugs in food-producing animals. J. Vet. Pharmacol. Ther..

[6] Boobis A., Cerniglia C., Chicoine A., Fattori V., Lipp M., Reuss R., Verger P., Tritscher A. (2017). Characterizing chronic and acute health risks of residues of veterinary drugs in food: Latest methodological developments by the joint FAO/WHO expert committee on food additives. Crit. Rev. Toxicol..

[7] Kong C.T., Holt D.E., Ma S.K., Lie A.K., Chan L.C. (2000). Effects of antioxidants and a caspase inhibitor on chloramphenicol-induced toxicity of human bone marrow and HL-60 cells. Hum. Exp. Toxicol..

[8] Qiu S.S. (2023). Study on the status and influence of sulfonamides residues in animal food. Food Saf. Guide.

[9] Shi Q., Yang H., Chen Y., Zheng N., Li X., Wang X., Ding W., Zhang B. (2023). Developmental Neurotoxicity of Trichlorfon in Zebrafish Larvae. Int. J. Mol. Sci..

[10] Huang D.S., Yuan C.J., Yan X.D. (2018). Discussion on the causes and prevention and control measures of drug residues in animal products. Anhui Agric. Sci. Bull..

[11] Wang B., Xie K., Lee K. (2021). Veterinary Drug Residues in Animal-Derived Foods: Sample Preparation and Analytical Methods. Foods.

[12] Huang Q., Guo W., Zhao X., Cao H., Fan K., Meng J., Nie D., Wu Y., Han Z. (2022). Universal screening of 200 mycotoxins and their variations in stored cereals in Shanghai, China by UHPLC-Q-TOF MS. Food Chem..

[13] Alshannaq A., Yu J.H. (2017). Occurrence, Toxicity, and Analysis of Major Mycotoxins in Food. Int. J. Environ. Res. Public Health.

[14] Rai A., Das M., Tripathi A. (2020). Occurrence and toxicity of a fusarium mycotoxin, zearalenone. Crit. Rev. Food Sci. Nutr..

[15] European Commission (2010). Commission Regulation (EU) No 37/2010 of 22 December 2009 on Pharmacologically Active Substances and Their Classification Regarding Maximum Residue Limits in Foodstuffs of Animal Origin.

[16] European Commission (2005). Commission Regulation 396/2005 on Maximum Residue Levels of Pesticides in or on Food and Feed of Plant and Animal Origin and Amending Council Directive 91/414/EEC.

[17] (2019). National Food Safety Standard Maximum Residue Limits for Veterinary Drugs in Foods.

[18] Turnipseed S.B., Storey J.M., Lohne J.J., Andersen W.C., Burger R., Johnson A.S., Madson M.R. (2017). Wide-Scope Screening Method for Multiclass Veterinary Drug Residues in Fish, Shrimp, and Eel Using Liquid Chromatography-Quadrupole High-Resolution Mass Spectrometry. J. Agric. Food Chem..

[19] Hayward D.G., Wong J.W., Shi F., Zhang K., Lee N.S., DiBenedetto A.L., Hengel M.J. (2013). Multiresidue pesticide analysis of botanical dietary supplements using salt-out acetonitrile extraction, solid-phase extraction cleanup column, and gas chromatography-triple quadrupole mass spectrometry. Anal. Chem..

[20] Hermes N., Jewell K.S., Wick A., Ternes T.A. (2018). Quantification of more than 150 micropollutants including transformation products in aqueous samples by liquid chromatography-tandem mass spectrometry using scheduled multiple reaction monitoring. J. Chromatogr. A.

[21] Pang X., Qiu J., Zhang Z., Li P., Xing J., Su X., Liu G., Yu C., Weng R. (2023). Wide-Scope Multi-residue analysis of pesticides in beef by gas chromatography coupled with quadrupole Orbitrap mass spectrometry. Food Chem..

[22] Hou X., Xu X., Xu X., Han M., Qiu S. (2020). Application of a multiclass screening method for veterinary drugs and pesticides using HPLC-QTOF-MS in egg samples. Food Chem..

[23] Wang J., Zhao W., Guo W., Li Y., Jiang R., Li H., Wang S., Li Z. (2022). Simultaneous screening and analysis of 155 veterinary drugs in livestock foods using ultra-high performance liquid chromatography tandem quadrupole linear-ion-trap mass spectrometry. Food Chem..

[24] Guijas C., Montenegro-Burke J.R., Warth B., Spilker M.E., Siuzdak G. (2018). Metabolomics activity screening for identifying metabolites that modulate phenotype. Nat. Biotechnol..

[25] Yu J.S., Seo H., Kim G.B., Hong J., Yoo H.H. (2019). MS-Based Molecular Networking of Designer Drugs as an Approach for the Detection of Unknown Derivatives for Forensic and Doping Applications: A Case of NBOMe Derivatives. Anal. Chem..

[26] Szeremeta M., Pietrowska K., Niemcunowicz-Janica A., Kretowski A., Ciborowski M. (2021). Applications of Metabolomics in Forensic Toxicology and Forensic Medicine. Int. J. Mol. Sci..

[27] Wang Q., Su B., Dong L., Jiang T., Tan Y., Lu X., Liu X., Lin X., Xu G. (2020). Liquid Chromatography-Mass Spectrometry-Based Nontargeted Metabolomics Predicts Prognosis of Hepatocellular Carcinoma after Curative Resection. J. Proteome Res..

[28] Qiao B., Nie S., Li Q., Majeed Z., Cheng J., Yuan Z., Li C., Zhao C. (2022). Quick and In Situ Detection of Different Polar Allelochemicals in Taxus Soil by Microdialysis Combined with UPLC-MS/MS. J. Agric. Food Chem..

[29] Ferreira H.B., Barros C., Melo T., Paiva A., Domingues M.R. (2022). Looking in Depth at Oxidized Cholesteryl Esters by LC-MS/MS: Reporting Specific Fragmentation Fingerprints and Isomer Discrimination. J. Am. Soc. Mass Spectrom..

[30] Chen Q., Shi J., Mu B., Chen Z., Dai W., Lin Z. (2020). Metabolomics combined with proteomics provides a novel interpretation of the changes in nonvolatile compounds during white tea processing. Food Chem..

[31] Qu F., Zhu X., Ai Z., Ai Y., Qiu F., Ni D. (2019). Effect of different drying methods on the sensory quality and chemical components of black tea. LWT-Food Sci. Technol..

[32] Mehl A., Schmidt L.J., Schmidt L., Morlock G.E. (2021). High-throughput planar solid-phase extraction coupled to orbitrap high-resolution mass spectrometry via the autoTLC-MS interface for screening of 66 multi-class antibiotic residues in food of animal origin. Food Chem..

[33] Luca F., Carlos G., Joerg S., Christoph H., Luciano P., Ursula V. (2024). Determination of four aminoglycoside antibiotics and spectinomycin in feed at cross-contamination level: Development and in-house validation of a LC–MS/MS method for enforcing EU Regulation. J. Pharm. Biomed. Anal..

[34] Ingrid D.S., Paul Z., Ionara R., Roger W., Hans M. (2024). Multi-residue determination of biocides in dairy products and slurry feed using QuEChERS extraction and liquid chromatography combined with high resolution mass spectrometry (LC-ESI-QOrbitrap™-MS). Food Chem..

[35] (2002). Commission Decision of 12 August 2002 implementing Council Directive 96/23/EC concerning the performance of analytical methods and the interpretation of results (2002/657/EC). Off. J. Eur. Communities.

[36] (2021). Guidance document on analytical quality control and method validation procedures for pesticide residues and analysis in food and feed (SANTE/11312/2021). Off. J. Eur. Communities.

[37] (2025). Guideline for Method Development, Validation Verification and Internal Quality Control in Feed Quality and Safety Detection.

[38] Wang X., Liu Y., Su Y., Yang J., Bian K., Wang Z., He L.M. (2014). High-throughput screening and confirmation of 22 banned veterinary drugs in feedstuffs using LC-MS/MS and high-resolution Orbitrap mass spectrometry. J. Agric. Food Chem..

[39] Dasenaki M.E., Bletsou A.A., Koulis G.A., Thomaidis N.S. (2015). Qualitative Multiresidue Screening Method for 143 Veterinary Drugs and Pharmaceuticals in Milk and Fish Tissue Using Liquid Chromatography Quadrupole-Time-of-Flight Mass Spectrometry. J. Agric. Food Chem..

