# Supplementary material for: High-Throughput Screening and Confirmation of 420 Hazardous Substances in Feed Based on Liquid Chromatography−High-Resolution Mass Spectrometry
Source: Foods. 2026 Feb 1;15(3):502. doi: 10.3390/foods15030502 (PMC12896493; doi:10.3390/foods15030502)
Supplement: Supplementary file 1 [file foods-15-00502-s001.zip › foods-3965837-supplementary.pdf]

Table S1. Retention time, Adduct, and mass-to-charge ratios of 420 risk substances.

Number	Compounds	CAS	Formula	RT（min）	Adduct	Parent ion（m/z）	Fragment ions（m/z）	Solvent
1	2-Aminoflubendazole	82050-13-3	$C_{14}H_{10}FN_3O$	4.27	[M+H]+	256.0881	123.0232/95.0286/133.0629	DMSO
2	2-Methyl-5-nitroimidazole	696-23-1	$C_4H_5N_3O_2$	2.17	$[M+H]^+$	128.0455	82.0531/98.0474/111.0425	ACN
3	2-NP-SEM	16004-43-6	$C_8H_8N_4O_3$	4.67	$[M+H]^+$	209.0669	192.0404/166.0611/149.0343	MeOH
4	3-Methyl-quinoxaline-2-carboxylic acid	74003-63-7	$C_{10}H_8N_2O_2$	3.53	$[M+H]^+$	189.0659	145.0764/143.0605/77.0393	MeOH
5	Desacetyl cefotaxime	66340-28-1	$C_{14}H_{15}N_5O_6S_2$	2.31	$[M+H]^+$	414.0537	241.0391/126.0118/285.0112	ACN
6	4-Aminoantipyrine	83-07-8	$C_{11}H_{13}N_3O$	2.32	$[M+H]^+$	204.1131	56.0511/159.0911/187.0858	MeOH
7	4-Formylaminoantipyrine	1672-58-8	$C_{12}H_{13}N_3O_2$	2.86	$[M+H]^+$	232.1081	214.0977/204.1133/56.051	ACN
8	4-Isopropylaminoantipyrine	3615-24-5	$C_{14}H_{19}N_3O$	2.55	$[M+H]^+$	246.1601	56.0495/125.1064/111.0440	MeOH
9	5-Hydroxymebendazole	60254-95-7	$C_{16}H_{15}N_3O_3$	4.07	$[M+H]^+$	298.1186	266.0921/160.0504/220.087	DMSO
10	5-Hydroxythiabendazole	948-71-0	$C_{10}H_7N_3OS$	2.37	$[M+H]^+$	218.0383	191.0271/147.0548/192.0296	MeOH
11	17α-Estradiol	57-91-0	$C_{18}H_{24}O_2$	8.87	$[M+H]^+$	273.1849	145.0642/183.0804/223.0267	ACN
12	17α-Hydroxyprogesterone	68-96-2	$C_{21}H_{30}O_3$	13.97	$[M+H]^+$	331.2268	97.0174/109.0581/160.2	MeOH
13	(22R)-Budesonide	51372-29-3	$C_{25}H_{34}O_6$	9.82	$[M+H]^+$	431.2428	413.2329/323.1652/147.0801	MeOH
14	α-Trenbolone	80657-17-6	$C_{18}H_{22}O_2$	8.29	$[M+H]^+$	271.1693	253.1585/199.1123/197.0955	MeOH
15	N-Didesmethyl Sibutramine	84467-54-9	$C_{15}H_{22}ClN$	7.06	$[M+H]^+$	252.1514	125.1441/139.0312/153.0469	MeOH
16	Albendazole	54965-21-8	$C_{12}H_{15}N_3O_2S$	6.32	$[M+H]^+$	266.0958	234.0701/191.015/192.0227	ACN
17	Albendazole sulfoxide	75184-71-3	$C_{12}H_{15}N_3O_4S$	4.85	$[M+H]^+$	298.0856	159.0430/266.0596/224.0130	MeOH
18	Albendazolesulf oxide	54029-12-8	$C_{12}H_{15}N_3O_3S$	3.65	$[M+H]^+$	282.0907	208.0176/159.0428/191.0688	ACN/MeOH（1:1）
19	Albendazole-2-aminosulfone	80983-34-2	$C_{10}H_{13}N_3O_2S$	2.53	$[M+H]^+$	240.0801	133.0634/198.033/105.0453	MeOH
20	Aklomide	3011-89-0	$C_7H_5ClN_2O_3$	3.8	$[M+H]^+$	201.0062	137.9862/155.013/183.9795	MeOH
21	Alclomethasone dipropionate	66734-13-2	$C_{28}H_{37}ClO_7$	12.19	$[M+H]^+$	521.3228	171.0808/301.1578/275.1463	ACN
22	Alprazolam	28981-97-7	$C_{17}H_{13}ClN_4$	7.77	$[M+H]^+$	309.0902	281.0698/274.1199/205.0777	ACN
23	Atenolol	29122-68-7	$C_{14}H_{22}N_2O_3$	2.13	$[M+H]^+$	267.1703	145.0650/190.0967/56.0499	MeOH
24	Atropine	51-55-8	$C_{17}H_{23}NO_3$	3.09	$[M+H]^+$	290.1751	124.1116/93.07/91.0549	ACN
25	Estazolam	29975-16-4	$C_{16}H_{11}ClN_4$	7.38	$[M+H]^+$	295.0745	267.0566/205.0764/241.0526	MeOH
26	Methaqualone	72-44-6	$C_{16}H_{14}N_2O$	7.84	$[M+H]^+$	251.1179	132.0812/91.0553/117.0579	MeOH
27	Antipyrine	60-80-0	$C_{11}H_{12}N_2O$	3.72	$[M+H]^+$	189.1022	77.0396/56.0513/147.0913	MeOH
28	Amcinonide	51022-69-6	$C_{28}H_{35}FO_7$	12.07	$[M+H]^+$	503.244	339.159/321.1482/399.18	ACN
29	Aminophylline	317-34-0	$C_{16}H_{24}N_{10}O_4$	2.42	$[M+H]^+$	181.072	124.0502/69.0458/96.0557	ACN
30	Mebendazole amine	52329-60-9	$C_{14}H_{11}N_3O$	3.96	$[M+H]^+$	238.0975	77.0394/105.0342/133.0632	MeOH

Number	Compounds	CAS	Formula	RT（min）	Adduct	Parent ion（m/z）	Fragment ions（m/z）	Solvent
31	Amino tadalafil	385769-84-6	$C_{21}H_{18}N_4O_4$	7.19	$[M+H]^+$	391.1401	269.1043/169.0759/204.0808	MeOH
32	Tranexamic acid	1197-18-8	$C_8H_{15}NO_2$	4.85	$[M+H]^+$	158.1176	95.0855/67.0541/123.0803	ACN
33	Amlodipine	88150-42-9	$C_{20}H_{25}ClN_2O_5$	6.75	$[M+H]^+$	409.1525	238.0629/294.0894/377.1266	MeOH
34	Orbifloxacin	113617-63-3	$C_{19}H_{20}F_3N_3O_3$	3.54	$[M+H]^+$	396.153	295.1056/267.0378/352.1633	ACN
35	Oxfendazole	53716-50-0	$C_{15}H_{13}N_3O_3S$	4.95	$[M+H]^+$	316.075	159.043/191.0692/267.0462	ACN
36	Olaquindox	23696-28-8	$C_{12}H_{13}N_3O_4$	2.42	$[M+H]^+$	264.0979	143.0602/221.0556/212.0818	ACN
37	Oxazepam	604-75-1	$C_{15}H_{11}ClN_2O_2$	7.23	$[M+H]^+$	287.0582	241.0528/269.0478/104.0492	ACN
38	Oseltamivir	196618-13-0	$C_{16}H_{28}N_2O_4$	4.71	$[M+H]^+$	313.2122	166.0854/208.096/120.0448	MeOH
39	Ornidazole	16773-42-5	$C_7H_{10}ClN_3O_3$	3.76	$[M+H]^+$	220.0484	128.045/82.0523/111.0422	ACN
40	Baclofen	1134-47-0	$C_{10}H_{12}ClNO_2$	2.6	$[M+H]^+$	214.0629	77.0389/151.0309/116.0622	MeOH
41	Beclomethasone	4419-39-0	$C_{22}H_{29}ClO_5$	7.77	$[M+H]^+$	409.1776	391.1669/337.1797/279.1742	ACN
42	Beclomethasone dipropionate	5534-09-8	$C_{28}H_{37}ClO_7$	13.16	$[M+H]^+$	521.2301	503.2192/319.1695/393.206	ACN
43	Betamethasone	378-44-9	$C_{22}H_{29}FO_5$	7.41	$[M+H]^+$	393.2072	355.1906/337.1797/373.2008	ACN
44	Betamethasone dipropionate	5593-20-4	$C_{28}H_{37}FO_7$	12.57	$[M+H]^+$	505.2596	411.2168/319.1699/279.1746	ACN
45	Betamethasone 17-valerate	2152-44-5	$C_{27}H_{37}FO_6$	11.41	$[M+H]^+$	477.2647	355.1915/279.175/337.1809	ACN
46	Clobetasone butyrate	25122-57-0	$C_{26}H_{32}ClFO_5$	13.48	$[M+H]^+$	479.1995	343.1464/371.1414/279.1382	ACN
47	Nadrolone phenylpropionate	62-90-8	$C_{27}H_{34}O_3$	16.1	$[M+H]^+$	407.2581	105.07/257.19/133.0644	ACN
48	Benzimidazole	51-17-2	$C_7H_6N_2$	2.03	$[M+H]^+$	119.0604	65.0399/92.0497/59.0507	MeOH
49	Sulfabenzamide	127-71-9	$C_{13}H_{12}N_2O_3S$	5.6	$[M+H]^+$	277.0641	156.0108/108.0438/92.049	ACN
50	Febantel	58306-30-2	$C_{20}H_{22}N_4O_6S$	11.4	$[M+H]^+$	447.1333	383.082/280.0544/312.081	ACN
51	Azlocillin	37091-66-0	$C_{20}H_{23}N_5O_6S$	5.52	$[M+H]^+$	462.1442	218.0913/246.0867/175.0863	ACN/H_2O（1:3）
52	5-Nitrobenzimidazole	94-52-0	$C_7H_5N_3O_2$	3.04	$[M+H]^+$	164.0455	118.0529/91.0422/63.0243	ACN
53	Isoxsuprine	395-28-8	$C_{18}H_{23}NO_3$	4.32	$[M+H]^+$	302.1751	284.165/107.0492/133.0646	MeOH
54	Phenylethanolamine A	1346746-81-3	$C_{19}H_{24}N_2O_4$	5.96	$[M+H]^+$	345.1809	327.1708/150.0916/118.0654	MeOH
55	Phenformin	114-86-3	$C_{10}H_{15}N_5$	2.61	$[M+H]^+$	206.14	60.0572/105.0701/77.0395	MeOH
56	Oxacillin	66-79-5	$C_{19}H_{19}N_3O_5S$	7.9	$[M+H]^+$	402.1118	160.042/243.0757/144.0438	ACN
57	Pirlimycin	79548-73-5	$C_{17}H_{31}ClN_2O_5S$	4.14	$[M+H]^+$	411.1715	112.1127/363.1693/56.0514	MeOH
58	Piroxicam	36322-90-4	$C_{15}H_{13}N_3O_4S$	7.26	$[M+H]^+$	332.07	121.0395/95.0603/164.0818	ACN
59	Epitestosterone	481-30-1	$C_{19}H_{28}O_2$	9.98	$[M+H]^+$	289.2162	271.2055/109.0646/253.1951	ACN
60	Testosterone propionate	57-85-2	$C_{22}H_{32}O_3$	14.58	$[M+H]^+$	345.2424	271.2061/97.0657/109.0655	ACN
61	Nandrolone propionate	7207-92-3	$C_{21}H_{30}O_3$	10.08	$[M+H]^+$	331.2268	257.1911/239.1802/275.2018	MeOH
62	Oxibendazole	20559-55-1	$C_{12}H_{15}N_3O_3$	4.68	$[M+H]^+$	250.1186	176.0459/218.0929/148.0508	MeOH

Number	Compounds	CAS	Formula	RT（min）	Adduct	Parent ion（m/z）	Fragment ions（m/z）	Solvent
63	Budesonide	51333-22-3	$C_{25}H_{34}O_6$	9.97	$[M+H]^+$	431.2428	413.2329/323.1652/147.0801	MeOH
64	Halofuginone	55837-20-2	$C_{16}H_{17}BrClN_3O_3$	4.58	$[M+H]^+$	414.0215	100.0761/396.0102/120.0806	MeOH
65	Estradiol	50-28-2	$C_{18}H_{24}O_2$	9.19	$[M+H]^+$	273.1849	145.0642/183.0804/223.0267	MeOH
66	Estrone	53-16-7	$C_{18}H_{22}O_2$	9.63	$[M+H]^+$	271.1693	253.1596/199.1115/165.0700	ACN
67	N-Acetylsulfamethoxazole	21312-10-7	$C_{12}H_{13}N_3O_4S$	5.12	$[M+H]^+$	296.07	198.022/134.0601/65.0403	ACN
68	Aceclofenac	89796-99-6	$C_{16}H_{13}Cl_2NO_4$	10.92	$[M+H]^+$	354.0294	214.0419/250.0186/215.0498	MeOH
69	Betamethasone 21-acetate	987-24-6	$C_{24}H_{31}FO_6$	9.29	$[M+H]^+$	435.2177	279.1786/147.0803/337.1834	ACN
70	Dexamethasone 21-acetate	1177-87-3	$C_{24}H_{31}FO_6$	9.56	$[M+H]^+$	435.2177	147.0802/237.1271/291.1745	ACN
71	Fluorometholone 17-Acetate	3801-06-7	$C_{24}H_{31}FO_5$	9.96	$[M+H]^+$	419.2228	279.1753/321.1861/339.1969	ACN
72	Fludrocortisone 21-acetate	514-36-3	$C_{23}H_{31}FO_6$	8.54	$[M+H]^+$	423.2177	239.1425/325.179/343.1893	ACN
73	Flugestone acetate	2529-45-5	$C_{23}H_{31}FO_5$	9.43	$[M+H]^+$	407.2228	267.1747/225.1639/309.186	ACN
74	Cyproterone acetate	427-51-0	$C_{24}H_{29}ClO_4$	12.11	$[M+H]^+$	417.1827	357.1618/279.173/321.1852	MeOH
75	Medroxyprogesterone 17-acetate	71-58-9	$C_{24}H_{34}O_4$	12.61	$[M+H]^+$	387.253	199.1484/173.1329/143.0858	ACN
76	Cortisone 21-acetate	50-04-4	$C_{23}H_{30}O_6$	8.87	$[M+H]^+$	403.2115	343.1906/163.1116/361.2006	ACN
77	Chlormadinone acetate	302-22-7	$C_{23}H_{29}ClO_4$	12.43	$[M+H]^+$	405.1827	301.1354/309.1849/267.1739	ACN
78	Melengestrol acetate	2919-66-6	$C_{25}H_{32}O_4$	12.59	$[M+H]^+$	397.2373	337.2167/279.1748/236.1558	ACN
79	Prednisone 21-acetate	125-10-0	$C_{23}H_{28}O_6$	8.73	$[M+H]^+$	401.1959	295.169/313.1795/341.1746	ACN
80	Prednisolone 21-acetate	52-21-1	$C_{23}H_{30}O_6$	8.38	$[M+H]^+$	403.2115	385.2015/307.1695/289.1589	ACN
81	17α-Hydroxyprogesterone acetate	302-23-8	$C_{23}H_{32}O_4$	11.69	$[M+H]^+$	373.2376	313.2166/109.0650/271.2057	ACN
82	Hydrocortisone acetate	50-03-3	$C_{23}H_{32}O_6$	8.5	$[M+H]^+$	405.2272	309.1853/327.1963/241.1586	ACN
83	Triamcinolone acetonide acetate	3870-07-3	$C_{26}H_{33}FO_7$	10.68	$[M+H]^+$	477.2283	339.1599/457.222/439.2119	ACN
84	Triamcinolone diacetate	67-78-7	$C_{25}H_{31}FO_8$	8.31	$[M+H]^+$	479.2076	441.1905/321.1486/399.1798	ACN
85	Norethisterone acetate	51-98-9	$C_{22}H_{28}O_3$	12.34	$[M+H]^+$	341.2111	281.1899/109.0645/145.1009	MeOH
86	Danofloxacin	112398-08-0	$C_{19}H_{20}FN_3O_3$	3.34	$[M+H]^+$	358.1562	340.1445/255.0567/82.0667	ACN
87	Azaperone	1649-18-9	$C_{19}H_{22}FN_3O$	3.48	$[M+H]^+$	328.182	165.0708/121.0759/123.0237	ACN
88	Deflazacort	14484-47-0	$C_{25}H_{31}NO_6$	8.75	$[M+H]^+$	442.2224	400.2115/424.2121/142.0496	ACN
89	Dexamethasone	50-02-2	$C_{22}H_{29}FO_5$	7.32	$[M+H]^+$	393.2072	237.1257/147.0789/355.1878	ACN
90	Diazepam	439-14-5	$C_{16}H_{13}ClN_2O$	9.06	$[M+H]^+$	285.0789	193.0884/154.0414/222.1149	ACN
91	Dicyclanil	112636-83-6	$C_8H_{10}N_6$	2.09	$[M+H]^+$	191.104	150.0641/163.0724/151.0721	MeOH
92	Buquinolate	5486-03-3	$C_{20}H_{27}NO_5$	10.66	$[M+H]^+$	362.1962	204.0287/316.1531/260.0918	乙醇
93	Sultamicillin tosilate	83105-70-8	$C_{32}H_{38}N_4O_{12}S_3$	5.55	$[M+H]^+$	595.1527	160.042/114.0365/106.0648	ACN
94	Acetaminophen	103-90-2	$C_8H_9NO_2$	2.42	$[M+H]^+$	152.0706	110.0598/93.0333/65.0383	ACN
95	Doramectin	117704-25-3	$C_{50}H_{74}O_{14}$	16.79	$[M+H]^+$	899.5151	593.348/145.0861/219.1747	MeOH
96	Doxycycline	564-25-0	$C_{22}H_{24}N_2O_8$	4.63	$[M+H]^+$	445.1605	410.122/427.1485/154.0503	ACN

Number	Compounds	CAS	Formula	RT（min）	Adduct	Parent ion（m/z）	Fragment ions（m/z）	Solvent
97	Oxolinic acid	14698-29-4	$C_{13}H_{11}NO_5$	5.45	$[M+H]^+$	262.071	244.0604/216.0287/160.0389	ACN
98	Enrofloxacin	93106-60-6	$C_{19}H_{22}FN_3O_3$	3.42	$[M+H]^+$	360.1718	342.1613/286.0977/316.1822	ACN
99	Diflorasone diacetate	33564-31-7	$C_{26}H_{32}F_2O_7$	10.73	$[M+H]^+$	495.2189	317.154/335.1649/395.186	ACN
100	Dimetridazole	551-92-8	$C_5H_7N_3O_2$	2.7	$[M+H]^+$	142.0611	96.0682/95.0604/81.0447	ACN
101	Dinitolmide	148-01-6	$C_8H_7N_3O_5$	4.05	$[M+H]^+$	226.0459	139.0136/122.0108/67.0426	ACN
102	Dioxopromethazine	13754-56-8	$C_{17}H_{20}N_2O_2S$	4.23	$[M+H]^+$	317.1318	86.0963/167.0725/272.0742	MeOH
103	Felodipine	72509-76-3	$C_{18}H_{19}Cl_2NO_4$	12.21	$[M+H]^+$	384.0764	338.0337/352.0496/324.0181	ACN
104	Phenacetin	62-44-2	$C_{10}H_{13}NO_2$	5.18	$[M+H]^+$	180.1019	138.0913/110.0602/65.04	ACN
105	Fenbendazole	43210-67-9	$C_{15}H_{13}N_3O_2S$	8.09	$[M+H]^+$	300.0801	268.0545/159.0431/131.0476	ACN
106	Fenbendazole sulfone	54029-20-8	$C_{15}H_{13}N_3O_4S$	6.43	$[M+H]^+$	332.07	300.0441/159.0428/131.0479	MeOH
107	Fenfluramine	458-24-2	$C_{12}H_{16}F_3N$	4.86	$[M+H]^+$	232.1308	159.0407/46.0648/109.0439	MeOH
108	Phenolphthalein	77-09-8	$C_{20}H_{14}O_4$	7.62	$[M+H]^+$	319.0965	86.0974/58.0673/225.0555	MeOH
109	Rimsulfuron	122931-48-0	$C_{14}H_{17}N_5O_7S_2$	7.77	$[M+H]^+$	432.0642	182.0558/139.0498/325.0955	ACN
110	Furaltadone	139-91-3	$C_{13}H_{16}N_4O_6$	2.46	$[M+H]^+$	325.1143	237.0399/217.034/202.0714	ACN
111	2-NP-AMOZ	183193-59-1	$C_{15}H_{18}N_4O_5$	3.03	$[M+H]^+$	335.135	128.1063/100.0756/291.1457	MeOH
112	Nitrofurantoin	67-20-9	$C_8H_6N_4O_5$	3.55	$[M+H]^+$	239.0411	122.0107/221.0917/67.0427	ACN
113	2-NP-AHD	623145-57-3	$C_{10}H_8N_4O_4$	4.79	$[M+H]^+$	249.0618	134.0237/178.0611/104.0259	MeOH
114	Furazolidone	67-45-8	$C_8H_7N_3O_5$	4.03	$[M+H]^+$	226.0459	122.0106/139.0134/67.0414	ACN
115	2-NP-AOZ	19687-73-1	$C_{10}H_9N_3O_4$	5.51	$[M+H]^+$	236.0666	134.0239/104.0254/78.047	MeOH
116	Flubendazole	31430-15-6	$C_{16}H_{12}FN_3O_3$	7.03	$[M+H]^+$	314.0936	282.0678/123.0252/95.0293	ACN
117	Flufenamic acid	530-78-9	$C_{14}H_{10}F_3NO_2$	11.89	$[M+H]^+$	282.0736	264.0625/167.0728/244.0575	ACN
118	Flumequin	42835-25-6	$C_{14}H_{12}FNO_3$	7.12	$[M+H]^+$	262.0874	244.0768/202.0301/126.0337	ACN
119	Fleroxacin	79660-72-3	$C_{17}H_{18}F_3N_3O_3$	3.06	$[M+H]^+$	370.1373	269.0884/352.125/326.1459	ACN
120	Flucloxacillin	5250-39-5	$C_{19}H_{17}ClFN_3O_5S$	8.91	$[M+H]^+$	454.0634	295.0269/160.0416/114.0372	MeOH
121	Fluorometholone	426-13-1	$C_{22}H_{29}FO_4$	8.44	$[M+H]^+$	377.2123	279.1748/321.1856/339.1964	ACN
122	Flumethasone	2135-17-3	$C_{22}H_{28}F_2O_5$	7.36	$[M+H]^+$	411.1978	253.1215/121.0648/235.1101	ACN
123	Flunixin	38677-85-9	$C_{14}H_{11}F_3N_2O_2$	8.48	$[M+H]^+$	297.0845	279.0749/264.0517/259.0683	ACN
124	Haloperidol	52-86-8	$C_{21}H_{23}ClFNO_2$	6.27	$[M+H]^+$	376.1474	165.0697/123.0241/358.1368	MeOH
125	Fluprednisolone	53-34-9	$C_{21}H_{27}FO_5$	6.18	$[M+H]^+$	379.1915	323.1645/171.0803/341.1752	ACN
126	Fluocinolone acetonide	67-73-2	$C_{24}H_{30}F_2O_6$	8.19	$[M+H]^+$	453.2083	413.1947/337.1425/433.2007	ACN
127	Fluocinonide	356-12-7	$C_{26}H_{32}F_2O_7$	10.73	$[M+H]^+$	495.2189	317.154/335.1649/395.186	ACN
128	Fludroxycortide	1524-88-5	$C_{24}H_{33}FO_6$	8.16	$[M+H]^+$	437.2334	361.1803/341.1742/323.1629	ACN
129	Fluticasone propionate	80474-14-2	$C_{25}H_{31}F_3O_5S$	12.26	$[M+H]^+$	501.1917	293.1548/313.161/275.1428	ACN
130	Formoterol	73573-87-2	$C_{19}H_{24}N_2O_4$	3.67	$[M+H]^+$	345.1809	121.0646/149.0957/327.1698	MeOH
131	Testosterone	58-22-0	$C_{19}H_{28}O_2$	9.36	$[M+H]^+$	289.2162	109.0653/97.0652/123.0801	ACN
132	Glibenclamide	10238-21-8	$C_{23}H_{28}ClN_3O_5S$	11.18	$[M+H]^+$	494.1511	369.0664/169.005/304.0745	ACN

Number	Compounds	CAS	Formula	RT（min）	Adduct	Parent ion（m/z）	Fragment ions（m/z）	Solvent
133	Glipizide	29094-61-9	$C_{21}H_{27}N_5O_4S$	8.46	$[M+H]^+$	446.1857	321.101/286.064/347.0803	ACN
134	Glibornuride	26944-48-9	$C_{18}H_{26}N_2O_4S$	10.21	$[M+H]^+$	367.1686	170.1538/152.1433/135.1167	MeOH
135	Gliquidone	33342-05-1	$C_{27}H_{33}N_3O_6S$	12.66	$[M+H]^+$	528.2163	403.1312/386.1045/167.0155	MeOH
136	Glimepiride	93479-97-1	$C_{24}H_{34}N_4O_5S$	11.52	$[M+H]^+$	491.2323	126.0918/352.133/225.0337	ACN
137	Gliclazide	21187-98-4	$C_{15}H_{21}N_3O_3S$	9.44	$[M+H]^+$	324.1376	127.1225/110.096/153.1017	MeOH
138	Guanfacine	29110-47-2	$C_9H_9Cl_2N_3O$	3.84	$[M+H]^+$	246.0195	158.9756/60.0575/123.0003	ACN
139	Decoquinate	18507-89-6	$C_{24}H_{35}NO_5$	14.2	$[M+H]^+$	418.2588	372.215/204.0294/232.0596	乙醇
140	Halcinonide	3093-35-4	$C_{24}H_{32}ClFO_5$	11.71	$[M+H]^+$	455.1995	377.1516/359.1398/227.142	ACN
141	Erythromycin	114-07-8	$C_{37}H_{67}NO_{13}$	6.13	$[M+H]^+$	734.4685	158.1176/576.3746/267.1603	ACN/H_2O（1:3）
142	Ciprofloxacin	85721-33-1	$C_{17}H_{18}FN_3O_3$	3.16	$[M+H]^+$	332.1405	314.1301/288.1509/231.0562	MeOH
143	Sulfaphenazole	526-08-9	$C_{15}H_{14}N_4O_2S$	6.07	$[M+H]^+$	315.091	158.0716/160.0871/159.0792	ACN
144	Sulfapyridine	144-83-2	$C_{11}H_{11}N_3O_2S$	2.99	$[M+H]^+$	250.0645	156.0113/108.0441/184.0869	ACN
145	Sulfapyrazole	852-19-7	$C_{16}H_{16}N_4O_2S$	6.53	$[M+H]^+$	329.1067	172.0871/145.0757/173.0949	MeOH
146	Sulfacetamide	144-80-9	$C_8H_{10}N_2O_3S$	2.54	$[M+H]^+$	215.0485	156.011/92.0495/65.0399	ACN
147	Sulfameter	651-06-9	$C_{11}H_{12}N_4O_3S$	3.81	$[M+H]^+$	281.0703	156.0121/126.067/92.0517	ACN
148	Sulfamoxole	729-99-7	$C_{11}H_{13}N_3O_3S$	3.4	$[M+H]^+$	268.075	156.011/108.0439/113.0707	MeOH
149	Sulfamethazine	57-68-1	$C_{12}H_{14}N_4O_2S$	3.62	$[M+H]^+$	279.091	186.0338/124.0868/108.0443	ACN
150	Sulfisoxazole	127-69-5	$C_{11}H_{13}N_3O_3S$	5.06	$[M+H]^+$	268.075	156.0116/113.0714/92.05	ACN
151	Sulfisomidine	515-64-0	$C_{12}H_{14}N_4O_2S$	2.44	$[M+H]^+$	279.091	124.0865/186.0338/108.0449	MeOH
152	Sulfamerazine	127-79-7	$C_{11}H_{12}N_4O_2S$	3.22	$[M+H]^+$	265.0754	156.0112/172.0174/108.044	ACN
153	Sulfamethoxazole	723-46-6	$C_{10}H_{11}N_3O_3S$	4.7	$[M+H]^+$	254.0594	156.0115/108.0444/92.0495	ACN
154	Sulfamethizole	144-82-1	$C_9H_{10}N_4O_2S_2$	3.62	$[M+H]^+$	271.0318	156.0112/108.0441/92.0492	ACN
155	Sulfadimethoxine	122-11-2	$C_{12}H_{14}N_4O_4S$	5.84	$[M+H]^+$	311.0809	108.0443/92.0494/156.0111	ACN
156	Sulfamonomethoxine	1220-83-3	$C_{11}H_{12}N_4O_3S$	3.62	$[M+H]^+$	281.0703	156.0117/92.0497/108.0441	ACN
157	Sulfaquinoxaline	59-40-5	$C_{14}H_{12}N_4O_2S$	5.93	$[M+H]^+$	301.0754	156.011/108.0439/92.049	ACN
158	Sulfadoxine	2447-57-6	$C_{12}H_{14}N_4O_4S$	4.71	$[M+H]^+$	311.0809	156.0117/108.0443/92.0494	ACN
159	Sulfachloropyridazine	80-32-0	$C_{10}H_9ClN_4O_2S$	4.35	$[M+H]^+$	285.0208	92.0644/108.0276/127.9599	ACN
160	Sulfadiazine	68-35-9	$C_{10}H_{10}N_4O_2S$	2.81	$[M+H]^+$	251.0597	156.0108/108.0439/92.049	ACN
161	Sulfathiazole	72-14-0	$C_9H_9N_3O_2S_2$	2.9	$[M+H]^+$	256.0209	156.011/108.044/92.0491	ACN
162	Sulfanitran	122-16-7	$C_{14}H_{13}N_3O_5S$	7.48	$[M+H]^+$	336.0649	136.0299/134.0592/137.0375	MeOH
163	Gemifioxacin	175463-14-6	$C_{18}H_{20}FN_5O_4$	4.28	$[M+H]^+$	390.1572	372.1469/313.1335/328.1203	MeOH
164	Kitasamycin	1392-21-8	$C_{40}H_{67}NO_{14}$	7.82	$[M+H]^+$	786.4634	174.1125/109.0648/558.3265	ACN/H_2O（1:3）
165	Hydroxyprogesterone caproate	630-56-8	$C_{27}H_{40}O_4$	15.42	$[M+H]^+$	429.2999	313.216/271.2057/295.2057	ACN

Number	Compounds	CAS	Formula	RT（min）	Adduct	Parent ion（m/z）	Fragment ions（m/z）	Solvent
166	Gatifloxacin	112811-59-3	$C_{19}H_{22}FN_3O_4$	3.73	$[M+H]^+$	376.1667	332.1768/358.1556/261.1025	MeOH
167	Tolbutamide	64-77-7	$C_{12}H_{18}N_2O_3S$	8.22	$[M+H]^+$	271.1111	155.0154/91.0537/74.096	ACN
168	Tosufloxacin	100490-36-6	$C_{19}H_{15}F_3N_4O_3$	4.34	$[M+H]^+$	405.1169	387.1066/344.1007/388.0914	ACN
169	Mebendazole	31431-39-7	$C_{16}H_{13}N_3O_3$	6.56	$[M+H]^+$	296.103	264.0764/105.0339/77.0399	ACN
170	Xylazine	7361-61-7	$C_{12}H_{16}N_2S$	3.61	$[M+H]^+$	221.1107	121.0279/164.052/91.0543	MeOH
171	Megestrol	3562-63-8	$C_{22}H_{30}O_3$	10.7	$[M+H]^+$	343.2268	325.2161/267.1742/224.156	ACN
172	Mefenamic acid	61-68-7	$C_{15}H_{15}NO_2$	11.84	$[M+H]^+$	242.1176	224.1075/209.0842/180.0817	ACN
173	17-Methyltestosterone	58-18-4	$C_{20}H_{30}O_2$	10.01	$[M+H]^+$	303.2319	285.2218/109.0643/97.0646	ACN
174	Methylprednisolone	83-43-2	$C_{22}H_{30}O_5$	7.16	$[M+H]^+$	375.2166	357.205/161.0952/185.0953	ACN
175	Methylprednisolone acetate	53-36-1	$C_{24}H_{32}O_6$	9.29	$[M+H]^+$	417.2272	399.2179/321.1859/339.1965	ACN
176	D-(-)-Norgestrel	797-63-7	$C_{21}H_{28}O_2$	10.65	$[M+H]^+$	313.2162	245.1903/109.0647/295.2058	ACN
177	Meclocycline	2013-58-3	$C_{22}H_{21}ClN_2O_8$	5.41	$[M+H]^+$	477.1059	460.0782/235.0152/226.071	ACN
178	Methacycline	914-00-1	$C_{22}H_{22}N_2O_8$	4.47	$[M+H]^+$	443.1449	426.1168/201.0544/381.0604	ACN
179	Metronidazole	443-48-1	$C_6H_9N_3O_3$	2.36	$[M+H]^+$	172.0717	128.0449/82.0521/111.0422	ACN
180	Trimethoprim	738-70-5	$C_{14}H_{18}N_4O_3$	2.88	$[M+H]^+$	291.1452	230.116/261.0987/123.0662	ACN
181	Nequinate	13997-19-8	$C_{22}H_{23}NO_4$	10.69	$[M+H]^+$	366.17	334.1431/201.0421/91.055	DMF
182	Josamycin	16846-24-5	$C_{42}H_{69}NO_{15}$	8.19	$[M+H]^+$	828.474	174.1135/229.1446/600.3403	ACN/H_2O（1:3）
183	Crystal violet	548-62-9	$C_{25}H_{30}ClN_3$	10.23	$[M]^+$	372.2434	356.2102/340.1797/251.1537	ACN
184	Amantadine	768-94-5	$C_{10}H_{17}N$	2.88	$[M+H]^+$	152.1434	135.1167/79.055/93.0703	ACN
185	Rimantadine	13392-28-4	$C_{12}H_{21}N$	4.42	$[M+H]^+$	180.1747	163.1483/77.0391/79.0546	MeOH
186	Chlortetracycline	57-62-5	$C_{22}H_{23}ClN_2O_8$	4.31	$[M+H]^+$	479.1216	462.0943/196.9995/197.0004	ACN
187	Metoprolol tartrate	56392-17-7	$C_{15}H_{25}NO_3$	3.62	$[M+H]^+$	268.1907	191.1063/116.1067/133.0644	MeOH
188	Carazolol	57775-29-8	$C_{18}H_{22}N_2O_2$	4.75	$[M+H]^+$	299.1754	116.1078/222.0907/194.0956	MeOH
189	Caffeine	58-08-2	$C_8H_{10}N_4O_2$	2.87	$[M+H]^+$	195.0877	138.0655/110.0706/42.0336	ACN
190	Carbadox	6804-07-5	$C_{11}H_{10}N_4O_4$	3.2	$[M+H]^+$	263.0775	132.0676/168.0441/203.0122	MeOH
191	Carprofen	53716-49-7	$C_{15}H_{12}ClNO_2$	10.29	$[M+H]^+$	274.0629	228.0565/193.0885/192.0803	ACN
192	Captopril	62571-86-2	$C_9H_{15}NO_3S$	3.64	$[M+H]^+$	218.0845	70.0657/75.0266/116.0703	MeOH
193	Cambendazole	26097-80-3	$C_{14}H_{14}N_4O_2S$	4.55	$[M+H]^+$	303.091	217.0539/261.0448/190.0435	MeOH
194	Cortisone	53-06-5	$C_{21}H_{28}O_5$	6.45	$[M+H]^+$	361.201	163.1119/121.0649/145.1011	ACN
195	Clarithromycin	81103-11-9	$C_{38}H_{69}NO_{13}$	7.39	$[M+H]^+$	748.4842	158.1174/590.3896/558.3633	MeOH
196	Clonidine	4205-90-7	$C_9H_9Cl_2N_3$	2.48	$[M+H]^+$	230.0246	212.9976/159.9718/132.9607	MeOH
197	Clindamycin	18323-44-9	$C_{18}H_{33}ClN_2O_5S$	4.47	$[M+H]^+$	425.1872	126.1278/377.1845/389.2111	ACN/H_2O（1:3）
198	Clinafloxacin	105956-97-6	$C_{17}H_{17}ClFN_3O_3$	3.78	$[M+H]^+$	366.1015	305.086/349.0762/348.09	MeOH
199	Clenhexerol	38339-23-0	$C_{14}H_{22}Cl_2N_2O$	5.38	$[M+H]^+$	305.1182	203.0131/132.0684/168.045	MeOH

Number	Compounds	CAS	Formula	RT（min）	Adduct	Parent ion（m/z）	Fragment ions（m/z）	Solvent
200	Clencyclohexerol	157877-79-7	$C_{14}H_{20}Cl_2N_2O_2$	2.75	$[M+H]^+$	319.0975	86.0974/58.0673/225.0555	MeOH
201	Clenbuterol	37148-27-9	$C_{12}H_{18}Cl_2N_2O$	3.64	$[M+H]^+$	277.0869	203.0137/168.0449/132.0683	ACN
202	Malachite green oxalate	2437-29-8	$C_{52}H_{54}N_4O_{12}$	8.74	$[M]^+$	329.2018	313.1699/208.1119/98.9848	ACN
203	Quinoxaline-2-carboxylic acid	879-65-2	$C_9H_6N_2O_2$	3.34	$[M+H]^+$	175.0502	129.045/131.0607/102.0341	MeOH
204	Labetalol	36894-69-6	$C_{19}H_{24}N_2O_3$	4.68	$[M+H]^+$	329.186	311.1741/162.0553/294.1478	MeOH
205	Ractopamine	97825-25-7	$C_{18}H_{23}NO_3$	3.21	$[M+H]^+$	302.1751	284.165/107.0492/133.0646	ACN
206	Lorazepam	846-49-1	$C_{15}H_{10}Cl_2N_2O_2$	7.57	$[M+H]^+$	321.0192	275.0144/229.1349/303.0092	ACN
207	Rilmenidine	54187-04-1	$C_{10}H_{16}N_2O$	3.08	$[M+H]^+$	181.1335	95.0862/67.0554/55.0559	MeOH
208	Chlordiazepoxide	58-25-3	$C_{16}H_{14}ClN_3O$	4.51	$[M+H]^+$	300.0898	227.0499/282.0789/283.0867	MeOH
209	Ritodrine	26652-09-5	$C_{17}H_{21}NO_3$	2.57	$[M+H]^+$	288.1594	270.1501/121.0644/150.091	MeOH
210	Reserpine	50-55-5	$C_{33}H_{40}N_2O_9$	7.75	$[M+H]^+$	609.2807	195.0652/397.2121/174.0915	MeOH
211	Lincomycin	154-21-2	$C_{18}H_{34}N_2O_6S$	2.25	$[M+H]^+$	407.221	126.1271/359.2174/389.2103	ACN/H_2O（1：3）
212	Rosiglitazone	122320-73-4	$C_{18}H_{19}N_3O_3S$	4.13	$[M+H]^+$	358.122	135.0912/119.0604/78.0344	MeOH
213	Roxithromycin	80214-83-1	$C_{41}H_{76}N_2O_{15}$	7.58	$[M+H]^+$	837.5319	679.4383/158.1182/116.108	ACN/H_2O（1:3）
214	Tetrahydropalmatine	2934-97-6	$C_{21}H_{25}NO_4$	4.8	$[M+H]^+$	356.1856	192.1002/165.0898/176.0694	ACN
215	Ronidazole	7681-76-7	$C_6H_8N_4O_4$	2.6	$[M+H]^+$	201.0618	140.0457/55.0457/54.038	ACN
216	Spiramycin	8025-81-8	$C_{43}H_{74}N_2O_{14}$	4.22	$[M+H]^+$	843.5213	174.1121/142.1225/540.3181	MeOH
217	Lovastatin	75330-75-5	$C_{24}H_{36}O_5$	12.98	$[M+H]^+$	405.2636	199.148/225.1637/285.185	ACN
218	Lovastatin sodium salt	75225-50-2	$C_{24}H_{37}NaO_6$	11.45	$[M+H]^+$	423.2741	199.1471/173.1318/225.1628	MeOH
219	Roxarsone	121-19-7	$C_6H_6AsNO_6$	2.22	$[M+H]^+$	263.9484	90.916/92.0258/245.9365	MeOH
220	Lomefloxacin	98079-51-7	$C_{17}H_{19}F_2N_3O_3$	3.33	$[M+H]^+$	352.1467	265.1156/237.0842/334.1355	ACN
221	Clobetasol 17-propionate	25122-46-7	$C_{25}H_{32}ClFO_5$	12.14	$[M+H]^+$	467.1995	355.1468/373.1575/263.1431	ACN
222	Chlorpheniramine maleate	113-92-8	$C_{20}H_{23}ClN_2O_4$	4.21	$[M+H]^+$	275.131	230.0731/167.0728/201.0335	MeOH
223	Clorprenaline	3811-25-4	$C_{11}H_{16}ClNO$	3.06	$[M+H]^+$	214.0993	154.0428/118.0666/119.0743	ACN
224	Chlorpromazine	50-53-3	$C_{17}H_{19}ClN_2S$	7.41	$[M+H]^+$	319.103	86.0974/58.0671/225.0555	MeOH
225	Chlormadinone	1961-77-9	$C_{21}H_{27}ClO_3$	10.87	$[M+H]^+$	363.1722	309.1858/345.1625/267.1735	ACN
226	5-Chloro-1-methyl-4-nitroimidazole	4897-25-0	$C_4H_4ClN_3O_2$	3.19	$[M+H]^+$	162.0065	145.0031/116.0134/132.0076	ACN
227	Chlormezanone	80-77-3	$C_{11}H_{12}ClNO_3S$	6.08	$[M+H]^+$	274.0299	154.0411/209.0595/152.0373	MeOH
228	Clopidol	2971-90-6	$C_7H_7Cl_2NO$	2.72	$[M+H]^+$	191.9978	101.0157/87.0006/86.9997	MeOH
229	Closantel	57808-65-8	$C_{22}H_{14}Cl_2I_2N_2O_2$	17.13	$[M+H]^+$	662.8595	264.0333/635.8464/372.8194	MeOH
230	Clonazepam	1622-61-3	$C_{15}H_{10}ClN_3O_3$	7.72	$[M+H]^+$	316.0484	270.0557/241.0528/214.0419	ACN

Number	Compounds	CAS	Formula	RT（min）	Adduct	Parent ion（m/z）	Fragment ions（m/z）	Solvent
231	Cloxacillin	61-72-3	$C_{19}H_{18}ClN_3O_5S$	8.57	$[M+H]^+$	436.0729	277.0356/160.0413/178.0051	ACN
232	Marbofloxacin	115550-35-1	$C_{17}H_{19}FN_4O_4$	2.92	$[M+H]^+$	363.1463	345.136/72.0833/320.1042	MeOH
233	Maduramycin ammonium	84878-61-5	$C_{47}H_{80}O_{17}$	6.77	$[M+H]^+$	916.539	174.1133/772.4491/145.0858	MeOH
234	Mapenterol	54238-51-6	$C_{14}H_{20}ClF_3N_2O$	5.18	$[M+H]^+$	325.1289	237.0399/217.034/202.0714	MeOH
235	Mevastatin	73573-88-3	$C_{23}H_{34}O_5$	12.23	$[M+H]^+$	391.2479	185.1316/159.1159/211.1476	ACN
236	Mesterolone	1424-00-6	$C_{20}H_{32}O_2$	11.05	$[M+H]^+$	305.2475	269.2265/287.237/229.1948	ACN
237	Melengestrol	5633-18-1	$C_{23}H_{30}O_3$	10.58	$[M+H]^+$	355.2268	337.2195/279.1745/221.1328	ACN
238	Meloxicam	71125-38-7	$C_{14}H_{13}N_3O_4S_2$	9.12	$[M+H]^+$	352.042	115.032/141.0112/184.0535	ACN
239	Medetomidine	86347-14-0	$C_{13}H_{16}N_2$	4.56	$[M+H]^+$	201.1386	95.0611/68.051/41.0418	MeOH
240	Mestanolone	521-11-9	$C_{20}H_{32}O_2$	11.2	$[M+H]^+$	305.2475	269.2265/287.237/229.1948	ACN
241	Methandrostenolone	72-63-9	$C_{20}H_{28}O_2$	9.22	$[M+H]^+$	301.2162	121.0646/149.1323/283.2055	MeOH
242	Midazolam	59467-70-8	$C_{18}H_{13}ClFN_3$	5.52	$[M+H]^+$	326.0855	291.1162/249.0819/209.0631	ACN
243	Minocycline	10118-90-8	$C_{23}H_{27}N_3O_7$	2.68	$[M+H]^+$	458.1922	441.1648/352.1192/337.0959	ACN
244	Meprobamate	57-53-4	$C_9H_{18}N_2O_4$	4.87	$[M+H]^+$	219.1339	55.0542/97.101/158.1175	MeOH
245	Muraglitazar	331741-94-7	$C_{29}H_{28}N_2O_7$	12.68	$[M+H]^+$	517.1969	186.0907/292.1333/144.08	MeOH
246	Mometasone	105102-22-5	$C_{22}H_{28}Cl_2O_4$	10.59	$[M+H]^+$	521.1492	503.1397/355.1468/263.1428	ACN
247	Moxidectin	113507-06-5	$C_{37}H_{53}NO_8$	17.31	$[M+H]^+$	640.3844	498.2867/528.2937/416.26	MeOH
248	Moxifloxacin	51096-09-2	$C_{21}H_{24}FN_3O_4$	4.33	$[M+H]^+$	402.1824	384.1707/358.1911/364.1643	MeOH
249	Nadifloxacin	124858-35-1	$C_{19}H_{21}FN_2O_4$	7.06	$[M+H]^+$	361.1558	343.1453/283.0881/257.0722	MeOH
250	Nabumetone	42924-53-8	$C_{15}H_{16}O_2$	10.48	$[M+H]^+$	229.1223	171.0802/128.0616/156.0565	ACN
251	Nalidixic acid	389-08-2	$C_{12}H_{12}N_2O_3$	6.77	$[M+H]^+$	233.0921	215.0814/187.0499/159.0548	ACN
252	Nafcillin	147-52-4	$C_{21}H_{22}N_2O_5S$	8.93	$[M+H]^+$	415.1322	199.0744/171.0436/256.0963	ACN
253	Naproxen	22204-53-1	$C_{14}H_{14}O_3$	5.07	$[M+H]^+$	231.1016	199.0616/143.0599/185.0951	MeOH
254	Nimodipine	66085-59-4	$C_{21}H_{26}N_2O_7$	11.47	$[M+H]^+$	419.1813	343.128/301.0812/359.1231	MeOH
255	Nitrendipine	39562-70-4	$C_{18}H_{20}N_2O_6$	10.86	$[M+H]^+$	361.1394	315.0972/329.1129/269.1039	ACN
256	Norfloxacin	70458-96-7	$C_{16}H_{18}FN_3O_3$	3.04	$[M+H]^+$	320.1405	302.1301/276.151/233.1085	ACN
257	Nortestosterone	434-22-0	$C_{18}H_{26}O_2$	8.69	$[M+H]^+$	275.2006	257.1902/239.1795/109.0649	ACN
258	Piperacillin	61477-96-1	$C_{23}H_{27}N_5O_7S$	6.3	$[M+H]^+$	518.1704	143.0806/160.0426/115.0503	ACN/H_2O（1:9）
259	Prazosin	19216-56-9	$C_{19}H_{21}N_5O_4$	4.25	$[M+H]^+$	384.1666	247.1193/231.0875/95.0131	MeOH
260	Pefloxacin	70458-92-3	$C_{17}H_{20}FN_3O_3$	3.13	$[M+H]^+$	334.1562	316.1439/233.1073/290.165	ACN
261	Penbutolol	36507-48-9	$C_{18}H_{29}NO_2$	7.16	$[M+H]^+$	292.2271	236.1639/201.1275/133.0654	ACN
262	Corticosterone	50-22-6	$C_{21}H_{30}O_4$	7.75	$[M+H]^+$	347.2217	329.2112/121.0646/311.2007	ACN
263	Prednicarbate	73771-04-7	$C_{27}H_{36}O_8$	11.99	$[M+H]^+$	489.2483	381.2065/471.2381/307.17	ACN
264	Prednisone	53-03-2	$C_{21}H_{26}O_5$	6.29	$[M+H]^+$	359.1853	341.1734/313.179/147.0795	ACN
265	Prednisolone	50-24-8	$C_{21}H_{28}O_5$	6.27	$[M+H]^+$	361.201	343.1885/147.0804/307.167	ACN

Number	Compounds	CAS	Formula	RT（min）	Adduct	Parent ion（m/z）	Fragment ions（m/z）	Solvent
266	Propranolol	525-66-6	$C_{16}H_{21}NO_2$	5.12	$[M+H]^+$	260.1645	183.0809/116.1074/157.065	ACN
267	Hydroxy metronidazole	4812-40-2	$C_6H_9N_3O_4$	2.16	$[M+H]^+$	188.0666	126.0293/123.0547/144.0396	ACN
268	Hydroxy ipronidazole	35175-14-5	$C_7H_{11}N_3O_3$	3.6	$[M+H]^+$	186.0873	168.0769/121.0753/122.0838	MeOH
269	Hydroxy dimetridazole	936-05-0	$C_5H_7N_3O_3$	2.35	$[M+H]^+$	158.056	140.0448/55.0434/69.0457	MeOH
270	Oxymetholone	434-07-1	$C_{21}H_{32}O_3$	12.69	$[M+H]^+$	333.2424	99.0439/145.0997/133.1016	ACN
271	Penicillin G potassium	113-98-4	$C_{16}H_{18}N_2O_4S$	6.53	$[M+H]^+$	335.106	114.0368/176.0707/160.0430	ACN/H_2O（1:3）
272	Penicillin V potassium salt	132-98-9	$C_{16}H_{18}N_2O_5S$	7.26	$[M+H]^+$	351.1009	160.042/114.0371/192.0655	ACN/H_2O（1:9）
273	Hydrocortisone	50-23-7	$C_{21}H_{30}O_5$	6.35	$[M+H]^+$	363.2166	327.1955/121.065/309.1846	ACN
274	Hydrocortisone 17-butyrate	13609-67-1	$C_{25}H_{36}O_6$	9.75	$[M+H]^+$	433.2585	345.2069/327.1964/121.0653	ACN
275	Hydrocortisone 17-valerate	57524-89-7	$C_{26}H_{38}O_6$	10.7	$[M+H]^+$	447.2741	345.2048/121.0647/327.1944	ACN
276	Fenoterol hydrobromide	1944-12-3	$C_{17}H_{22}BrNO_4$	2.39	$[M+H]^+$	304.1543	107.0487/135.0797/286.1402	MeOH
277	Triamcinolone acetonide	76-25-5	$C_{24}H_{31}FO_6$	8.05	$[M+H]^+$	435.2177	213.1282/339.1615/171.0827	ACN
278	Triamcinolone	124-94-7	$C_{21}H_{27}FO_6$	10.74	$[M+H]^+$	395.1864	275.1429/259.1108/317.1546	ACN
279	Troglitazone	97322-87-7	$C_{24}H_{27}NO_5S$	12.15	$[M+H]^+$	442.1683	165.091/137.0961/191.1061	MeOH
280	Desmethyl sibutramine	168835-59-4	$C_{16}H_{24}ClN$	7.26	$[M+H]^+$	266.167	125.015/139.0303/153.0462	MeOH
281	Dehydro lovastatin	109273-98-5	$C_{24}H_{34}O_4$	15.13	$[M+H]^+$	387.253	199.1486/173.1327/143.0857	MeOH
282	Boldenone	846-48-0	$C_{19}H_{26}O_2$	8.57	$[M+H]^+$	287.2006	121.0651/135.1165/173.0955	ACN
283	Ethinyl estradiol	57-63-6	$C_{20}H_{24}O_2$	9.56	$[M+H]^+$	279.1849	107.0496/265.0134/248.9826	ACN
284	Quinestrol	152-43-2	$C_{25}H_{32}O_2$	16.09	$[M+H]^+$	365.2475	297.1853/107.0495/279.1742	ACN
285	Norethindrone	68-22-4	$C_{20}H_{26}O_2$	9.45	$[M+H]^+$	299.2006	231.1742/109.0649/281.1902	ACN
286	Trenbolone	10161-33-8	$C_{18}H_{22}O_2$	8.13	$[M+H]^+$	271.1693	253.1581/199.1111/227.1427	MeOH
287	Repaglinde	135062-02-1	$C_{27}H_{36}N_2O_4$	8.63	$[M+H]^+$	453.2748	230.1893/174.1278/162.1271	ACN
288	Secnidazole	3366-95-8	$C_7H_{11}N_3O_3$	2.9	$[M+H]^+$	186.0873	168.0769/121.0761/122.0838	MeOH
289	Thiabendazole	148-79-8	$C_{10}H_7N_3S$	2.8	$[M+H]^+$	202.0433	175.0322/131.06/65.0384	ACN
290	Cyproheptadine	41354-29-4	$C_{21}H_{21}N$	6.75	$[M+H]^+$	288.1747	191.0955/215.0859/96.0810	ACN
291	Diminazene	536-71-0	$C_{14}H_{15}N_7$	2.07	$[M+H]^+$	282.1462	119.0599/102.0333/135.0785	MeOH/H_2O（1:4）
292	Ketotriclabendazole	1201920-88-8	$C_{13}H_7Cl_3N_2O_2$	10.01	$[M+H]^+$	328.9646	181.9882/168.008/98.9856	ACN
293	Triclabendazole	68786-66-3	$C_{14}H_9Cl_3N_2OS$	11.95	$[M+H]^+$	358.9574	196.9571/341.9166/343.9341	MeOH
294	Triclabendazole sulfone	106791-37-1	$C_{14}H_9Cl_3N_2O_3S$	11.3	$[M+H]^+$	390.9472	309.9459/242.0235/311.9608	MeOH
295	Triazolam	28911-01-5	$C_{17}H_{12}Cl_2N_4$	8.09	$[M+H]^+$	343.0512	308.0822/315.0325/239.0381	ACN
296	Salbutamol	18559-94-9	$C_{13}H_{21}NO_3$	2.14	$[M+H]^+$	240.1594	148.0752/166.0859/222.1485	ACN

Number	Compounds	CAS	Formula	RT（min）	Adduct	Parent ion（m/z）	Fragment ions（m/z）	Solvent
297	Sarafloxacin	98105-99-8	$C_{20}H_{17}F_2N_3O_3$	3.86	$[M+H]^+$	386.1311	368.1212/348.1144/299.0991	ACN
298	Salmeterol	89365-50-4	$C_{25}H_{37}NO_4$	7.3	$[M+H]^+$	416.2795	232.1686/380.2577/230.1529	MeOH
299	Sulindac	38194-50-2	$C_{20}H_{17}FO_3S$	8.35	$[M+H]^+$	357.0955	233.0758/340.0928/248.0998	ACN
300	Difloxacin	98106-17-3	$C_{21}H_{19}F_2N_3O_3$	3.95	$[M+H]^+$	400.1467	382.1366/356.1573/299.099	ACN
301	Amitraz	33089-61-1	$C_{19}H_{23}N_3$	13.77	$[M+H]^+$	294.1965	163.1232/122.0969/132.0813	ACN
302	Diclofenac acid	15307-86-5	$C_{14}H_9Cl_2NO$	10.81	$[M+H]^+$	278.0134	214.0411/215.0494/178.0645	ACN
303	Diclofenac sodium	15307-79-6	$C_{14}H_{10}CI_2NNaO_2$	10.77	$[M+H]^+$	296.0240	214.0414/215.0493/179.0731	ACN
304	Dihydrotestosterone	521-18-6	$C_{19}H_{30}O_2$	10.52	$[M+H]^+$	291.2319	255.2109/273.2216/159.1165	ACN
305	Sparfloxacin	110871-86-8	$C_{19}H_{22}F_2N_4O_3$	4	$[M+H]^+$	393.1733	349.1844/292.1249/251.0867	ACN
306	Stanozolol	10418-03-8	$C_{21}H_{32}N_2O$	9.25	$[M+H]^+$	329.2587	313.1691/208.1119/284.1435	ACN
307	Tetracycline	60-54-8	$C_{22}H_{24}N_2O_8$	3.28	$[M+H]^+$	445.1605	428.1349/321.0761/339.0867	ACN
308	Tylosin	1401-69-0	$C_{46}H_{77}NO_{17}$	6.71	$[M+H]^+$	916.5264	772.4484/174.1122/101.0599	ACN/H_2O（1:3）
309	Tiamulin	55297-95-5	$C_{28}H_{47}NO_4S$	7.05	$[M+H]^+$	494.3299	192.1052/119.0169/163.1117	MeOH
310	Terbutaline	23031-25-6	$C_{12}H_{19}NO_3$	2.14	$[M+H]^+$	226.1438	152.0707/125.0594/170.0813	ACN
311	Tilmicosin	108050-54-0	$C_{46}H_{80}N_2O_{13}$	5.19	$[M+H]^+$	869.5733	696.4632/174.1119/132.1017	ACN/H_2O（1:3）
312	Tenoxicam	59804-37-4	$C_{13}H_{11}N_3O_4S_2$	5.07	$[M+H]^+$	338.0264	121.04/78.0348/95.0612	ACN
313	Tinidazole	19387-91-8	$C_8H_{13}N_3O_4S$	3.3	$[M+H]^+$	248.07	121.0321/128.0457/93.0007	ACN
314	Tizanidine	51322-75-9	$C_9H_8ClN_5S$	2.35	$[M+H]^+$	254.0262	156.9619/209.9878/185.9885	MeOH
315	Ketoprofen	22071-15-4	$C_{16}H_{14}O_3$	8.75	$[M+H]^+$	255.1016	105.0328/209.0956/77.0385	ACN
316	Cephalexin	15686-71-2	$C_{16}H_{17}N_3O_4S$	2.74	$[M+H]^+$	348.1013	158.0269/174.0549/106.0653	ACN/H_2O(1:3)
317	Cefotaxime	63527-52-6	$C_{16}H_{17}N_5O_7S_2$	3.2	$[M+H]^+$	456.0642	324.0585/396.0432/125.0046	ACN/H_2O(1:3)
318	Cefaclor	53994-73-3	$C_{15}H_{14}ClN_3O_4S$	3.4	$[M+H]^+$	368.0466	174.0551/106.0649/118.0408	ACN/H_2O(1:3)
319	Cefixime	79350-37-1	$C_{16}H_{15}N_5O_7S_2$	3.19	$[M+H]^+$	454.0486	285.0294/126.0119/210.0206	ACN/H_2O(1:3)
320	Cefquinome	84957-30-2	$C_{23}H_{24}N_6O_5S_2$	2.67	$[M+H]^+$	529.1322	134.096/167.0264/324.0571	ACN/H_2O(1:3)
321	Cephradine	38821-53-3	$C_{16}H_{19}N_3O_4S$	2.95	$[M+H]^+$	350.1169	192.0465/160.0418/174.0541	ACN/H_2O(1:3)
322	Cephalonium	5575-21-3	$C_{20}H_{18}N_4O_5S_2$	2.89	$[M+H]^+$	459.0791	152.0158/123.0549/185.0372	ACN/H_2O(1:3)
323	Cefamandole	34444-01-4	$C_{18}H_{18}N_6O_5S_2$	4.96	$[M+H]^+$	463.0853	158.0264/140.0156/185.0373	ACN/H

Number	Compounds	CAS	Formula	RT（min）	Adduct	Parent ion（m/z）	Fragment ions（m/z）	Solvent
								$_2$O(1:3)
324	Cefminox	75481-73-1	$C_{16}H_{21}N_7O_7S_3$	2.22	$[M+H]^+$	520.0737	161.0375/328.0425/215.0481	ACN/H$_2$O(1:3)
325	Cefoperazone	62893-19-0	$C_{25}H_{27}N_9O_8S_2$	4.62	$[M+H]^+$	646.1497	143.0811/530.135/290.1129	ACN/H$_2$O(1:3)
326	Cephapirin	21593-23-7	$C_{17}H_{17}N_3O_6S_2$	2.34	$[M+H]^+$	424.0632	292.0588/152.0161/124.0213	ACN/H$_2$O(1:3)
327	Cefpirome	84957-29-9	$C_{22}H_{22}N_6O_5S_2$	2.41	$[M+H]^+$	515.1166	120.08/167.0261/140.0153	ACN/H$_2$O(1:3)
328	Cefadroxil	50370-12-2	$C_{16}H_{17}N_3O_5S$	2.2	$[M+H]^+$	364.0962	114.0001/134.0355/68.0501	ACN/H$_2$O(1:3)
329	Ceftiofur	80370-57-6	$C_{19}H_{17}N_5O_7S_3$	5.56	$[M+H]^+$	524.0363	241.0397/125.0043/126.0121	ACN/H$_2$O(1:3)
330	Ceftazidime	72558-82-8	$C_{22}H_{22}N_6O_7S_2$	2.32	$[M+H]^+$	547.1064	167.0266/396.0779/468.0635	ACN/H$_2$O(1:3)
331	Cefetamet pivoxil	65243-33-6	$C_{20}H_{25}N_5O_7S_2$	8.45	$[M+H]^+$	512.1268	241.038/398.0598/482.1173	ACN/H$_2$O(1:3)
332	Cefazolin	25953-19-9	$C_{14}H_{14}N_8O_4S_3$	3.67	$[M+H]^+$	455.0373	323.0562/156.0108/153.0477	ACN/H$_2$O(1:3)
333	Oxytetracycline	79-57-2	$C_{22}H_{24}N_2O_9$	2.99	$[M+H]^+$	461.1555	426.1178/337.0713/201.0526	ACN
334	Melatonine	73-31-4	$C_{13}H_{16}N_2O_2$	4.87	$[M+H]^+$	233.1285	174.0916/130.0653/159.0674	MeOH
335	Tolmetin	26171-23-3	$C_{15}H_{15}NO_3$	8.32	$[M+H]^+$	258.1125	119.0488/91.054/166.0495	ACN
336	Tolfenamic acid	13710-19-5	$C_{14}H_{12}ClNO_2$	12.28	$[M+H]^+$	262.0629	244.0525/209.083/180.0805	ACN
337	Dehydroepiandrosterone	53-43-0	$C_{19}H_{28}O_2$	9.98	$[M+H]^+$	289.2162	271.2055/109.0646/253.1951	ACN
338	Anhydroerythromycin A	23893-13-2	$C_{37}H_{65}NO_{12}$	7	$[M+H]^+$	716.458	558.3648/158.1176/540.3534	MeOH
339	Desoxycarbadox	55456-55-8	$C_{11}H_{10}N_4O_2$	5.07	$[M+H]^+$	231.0877	199.0616/143.0606/171.0666	MeOH
340	Cortexolone	152-58-9	$C_{21}H_{30}O_4$	8	$[M+H]^+$	347.2217	109.0964/329.2121/97.0651	ACN
341	Tulobuterol	41570-61-0	$C_{12}H_{18}ClNO$	3.58	$[M+H]^+$	228.115	154.0414/118.0655/119.0732	MeOH
342	Toltrazuril	69004-03-1	$C_{18}H_{14}F_3N_3O_4S$	11.93	$[M+H]^+$	426.073	192.9918/164.9966/115.0003	ACN
343	Toltrazuril sulfoxide	69004-15-5	$C_{18}H_{14}F_3N_3O_5S$	9.11	$[M+H]^+$	442.0679	373.0726/233.08/133.0521	MeOH
344	Vildagliptin	274901-16-5	$C_{17}H_{25}N_3O_2$	2.28	$[M+H]^+$	304.202	154.0968/97.076/151.111	MeOH
345	Virginiamycin M1	21411-53-0	$C_{28}H_{35}N_3O_7$	8.5	$[M+H]^+$	526.2548	508.2441/355.1292/337.1178	MeOH
346	Venlafaxine	93413-69-5	$C_{17}H_{27}NO_2$	4.62	$[M+H]^+$	278.2115	260.2016/58.0672/121.065	MeOH
347	Sildenafil	139755-83-2	$C_{22}H_{30}N_6O_4S$	5.93	$[M+H]^+$	475.2122	58.0671/283.1183/311.1511	MeOH
348	Cimaterol	54239-37-1	$C_{12}H_{17}N_3O$	2.16	$[M+H]^+$	220.1444	143.0609/160.0873/116.0506	ACN
349	Cinoxacin	28657-80-9	$C_{12}H_{10}N_2O_5$	4.84	$[M+H]^+$	263.0663	245.0557/217.0607/189.0293	MeOH
350	Sitagliptin	486460-32-6	$C_{16}H_{15}F_6N_5O$	4.16	$[M+H]^+$	408.1254	235.0805/174.0525/193.0696	MeOH
351	Nifedipine	21829-25-4	$C_{17}H_{18}N_2O_6$	9.17	$[M+H]^+$	347.1238	122.0252/222.0764/254.1042	MeOH

Number	Compounds	CAS	Formula	RT（min）	Adduct	Parent ion（m/z）	Fragment ions（m/z）	Solvent
352	Nitrazepam	146-22-5	$C_{15}H_{11}N_3O_3$	7.14	$[M+H]^+$	282.0873	236.0947/180.0804/207.0916	ACN
353	Simvastatin	79902-63-9	$C_{25}H_{38}O_5$	13.83	$[M+H]^+$	419.2792	199.1478/285.204/225.1634	ACN
354	Androsterone	53-41-8	$C_{19}H_{30}O_2$	11.2	$[M+H]^+$	291.2319	255.21/159.1171/145.1003	ACN
355	Bromchlorbuterol	37153-52-9	$C_{12}H_{18}BrClN_2O$	3.94	$[M+H]^+$	321.0364	246.963/168.045/303.0264	MeOH
356	Ofloxacin	82419-36-1	$C_{18}H_{20}FN_3O_4$	3.08	$[M+H]^+$	362.1511	318.1613/261.1033/344.1405	ACN
357	Enoxacin	74011-58-8	$C_{15}H_{17}FN_4O_3$	2.93	$[M+H]^+$	321.1358	303.1246/232.0514/204.0561	ACN
358	Eprinomectin	123997-26-2	$C_{50}H_{75}NO_{14}$	15.05	$[M+H]^+$	914.526	186.1126/154.0863/330.1926	ACN
359	Megestrol acetate	595-33-5	$C_{24}H_{32}O_4$	12.32	$[M+H]^+$	385.2373	267.1746/224.1557/325.2172	ACN
360	Mequindox	13297-17-1	$C_{11}H_{10}N_2O_3$	3.54	$[M+H]^+$	219.0764	143.0602/185.0707/160.0626	ACN
361	Ipronidazole	14885-29-1	$C_7H_{11}N_3O_2$	4.89	$[M+H]^+$	170.0924	109.0776/124.1008/123.0925	ACN
362	Isochlortetracycline	514-53-4	$C_{22}H_{23}ClN_2O_8$	3.71	$[M+H]^+$	479.1216	462.0138/196.92/416.1	ACN
363	Indomethacin	53-86-1	$C_{19}H_{16}ClNO_4$	10.91	$[M+H]^+$	358.0841	138.9939/110.999/174.0914	ACN
364	Leucomalachite green	129-73-7	$C_{23}H_{26}N_2$	8.09	$[M[^+$	331.2169	239.1542/315.1863/316.194	MeOH
365	Leucocrystal violet	603-48-5	$C_{25}H_{31}N_3$	4.77	$[M]^+$	374.2591	358.227/239.1533/238.1455	MeOH
366	Indoprofen	31842-01-0	$C_{17}H_{15}NO_3$	7.79	$[M+H]^+$	282.1125	236.1072/218.0965/180.081	ACN
367	Progesterone	57-83-0	$C_{21}H_{30}O_2$	12.28	$[M+H]^+$	315.2319	109.0647/97.0649/297.2205	ACN
367	Pregnenolone	145-13-1	$C_{21}H_{32}O_2$	12.06	$[M+H]^+$	317.2475	299.2374/281.2258/159.1169	ACN
369	Zaleplon	151319-34-5	$C_{17}H_{15}N_5O$	7.15	$[M+H]^+$	306.1349	236.0952/219.0683/234.0796	MeOH
370	Oleandomycin	3922-90-5	$C_{35}H_{61}NO_{12}$	5.69	$[M+H]^+$	688.4267	544.3473/158.1172/116.0709	MeOH
371	Levamisole	14769-73-4	$C_{11}H_{12}N_2S$	2.61	$[M+H]^+$	205.0794	178.0687/123.0266/91.0548	ACN
372	Levofloxacine	100986-85-4	$C_{18}H_{20}FN_3O_4$	3.01	$[M+H]^+$	362.1511	261.1025/318.1606/344.1398	MeOH
373	Zolpidem	82626-48-0	$C_{19}H_{21}N_3O$	4.48	$[M+H]^+$	308.1757	236.1287/235.1232/263.1157	MeOH
374	3,5-Dinitrobenzamide	121-81-3	$C_7H_5N_3O_5$	4.41	$[M-H]^-$	210.0156	61.9882/63.0236/89.9247	MeOH
375	ez-Diethylstilbestrol	6898-97-1	$C_{18}H_{22}O_2$	10.62	$[M-H]^-$	269.1547	119.0499/133.0657/120.0521	MeOH
376	Analgin	68-89-3	$C_{13}H_{16}N_3NaO_4S$	4.45	$[M-H]^-$	310.0867	79.9610/175.0194/80.9680	ACN
377	Barbital	57-44-3	$C_8H_{12}N_2O_3$	3.29	$[M-H]^-$	183.0775	61.9878/69.0703/97.0288	MeOH
378	Phenobarbital	50-06-6	$C_{12}H_{12}N_2O_3$	5.2	$[M-H]^-$	231.0775	61.9882/164.9981/87.9247	MeOH
379	Ibuprofen	15687-27-1	$C_{13}H_{18}O_2$	10.85	$[M-H]^-$	205.1234	161.1320/119.0845/105.0695	MeOH
380	Dapagliflozin	461432-26-8	$C_{21}H_{25}ClO_6$	8.02	$[M-H]^-$	407.1267	167.0254/135.0802/191.0255	MeOH
381	Diclazuril	101831-37-2	$C_{17}H_9Cl_3N_4O_2$	11.31	$[M-H]^-$	404.9718	333.9724/298.9791/334.9559	MeOH
382	Diflunisal	22494-42-4	$C_{13}H_8F_2O_3$	9.77	$[M-H]^-$	249.0369	205.0484/177.0505/206.0502	MeOH
383	Sodium nifurstylenate	54992-23-3	$C_{13}H_8NNaO_5$	8.92	$[M-Na]^-$	258.0408	114.0473/214.0510/108.0211	MeOH
384	Furosemide	54-31-9	$C_{12}H_{11}ClN_2O_5S$	6.82	$[M-H]^-$	329.0004	204.9850/285.0108/77.9654	MeOH
385	Florfenicol	73231-34-2	$C_{12}H_{14}Cl_2FNO_4S$	4.79	$[M-H]^-$	355.9532	119.0554/185.0278/78.9859	MeOH
386	Flurbiprofen	5104-49-4	$C_{15}H_{13}FO_2$	10.38	$[M-H]^-$	243.0827	61.9880/59.9858/197.9049	MeOH

Number	Compounds	CAS	Formula	RT（min）	Adduct	Parent ion（m/z）	Fragment ions（m/z）	Solvent
387	Ciglitazone	74772-77-3	$C_{18}H_{23}NO_3S$	14.13	$[M-H]^-$	332.1326	150.0141/289.1254/149.0074	ACN
388	Hexestrol	84-16-2	$C_{18}H_{22}O_2$	10.56	$[M-H]^-$	269.1547	253.1596/199.1115/165.0700	ACN
389	Diethylstilbestrol	56-53-1	$C_{18}H_{20}O_2$	10.34	$[M-H]^-$	267.1391	237.0913/251.1069/222.0679	ACN
390	Narasin	55134-13-9	$C_{43}H_{72}O_{11}$	19.47	$[M-H]^-$	763.5002	255.1594/407.2432/745.4926	MeOH
391	Lasalocid	25999-31-9	$C_{34}H_{54}O_8$	17.71	$[M-H]^-$	589.3746	235.0984/337.2750/237.1858	ACN
392	Monensin	17090-79-8	$C_{36}H_{62}O_{11}$	18.34	$[M-H]^-$	669.4219	637.3984/137.0970/101.0606	MeOH
393	Nicarbazin	330-95-0	$C_{19}H_{18}N_6O_6$	10.46	$[M-H]^-$	301.0578	137.0370/107.0377/93.0570	ACN/N，N-DMF（9:1）
394	Hydrochlorothiazide	58-93-5	$C_7H_8ClN_3O_4S_2$	2.96	$[M-H]^-$	295.9572	268.9468/204.9846/77.9652	MeOH
395	Sulbactam	68373-14-8	$C_8H_{11}NO_5S$	2.3	$[M-H]^-$	232.0285	69.9626/140.0713/188.0393	ACN
396	Dienestrol	84-17-3	$C_{18}H_{18}O_2$	10.46	$[M-H]^-$	265.1234	93.0355/249.0925/117.0341	ACN
397	Secobarbital	76-73-3	$C_{12}H_{18}N_2O_3$	7.39	$[M-H]^-$	237.1245	41.9985/194.1188/42.0085	MeOH
398	Ponazuril	69004-04-2	$C_{18}H_{14}F_3N_3O_6S$	10.77	$[M-H]^-$	456.0483	162.8385/127.8709/148.0391	MeOH
399	Nitroxynil	1689-89-0	$C_7H_3IN_2O_3$	7.67	$[M-H]^-$	288.9116	126.9061/162.0067/258.9126	ACN
400	Salinomycin	53003-10-4	$C_{42}H_{70}O_{11}$	18.57	$[M-H]^-$	749.4845	241.1440/407.2434/731.4773	MeOH
401	Amobarbital	57-43-2	$C_{11}H_{18}N_2O_3$	6.76	$[M-H]^-$	225.1245	143.8634/141.8680/61.9881	MeOH
402	4,4′-Dinitrocarbanilide	587-90-6	$C_{13}H_{10}N_4O_5$	10.43	$[M+H]^+$	303.0724	93.0570/122.0466/139.0493	ACN
403	Propetamphos	31218-83-4	$C_{10}H_{20}NO_4PS$	11.49	$[M+H]^+$	282.0923	138.0128/156.023/109.9813	ACN
404	Fenthion	55-38-9	$C_{10}H_{15}O_3PS_2$	12.51	$[M+H]^+$	279.0273	169.0118/105.0691/124.9812	ACN
405	Ricinine	524-40-3	$C_8H_8N_2O_2$	2.62	$[M+H]^+$	165.0659	138.0545/82.0299/84.0456	ACN
406	Trichlorfon	52-68-6	$C_4H_8Cl_3O_4P$	3.75	$[M+H]^+$	256.9299	109.0043/256.93/220.9531	ACN
407	Dichlorvos	62-73-7	$C_4H_7Cl_2O_4P$	6.42	$[M+H]^+$	220.9532	109.0032/78.9935/127.0148	ACN
408	Fluazuron	86811-58-7	$C_{20}H_{10}Cl_2F_5N_3O_3$	14.52	$[M+H]^+$	506.0092	348.9751/158.0407/141.0142	MeOH
409	Diazinon	333-41-5	$C_{12}H_{21}N_2O_3PS$	12.63	$[M+H]^+$	305.1083	169.0789/153.1019/249.0453	ACN
410	Malathion	121-75-5	$C_{10}H_{19}O_6PS_2$	11.29	$[M+H]^+$	331.0433	99.0064/124.9813/127.0383	MeOH
411	Phoxim	14816-18-3	$C_{12}H_{15}N_2O_3PS$	13.36	$[M+H]^+$	299.0614	77.0381/96.9497/129.0435	AC
412	Ethopabate	59-06-3	$C_{12}H_{15}NO_4$	5.74	$[M+H]^+$	238.1074	206.0808/136.0395/164.0704	MeOH
413	Salicylic acid	69-72-7	$C_7H_6O_3$	4.18	$[M-H]^-$	137.0244	65.0398/121.0275/93.0327	MeOH
414	Sodium pentachlorophenoxide	131-52-2	C_6Cl_5NaO	12.09	$[M-H]^-$	264.83	1999.8759/227.8698/96.9890	ACN
415	α-Zearalenol	36455-72-8	$C_{18}H_{24}O_5$	9	$[M-H]^-$	319.1551	275.1655/174.0318/238.8923	ACN
416	β-Zearalanol	42422-68-4	$C_{18}H_{26}O_5$	7.96	$[M-H]^-$	321.1708	277.1780/303.1591/259.1702	ACN
417	β-Zearalenol	71030-11-0	$C_{18}H_{24}O_5$	8.08	$[M-H]^-$	319.1551	275.1651/174.0304/301.1437	ACN
418	Zearalanol	26538-44-3	$C_{18}H_{26}O_5$	8.81	$[M-H]^-$	321.1708	277.1811/303.1595/259.1691	ACN
419	Zearalanone	5975-78-0	$C_{18}H_{24}O_5$	10.04	$[M-H]^-$	319.1551	275.1461/205.0724/161.0855	ACN

Number	Compounds	CAS	Formula	RT（min）	Adduct	Parent ion（m/z）	Fragment ions（m/z）	Solvent
420	Zearalenone	17924-92-4	$C_{18}H_{22}O_5$	10.11	$[M-H]^-$	317.1395	185.0587/187.0743/203.0690	ACN

Table S2. Information of 20 compounds and 20 matrices

Compound	Matrix	Number
Leucomalachite green, Megestrol acetate, Cloxacillin, Glipizide, Methylprednisolone, Cortisone, Prednisolone, Ethopabate, Fenfluramine, Carazolol, Sulfamethoxazole, Oxibendazole, Clindamycin, 5-hydroxymebendazole, Dinitolmide, Formoterol, Ractopamine, Cefotaxime, Sulfadiazine, Furaltadone.	Pig formula feed	2
	Chicken formula feed	2
	Pig concentrated feed	2
	Chicken concentrated feed	2
	Cow concentrate supplement	2
	Composite additive premix	2
	Food flavour	2
	Plant extracts (Radix Astragali)	2
	Trace mineral feed	2
	Vitamin premix feed	2

Table S3. Information of 248 real samples

Sample type	Number	Subtype
Feed raw materials	40	Torreyaes emen exact, Motherwort herb extract, Astragalus root extract, Dark plum extract, Agastache rugosus, Liquorice, Fructus Crataegi, Heartleaf houttuynia herb, Japanese honeysuckle flower bud extract， Dandelion extract， Distillers dried grains with solubles, Rice bran meal, Hydrolyaed yeast extract, Motherwort herb extract, Angelica (Dong Quai) extract, Swordlike atractylodes rhizome extract, Aspen flower extract, Balloon flower root extract, Gotu kola extract, Eucmmia leaf extract, Curcuma longaextract
Vitamin premix feed	21	Vitamin premix feed for fattening pigs, Vitamin premix feed for piglets, Vitamin premix feed for livestock and poultry, 0.02% Vitamin premix feed for laying hens
Trace element premix feed	11	0.1% Trace element premix feed for breeding chickens, 0.1% Trace element premix feed for pigs, 0.03% 0.1% Trace element premix feed for piglets, 0.15% Trace element premix feed for breeding chickens
Composite premix feed	29	0.5% Composite premix feed for piglets, Composite premix feed for fattening pigs, Composite premix feed for livestock and poultry, 5% Composite premix feed for breeding chickens, Composite premix feed for laying hens, Composite premix feed for carp
Concentrated feed	1	Concentrated feed for pigs

Feed additives	146	Gallotannic acid, Bacillus subtilis, Bacillus licheniformis, protease, Iron Proteinate, lactobacillus plantarum, Flavouring agent, Hydroxymethionine, Calcium hydrogen phosphate, L-threonine, Lysine, Copper sulfate, Common salt, Phytase, isoleucine, Ammonia chloride, Magnesium oxide, Zinc amino acid complex, Saccharomyces cerevisiae type I, L-ascorbic acid， Compound microbial preparation type I-C, Compound acidity regulator, acidity regulator, Zymin and Microbiology, Calcium phosphorus zinc, Vitamin B2 and nicotinamide, Bacillus albacter and Clostridium butyricum, Bacillus subtilis and Saccharomyces cerevisiae type I, Bacillus subtilis and Cellulase type II, Candida subtilis and Bacillus, subtilis and Streptococcus lactate, Lipase, High-temperature resistant phytase LS-P816, Propionic acid and alanine, Bile acid

Table S4. SDL and Precision test results of 420 risk substances

Number	Compounds	SDL（μg/L）	Intra-day RSD（%）			Inter-day RDD（%）		
			50（μg/L)	100（μg/L)	500（μg/L)	50 （μg/L)	100（μg/L)	500（μg/L)
1	2-Aminoflubendazole	50	5.76	3.02	4.81	14.89	11.86	4.56
2	2-Methyl-5-nitroimidazole	50	2.76	6.04	4.57	4.72	5.4	7.15
3	2-NP-SEM	50	2.14	4.79	8.26	8.62	11.09	8.43
4	3-Methyl-quinoxaline-2-carboxylic acid	50	4.56	2.47	2.75	4.58	14.76	5.63
5	Desacetyl cefotaxime	100	-	5.33	4.67	-	10.07	9.07
6	4-Aminoantipyrine	50	8.77	2.48	5.77	21.57	3.29	4.38
7	4-Formylaminoantipyrine	50	3.4	8.93	7.94	2.68	10.84	2.26
8	4-Isopropylaminoantipyrine	50	4.03	4.51	7.63	2.85	0.3	7.73
9	5-Hydroxymebendazole	100	-	8.81	9.44	-	7.61	0.81
10	5-Hydroxythiabendazole	50	7.57	6.77	6.63	17.83	6.59	2.7
11	17α-Estradiol	50	5.94	6.37	3.12	23.36	8.53	8.38
12	17α-Hydroxyprogesterone	50	7.87	6.03	8.7	21.87	4.38	1.69
13	(22R)-Budesonide	50	9.12	5.64	8.98	20.57	8.69	3.01
14	α-Trenbolone	50	7.36	6.2	9.7	13.82	1.37	0.69
15	N-Didesmethyl Sibutramine	50	7.08	2.22	2.42	12.98	0.33	7.62
16	Albendazole sulfone	50	3.87	8.76	5.55	2.06	6.67	4.91
18	Albendazole sulfoxide	50	3.83	8.85	3.47	3.63	10.54	0.37
18	Albendazolesulf oxide	50	4.36	8.27	9.01	7.15	2.22	8.96
19	Albendazole-2-aminosulfone	50	6.82	6.01	5.86	17.45	1.47	2.72
20	Aklomide	50	8.72	6.37	8.97	15.16	5.07	4.98
21	Alclomethasone dipropionate	50	5.33	4.08	9.6	20.29	5.24	3.63
22	Alprazolam	50	3.02	9.05	7.39	17.37	13.98	6.72
23	Atenolol	50	8.19	4.35	9.03	9.82	3.07	5.71

Number	Compounds	SDL （μg/L）	Intra-day RSD（%）			Inter-day RDD（%）		
			50（μg/L）	100（μg/L）	500（μg/L）	50 （μg/L）	100 （μg/L）	500（μg/L）
24	Atropine	50	5.41	2.12	4.21	17.33	3.11	5.37
25	Estazolam	50	7.12	5.01	3.82	16.77	4.28	6.72
26	Methaqualone	50	4.66	4.71	6.5	8.37	7.54	7.87
27	Antipyrine	50	5.81	8.41	7.97	14.62	3.51	1.75
28	Amcinonide	50	5.68	6.62	5.9	8.64	9	2.61
29	Aminophylline	50	3.41	4.12	3.19	20.23	13.27	6.88
30	Mebendazole amine	50	9.4	7.13	8.01	6.03	12.63	3.01
31	Amino tadalafil	50	5.72	8.02	7.7	21.76	11.51	1.73
32	Tranexamic acid	50	5.99	5.25	6.04	3.73	7.44	7.41
33	Amlodipine	50	7.01	6.69	7.72	9.23	8.05	8.49
34	Orbifloxacin	50	6.79	6.73	7.18	17.57	2.03	0.99
35	Oxfendazole	50	8.39	8.05	7.63	13.32	0.15	7.04
36	Olaquindox	50	6.91	6.68	2.53	15.87	0.84	2.06
37	Oxazepam	50	6.38	3.99	4.65	2.11	11.75	2.74
38	Oseltamivir	50	6.7	8.79	5.68	20.84	4.62	3.75
39	Ornidazole	50	7.97	7.09	6.94	7.85	11.39	4.97
40	Baclofen	50	9.09	5.64	3.95	3.46	7.09	4.55
41	Beclomethasone	50	3.41	7.69	2.24	15.62	11.85	5.08
42	Beclomethasone dipropionate	50	9.43	5.03	8.45	16.54	8.27	8.49
43	Betamethasone	50	7.62	5.08	9.04	16.02	6.23	4.18
44	Betamethasone dipropionate	50	7.28	7.84	9.92	18.26	13.62	2.94
45	Betamethasone 17-valerate	50	6.44	2.76	9.07	23.71	7.74	6.4
46	Clobetasone butyrate	50	5.31	5.24	5.74	4.39	6.56	6.35
47	Nadrolone phenylpropionate	50	5.02	5.04	5.95	4.69	8.64	2.4
48	Benzimidazole	50	2.61	6	2.16	15.57	5.65	7.78
49	Sulfabenzamide	50	6.93	4.95	7.57	15.98	5.47	4.15
50	Febantel	50	6.7	5.31	8.57	20.81	5.8	8.46
51	Azlocillin	50	4.71	5.33	2.09	7.57	9.71	8.94
52	5-Nitrobenzimidazole	50	8	8.99	4.94	4.69	3.09	0.41
53	Isoxsuprine	50	5.05	3.44	2.87	5.59	0.33	0.08
54	Phenylethanolamine A	50	7.48	9.11	5.05	22.2	0.33	6.23
55	Phenformin	50	7.21	8.76	8.68	13.82	10.37	5.39
56	Oxacillin	50	5.9	3.82	4.57	22.45	6.39	4.46
57	Pirlimycin	50	7.6	4.66	4.27	10.28	4.5	2.33
58	Piroxicam	50	4.88	2.46	4.98	12.01	0.94	5.65
59	Epitestosterone	50	4.45	6.83	5.08	11.56	3.05	4.9
60	Testosterone propionate	50	2.65	4.21	5.98	14.87	14.08	2.06
61	Nandrolone propionate	50	4.45	4.85	4.05	15.6	4.85	7.5
62	Oxibendazole	50	4.93	5.31	4.94	7.83	7.71	3.86
63	Budesonide	50	5.5	4.72	7.29	21.17	6.38	9.19

Number	Compounds	SDL	Intra-day RSD（%）			Inter-day RDD（%）		
		（μg/L）	50（μg/L）	100（μg/L）	500（μg/L）	50 （μg/L）	100 （μg/L）	500（μg/L）
64	Halofuginone	50	4.19	5.31	6.47	21.03	5.19	2.22
65	Estradiol	50	6.1	8.61	8.13	15.94	8.3	7.92
66	Estrone	50	8.41	5.01	5.84	7.08	2.86	3.72
67	N-Acetylsulfamethoxazole	50	9.21	7.07	5.44	22.26	5.43	8.76
68	Aceclofenac	50	6.69	5.26	4.16	5.25	6.04	5.29
69	Betamethasone 21-acetate	50	7.44	7.44	2.21	7.33	5.16	8.67
70	Dexamethasone 21-acetate	50	4.13	4.51	7.09	3.04	6.64	1.07
71	Fluorometholone 17-Acetate	50	6.35	6.67	6.98	8.92	8.91	0.06
72	Fludrocortisone 21-acetate	50	6.09	4.79	6.38	13.12	12.12	0.59
73	Flugestone acetate	50	3.74	6.53	5.01	8.96	3.21	1.55
74	Cyproterone acetate	50	8.34	7.65	5.45	2.74	2.75	4.03
75	Medroxyprogesterone 17-acetate	50	6.23	4.61	9.49	12	3.73	6.7
76	Cortisone 21-acetate	50	7.43	6.18	3.09	20.66	9.15	3.4
77	Chlormadinone acetate	50	8.82	2.17	3.26	21.68	12.71	2.59
78	Melengestrol acetate	50	6.57	2.05	5.1	16.86	11.05	5.71
79	Prednisone 21-acetate	50	2.88	3.06	2.02	4.8	7.6	6.57
80	Prednisolone 21-acetate	50	2.02	6.5	6.74	17.09	4.61	2.6
81	17α-Hydroxyprogesterone acetate	50	2.08	6.48	6.45	18.23	5.43	3.16
82	Hydrocortisone acetate	50	2.53	6.94	6.05	15.3	1.53	6.05
83	Triamcinolone acetonide acetate	50	7.45	3.29	4.31	22.21	10.12	7.02
84	Triamcinolone diacetate	50	4.87	5.37	3.72	19.15	2.71	8.18
85	Norethisterone acetate	50	2.32	2.93	7.97	16.25	6.67	0.35
86	Danofloxacin	50	6.1	4.27	2.16	12.58	5.71	6.72
87	Azaperone	50	3.22	8.78	6.5	3.91	10.45	8.85
88	Deflazacort	50	7.84	7.58	4.01	13.13	11.72	5.51
89	Dexamethasone	50	6.2	3.31	5.39	19.37	4.3	5.8
90	Diazepam	50	4.55	4.63	5.49	17.71	2.79	3.99
91	Dicyclanil	50	5.62	6.82	6.07	20.43	8.66	1.04
92	Buquinolate	100	-	3.7	4.11	-	0.52	7.71
93	Sultamicillin tosilate	50	7.41	5.87	8.12	12.19	3.55	6.61
94	Acetaminophen	50	2.11	5.93	7.37	20.4	13.44	2.25
95	Doramectin	100	-	5.79	4.13	-	5.45	4.08
96	Doxycycline	50	6.15	8.72	9.64	4.31	10.65	6.08
97	Oxolinic acid	50	3.2	7.08	8.68	21.89	10.2	7.83
98	Enrofloxacin	50	4.12	3.56	3.78	5.54	0.35	3.76
99	Diflorasone diacetate	50	8.99	7.37	4.33	19.22	6.62	8.5
100	Dimetridazole	50	8.89	5.61	4.17	10.99	3.06	6.1
101	Dinitolmide	50	9.43	5.73	4.32	10.78	0.36	1.16
102	Dioxopromethazine	50	6.05	4.52	3.04	11.87	7.11	4.39
103	Felodipine	50	4.72	6.43	6.36	19.2	10.01	6.22

Number	Compounds	SDL (μg/L)	Intra-day RSD（%）			Inter-day RDD（%）		
			50（μg/L）	100（μg/L）	500（μg/L）	50 （μg/L）	100 （μg/L）	500（μg/L）
104	Phenacetin	50	7.22	2.34	7.27	21.85	7.89	6.32
105	Fenbendazole	50	5.53	7.55	6.89	9.91	8.16	4.49
106	Fenbendazole sulfone	50	5.96	9.18	2.78	3.47	14.5	4.89
107	Fenfluramine	50	8.78	3.61	6.05	4.29	9.32	8.74
108	Phenolphthalein	50	4.65	3.54	2.94	5.86	13.23	5.39
109	Rimsulfuron	50	4.84	2.13	4.21	13.64	1.81	6.34
110	Furaltadone	50	9.11	5.18	3.55	20.37	14.71	3.18
111	2-NP-AMOZ	50	3.13	4.84	2.88	20.2	4.36	5.99
112	Nitrofurantoin	50	7.12	3.66	4.01	21.51	4.15	0
113	2-NP-AHD	50	7.22	3.3	6.4	12.16	8.17	1.76
114	Furazolidone	50	4.78	8.92	5.72	10.17	1.99	4.65
115	2-NP-AOZ	50	9.17	2.85	8.89	10.35	4.08	4.69
116	Flubendazole	50	4.05	5.05	6.04	5.79	7.41	0.79
117	Flufenamic acid	50	5.17	6.48	7.19	11.38	2.49	5.78
118	Flumequin	50	5.69	5.9	6.92	9.76	14.61	1.77
119	Fleroxacin	50	8.98	5.25	4.44	5.54	2.95	5.65
120	Flucloxacillin	50	9.07	6.99	2.91	12.5	14.73	6.18
121	Fluorometholone	50	4.83	8.42	7.89	11.59	9.31	6.19
122	Flumethasone	50	2.43	4.45	5.45	16.16	11.21	6.51
123	Flunixin	50	5.3	8.84	6.91	18.64	3.48	4.41
124	Haloperidol	50	6.05	9.11	4.4	5.53	10.54	0.27
125	Fluprednisolone	50	2.85	6.38	3.04	14.01	0.55	4.23
126	Fluocinolone acetonide	50	9.01	9.41	6.16	8.26	11.42	8.7
127	Fluocinonide	50	7.3	6.19	2.65	3.16	12.56	9.35
128	Fludroxycortide	50	6.52	6.98	6.19	5.8	8.39	8.38
129	Fluticasone propionate	50	9.21	5.86	6.97	8.75	12.58	7.76
130	Formoterol	50	4.93	3.1	2.03	4.7	3.45	9.21
131	Testosterone	50	4.84	8.17	6.83	7	14.75	4.56
132	Glibenclamide	50	2.28	7.55	2.39	21.8	13.1	8.82
133	Glipizide	50	6.81	6.9	2.45	11.02	0.89	8
134	Glibornuride	100	-	2.4	3.77	-	14.12	5.51
135	Gliquidone	50	2.61	4.64	2.41	11.3	9.94	1.81
136	Glimepiride	50	5.5	4.68	5.59	15.67	4.5	2.73
137	Gliclazide	50	8.3	6.31	4.1	5.2	1.03	3.72
138	Guanfacine	50	3.16	4.47	6.59	22.18	13.57	3.27
139	Decoquinate	100	-	8.59	8.9	-	3.24	6.47
140	Halcinonide	50	5.73	5.83	3.99	11.75	14.38	8.38
141	Erythromycin	50	5.53	8	7.78	16.59	1.22	4.28
142	Ciprofloxacin	50	4.04	9.24	3.75	16.82	10.85	2.72
143	Sulfaphenazole	50	2.21	7.42	4.31	4.59	7.07	6.9

Number	Compounds	SDL （μg/L）	Intra-day RSD（%）			Inter-day RDD（%）		
			50（μg/L）	100（μg/L）	500（μg/L）	50 （μg/L）	100 （μg/L）	500 （μg/L）
144	Sulfapyridine	50	6.16	6.41	3.44	4.25	11.02	8.68
145	Sulfapyrazole	50	5.75	6.9	7.29	7.9	3.52	9.04
146	Sulfacetamide	50	4	6.07	8.27	9.64	7.93	7.22
147	Sulfameter	50	4.04	4	3.85	15.12	4.98	4.13
148	Sulfamoxole	50	9.39	7.86	4.35	11.94	12.92	4.14
149	Sulfamethazine	50	8.23	4.62	5.98	18.3	6.63	7.42
150	Sulfisoxazole	50	3.39	5.61	2.27	21.78	10.14	4.9
151	Sulfisomidine	50	6.68	4.83	8.69	15.76	6.25	3.3
152	Sulfamerazine	50	9.23	9.14	8.99	22.32	3.05	8.13
153	Sulfamethoxazole	50	2.28	3.96	3.55	2.8	13.76	8.74
154	Sulfamethizole	50	2.87	3.22	3.03	19.65	5.01	4.16
155	Sulfadimethoxine	50	2.35	8.47	3.81	19.38	1.1	7.82
156	Sulfamonomethoxine	50	5.29	5.32	2.78	9.5	7.61	2.63
157	Sulfaquinoxaline	50	8.26	5.78	8.5	20.8	6.75	5.2
158	Sulfadoxine	50	4.04	2.05	3.05	15.49	10.78	0.69
159	Sulfachloropyridazine	50	4.19	7.87	3.74	13.19	5.6	7.18
160	Sulfadiazine	50	7.96	3.5	7.25	19.13	3.78	3.04
161	Sulfathiazole	50	5.57	2.69	5.34	20.65	0.81	7.67
162	Sulfanitran	50	2.4	2.29	7.57	6.15	13.04	3.91
163	Gemifioxacin	50	6.7	8.45	8.57	11.49	11.04	4.86
164	Kitasamycin	50	3.87	4.94	7.02	20.32	8.92	8.77
165	Hydroxyprogesterone caproate	50	3.01	4.04	2.81	5.5	9.8	3.58
166	Gatifloxacin	50	6.72	4.48	2.51	3.27	3.9	2.38
167	Tolbutamide	50	8.48	5.5	3.96	18.18	7.75	2.53
168	Tosufloxacin	50	4.7	4.91	2.02	7.92	7.91	5.22
169	Mebendazole	50	7.83	5.8	6.05	5.47	1.18	2.39
170	Xylazine	50	3.85	3.65	8.51	22.39	3.58	8.69
171	Megestrol	50	4.48	2.8	3.26	12.44	1.26	6.44
172	Mefenamic acid	50	6.23	2.52	6.32	22.73	11.37	6.48
173	17-Methyltestosterone	50	2.59	6.69	8.64	3.86	2.28	4.01
174	Methylprednisolone	50	4.68	4.51	4.3	9.38	5.35	2.81
175	Methylprednisolone acetate	50	5.56	4.26	7.12	4.8	14.2	4.46
176	D-(-)-Norgestrel	50	7.58	2.42	3.9	7.48	6.81	7.94
177	Meclocycline	50	8.85	5.64	5.65	3.9	2.58	8.28
178	Methacycline	50	7.32	3.72	3.48	18.98	0.51	3.89
179	Metronidazole	50	2.31	6.67	3.53	17.38	1.69	8.86
180	Trimethoprim	50	8.62	6.31	5.98	19.43	13.89	8.36
181	Nequinate	50	5.88	7.91	7.61	3.27	9.66	3.11
182	Josamycin	50	4.98	4.63	6.03	21.84	12.13	0.6
183	Crystal violet	50	5.24	8.04	6.07	8.04	2.99	8.54

Number	Compounds	SDL （μg/L）	Intra-day RSD（%）			Inter-day RDD（%）		
			50（μg/L）	100（μg/L）	500（μg/L）	50 （μg/L）	100（μg/L）	500（μg/L）
184	Amantadine	50	2.26	6.18	4.72	15.12	0.75	4.76
185	Rimantadine	50	3.02	6.4	4.32	21.46	7.1	5.46
186	Chlortetracycline	50	7.97	4.48	5.33	3.63	9.17	3.61
187	Metoprolol tartrate	50	8.45	4.11	5.72	17.67	14.24	0.67
188	Carazolol	50	6	8.61	2.24	4.35	11.67	3.77
189	Caffeine	50	3.76	5.84	2.93	8.21	13.79	7.04
190	Carbadox	50	2.13	8.81	7.18	14.35	12.22	0.8
191	Carprofen	50	7.01	3.71	7.66	19.38	8.03	8.67
192	Captopril	50	2.55	2.8	4.17	10.08	14.82	7.72
193	Cambendazole	50	3.09	9.22	3.11	7.25	3.86	9.22
194	Cortisone	50	6.75	4.39	3.35	5.08	11.8	5.27
195	Clarithromycin	50	9.37	6.99	3.12	18.28	6.84	2.45
196	Clonidine	50	8.09	4.64	7.09	5.97	9.65	0.02
197	Clindamycin	50	4.81	8.93	3.95	20.62	11.25	9.19
198	Clinafloxacin	50	8.93	9.37	6.53	21.29	2.33	8.57
199	Clenhexerol	50	5.62	7.46	4.91	6.58	10.94	3.27
200	Clencyclohexerol	50	6.95	8.27	5.05	17.61	0.87	8.72
201	Clenbuterol	50	6.25	8.13	7.37	21.54	7.34	8.14
202	Malachite green oxalate	50	4.24	3.53	6.14	21.26	11.77	1.41
203	Quinoxaline-2-carboxylic acid	50	7.34	3.52	6.96	10.52	1.25	7.43
204	Labetalol	50	4.08	5.14	4.53	20.84	11.48	8.92
205	Ractopamine	50	7.33	3.21	3.89	9.45	13.67	0.44
206	Lorazepam	50	8.02	4.68	7.89	10.64	9.27	5.61
207	Rilmenidine	50	7.51	5.14	3.99	12.59	7.59	7.78
208	Chlordiazepoxide	50	6.51	4.64	5.49	14	13.77	1.27
209	Ritodrine	50	9.27	6.82	5.44	10.83	14.07	8.13
210	Reserpine	50	2.89	2.03	7.62	18.33	14.42	4.86
211	Lincomycin	50	4.61	2.54	7.15	18.77	9.76	0.18
212	Rosiglitazone	50	6.43	3.42	4.18	7.14	1.01	3.22
213	Roxithromycin	50	3.18	5.46	5.82	19.82	1.97	6.72
214	Tetrahydropalmatine	100	-	2.74	4.83	-	10.33	6.56
215	Ronidazole	50	2.84	7.77	4.04	15.85	10.65	6.47
216	Spiramycin	50	9.37	3.27	5.82	4.09	13.51	8.99
217	Lovastatin	50	7.39	4.54	5.69	4.95	13.12	7.83
218	Lovastatin sodium salt	50	4.04	2.27	2.38	4.29	2.97	2.39
219	Roxarsone	100	-	3.84	4.71	-	14.98	7.96
220	Lomefloxacin	50	5.1	4.16	3.05	20.45	2.83	3.9
221	Clobetasol 17-propionate	50	2.71	8.02	6.27	21.9	11.98	7.92
222	Chlorpheniramine maleate	50	8.09	4.77	7.47	21.26	9.41	7.25
223	Clorprenaline	50	7.77	9.2	4.41	7.18	8.9	7.24

Number	Compounds	SDL （μg/L）	Intra-day RSD（%）			Inter-day RDD（%）		
			50（μg/L）	100（μg/L）	500（μg/L）	50 （μg/L）	100（μg/L）	500（μg/L）
224	Chlorpromazine	50	9.35	8.19	9.36	5.14	7.67	3.36
225	Chlormadinone	50	6.83	5.17	3.32	6.79	9.66	9.12
226	5-Chloro-1-methyl-4-nitroimidazole	50	8.05	3.14	4.25	11.96	10.45	2.55
227	Chlormezanone	50	7.87	3.71	3.55	11.69	2.24	0.82
228	Clopidol	100	-	2.76	6.69	-	7.97	5.82
229	Closantel	50	2.4	2.3	7.03	9.82	8.47	0.12
230	Clonazepam	50	8.63	7.7	2.6	15.22	3.19	4.43
231	Cloxacillin	50	5.93	5.27	6.53	14.42	12.4	8.16
232	Marbofloxacin	50	4.63	9.27	7.57	6.36	13.42	3.14
233	Maduramycin ammonium	50	8.24	4.83	3.61	8.36	8.56	4.91
234	Mapenterol	50	3.78	4.44	3.73	6.03	2.37	6.18
235	Mevastatin	100	-	7.35	4.4	-	2.97	3.73
236	Mesterolone	50	6.13	8.19	7.13	7.24	7.26	7.51
237	Melengestrol	50	3.09	7.18	4.29	19.5	13.66	4.09
238	Meloxicam	50	2.24	5.17	5.1	9.61	3.1	3.35
239	Medetomidine	50	6.54	9.08	3.56	15.61	5.68	2.55
240	Mestanolone	50	9.32	5.12	6.56	4.16	7	3.65
241	Methandrostenolone	50	6.44	2.8	7.97	3.25	2.68	8.79
242	Midazolam	50	5.24	5.5	2.29	3.2	8.94	8.61
243	Minocycline	50	7.52	2.47	3.5	5.98	8.57	6.47
244	Meprobamate	100	-	4.84	8.52	-	11.57	2.39
245	Muraglitazar	50	3.76	3.87	4.08	4.53	5.29	6.82
246	Mometasone	50	4.85	9.11	8.37	12.75	13.84	2.58
247	Moxidectin	50	8.83	8.03	6.3	20.93	7.97	8.75
248	Moxifloxacin	50	2.18	5.62	6.66	14.63	11.46	9.03
249	Nadifloxacin	50	2.5	5.79	5.42	5.28	6.8	2.94
250	Nabumetone	50	5.56	8.31	3.6	11.37	8.02	8.85
251	Nalidixic acid	50	8.52	3.2	5.54	21.91	4.67	1.36
252	Nafcillin	50	8.53	3.94	2.65	20.23	1.82	2.85
253	Naproxen	50	5.05	3.89	8.35	15.76	0.38	2.73
254	Nimodipine	50	6.29	2.02	6.14	20.9	14.03	0.35
255	Nitrendipine	50	4.31	3.75	5.25	19.5	14.05	2.4
256	Norfloxacin	50	8.39	8.71	8.53	17.59	9.22	6.9
257	Nortestosterone	50	2.9	4.53	3.16	17.11	13.13	3.24
258	Piperacillin	500	-	-	2.37	-	-	8.18
259	Prazosin	50	3.09	6.66	5.03	18.84	9.06	5.96
260	Pefloxacin	50	4.01	9.19	8.8	12.82	5.65	6.31
261	Penbutolol	50	7.89	4.94	4.89	5.88	9.83	7.74
262	Corticosterone	50	5.9	6.87	3.83	22.66	9.21	3.26
263	Prednicarbate	50	5.28	5.02	7.09	21.91	11.59	0.23

Number	Compounds	SDL（μg/L）	Intra-day RSD（%）			Inter-day RDD（%）		
			50（μg/L）	100（μg/L）	500（μg/L）	50 （μg/L）	100 （μg/L）	500 （μg/L）
264	Prednisone	50	9	8.41	6.45	20.52	9.91	7.37
265	Prednisolone	50	8.16	7.49	3.19	8.55	6.89	5.02
266	Propranolol	50	7.68	5.79	9.18	15.1	8.99	1.13
267	Hydroxy metronidazole	50	4.1	8.41	7.33	18.11	9.49	3.18
268	Hydroxy ipronidazole	50	2.07	9.3	6.07	17.49	0.6	3.12
269	Hydroxy dimetridazole	50	7.85	2.01	9.74	17.37	5.88	9.1
270	Oxymetholone	50	7.47	2.02	5.57	5.67	6.94	3.73
271	Penicillin G potassium	50	3.16	2.11	3.41	21.94	11.54	5.93
272	Penicillin V potassium salt	50	5.06	3.57	4.53	5.7	3.87	8.87
273	Hydrocortisone	50	9.44	8.43	3.1	7.54	8.36	6
274	Hydrocortisone 17-butyrate	50	6.31	6.74	4.03	9.69	4.58	8.98
275	Hydrocortisone 17-valerate	50	6.62	6.87	3.88	16.9	11.98	1.7
276	Fenoterol hydrobromide	50	8.33	3.71	4.32	21.01	11.21	8.59
277	Triamcinolone acetonide	50	7.32	7.82	6.37	20.39	8.34	4.62
278	Triamcinolone	500	-	-	7.47	-	-	8.58
279	Troglitazone	50	7.35	4.71	7.07	12.5	9.99	8.15
280	Desmethyl sibutramine	50	5.92	3.43	7.59	16.63	9.55	3.97
281	Dehydro lovastatin	50	7.97	3.19	8.8	12.12	5.66	1.49
282	Boldenone	50	8.39	5.96	6.34	4.08	0.41	4.82
283	Ethinyl estradiol	500	-	-	4.22	-	-	0.56
284	Quinestrol	50	3.1	8.41	3.89	22.83	11.17	7.28
285	Norethindrone	50	7.65	6.66	6.87	5.75	1.39	8.89
286	Trenbolone	50	5.72	8.65	6.29	12.66	5.29	8.8
287	Repaglinde	50	3.2	2.95	5.92	22.84	5.51	0.93
288	Secnidazole	50	4.43	5.95	4.16	19.72	14.41	3.57
289	Thiabendazole	50	8.43	9.01	7.02	7.53	2.05	8.63
290	Cyproheptadine	50	6.43	2.53	3.7	7.18	2.05	0.67
291	Diminazene	100	-	2.24	7.82	-	7.24	0.98
292	Ketotriclabendazole	50	2.71	5.11	6.21	3.17	13.25	5.34
293	Triclabendazole	50	2.13	2.2	5.2	20.46	0.47	4.74
294	Triclabendazole sulfone	50	3.87	6.74	6.88	9.89	2.38	3.09
295	Triazolam	50	7.95	2.47	2.97	15.8	4.09	5.02
296	Salbutamol	50	4.89	3.57	4.29	11.66	10.88	7.29
297	Sarafloxacin	50	4.66	8.9	7.6	12.73	3.15	1.37
298	Salmeterol	50	8.74	7.63	2.11	20.14	10.21	8.39
299	Sulindac	50	3.47	4.31	3.49	17.64	13.23	8.71
300	Difloxacin	50	8.84	5.28	3.1	10.38	5.7	6.66
301	Amitraz	50	5.11	3.21	5.02	13.41	2.33	6.86
302	Diclofenac acid	50	8.98	4.93	6.78	21.46	11.98	7.62
303	Diclofenac sodium	50	6.73	4.13	4.45	20.26	7.7	1.27

Number	Compounds	SDL (μg/L)	Intra-day RSD（%）			Inter-day RDD（%）		
			50（μg/L)	100（μg/L)	500（μg/L)	50 （μg/L)	100（μg/L)	500（μg/L)
304	Dihydrotestosterone	50	4.66	4.46	4.27	5.73	11.49	3.77
305	Sparfloxacin	50	7.06	6.92	3.94	6.64	6.81	7.62
306	Stanozolol	50	6.59	2.69	8.3	15.53	7.16	2.83
307	Tetracycline	50	4.62	4.54	3.42	7.13	12.33	9.15
308	Tylosin	50	3.09	7.99	7.22	10.2	14.78	5.34
309	Tiamulin	50	6.46	5.75	2.05	7.88	0.16	7.29
310	Terbutaline	50	6.6	6.33	5.81	16.59	4.68	1.78
311	Tilmicosin	50	4.37	4.51	5.28	13.28	6.05	7.96
312	Tenoxicam	50	7.19	2.88	5.5	6.92	3.6	0.96
313	Tinidazole	50	2.86	7.27	5.92	21.36	0.2	6.83
314	Tizanidine	50	3.75	4.19	2.53	8.95	2.47	1.18
315	Ketoprofen	50	6.62	6.48	6.23	5	9.77	3.19
316	Cephalexin	500	-	-	6.69	-	-	3.00
317	Cefotaxime	50	2.84	9.13	8.86	8.99	9.83	3.16
318	Cefaclor	100	-	8.91	2.36	-	1.11	3.74
319	Cefixime	50	6.74	3.32	7.81	11	0.43	2.24
320	Cefquinome	100	-	4	5.54	-	2.59	4.04
321	Cephradine	500	-	-	8.82	-	-	7.44
322	Cephalonium	50	4.69	2.41	8.51	8.32	1.47	1.42
323	Cefamandole	50	7.3	4.85	6.37	16.01	4.91	7.76
324	Cefminox	100	-	2.25	5.34	-	3.37	6.47
325	Cefoperazone	50	4.52	6.37	2.19	3.09	3.42	2.33
326	Cephapirin	50	5.65	4.49	6.41	22.74	0.3	4.85
327	Cefpirome	500	-	-	7.15	-	-	1.34
328	Cefadroxil	100	-	7.53	4.82	-	8.74	6.51
329	Ceftiofur	50	3.74	5.15	9.74	13.62	0.49	7.09
330	Ceftazidime	500	-	-	3.88	-	-	6.68
331	Cefetamet pivoxil	50	7.76	7.92	5.15	15.99	3.73	6.68
332	Cefazolin	50	8	4.46	8.25	10.96	6.35	8.63
333	Oxytetracycline	50	5.4	7.91	4.89	14.22	2.93	2.1
334	Melatonine	50	6.69	4.95	2.08	4.25	11.02	8.19
335	Tolmetin	50	9.14	6.79	8.56	9.35	1.68	8.32
336	Tolfenamic acid	50	5.17	8.74	2.87	3.67	11.13	3.82
337	Dehydroepiandrosterone	50	6.45	6.23	9.26	22.57	4.55	1.21
338	Anhydroerythromycin A	50	4.58	5.59	5.1	9.04	2.2	4.95
339	Desoxycarbadox	50	2.05	7.18	6.78	11.07	12.26	7.37
340	Cortexolone	50	8.06	2.29	6.58	3.61	10.45	0.07
341	Tulobuterol	50	5.42	4.38	3.5	3.29	7.06	2.31
342	Toltrazuril	50	2.56	3.51	2.35	18.03	8.24	6.71
343	Toltrazuril sulfoxide	50	8.41	6.29	7.04	10.02	8.69	7.41

Number	Compounds	SDL（μg/L）	Intra-day RSD（%）			Inter-day RDD（%）		
			50（μg/L）	100（μg/L）	500（μg/L）	50 （μg/L）	100（μg/L）	500（μg/L）
344	Vildagliptin	50	4.81	6.67	7.1	6.02	3.8	1.53
345	Virginiamycin M1	50	2.14	2.76	4.68	10.07	0.94	4.22
346	Venlafaxine	50	8.11	7.85	8.57	8.01	6.06	1.63
347	Sildenafil	50	4.07	4.63	8.61	20.6	8.2	2.33
348	Cimaterol	50	2.17	6.61	5.29	20.57	11.33	6.4
349	Cinoxacin	50	2.64	2.95	6.31	14.9	3.25	5.83
350	Sitagliptin	50	7.53	8.43	8.61	21.02	8.27	8.54
351	Nifedipine	50	5.55	7.02	6.86	22.42	9.14	4.47
352	Nitrazepam	50	2.2	5.55	4.46	7.7	13.89	1.33
353	Simvastatin	50	5.59	4.53	4.56	20.29	13.96	2.32
354	Androsterone	50	5.92	7.5	8.46	8.13	6.29	3.69
355	Bromchlorbuterol	50	6.41	2.18	7.22	17.84	8.79	7.68
356	Ofloxacin	50	6.93	8.4	2.59	21.98	12.15	4.54
357	Enoxacin	50	2.23	2.12	8.5	23.2	4.79	0.24
358	Eprinomectin	50	8.28	5.72	7.87	13.46	13.01	1.62
359	Megestrol acetate	50	2.12	8.08	8.87	6.5	13.01	4.17
360	Mequindox	50	6.01	9.5	5.97	5.15	0.92	0.63
361	Ipronidazole	50	2.77	3.71	6.36	7.57	11.28	6.93
362	Isochlortetracycline	100	-	6.34	2.54	-	6.75	4.51
363	Indomethacin	50	7.24	4.54	2.47	10.1	7.21	4.58
364	Leucomalachite green	50	6.62	6.35	6.01	18.29	3.1	5.7
365	Leucocrystal violet	50	7.69	3.2	6.22	18.28	8.69	0.13
366	Indoprofen	50	4.75	2.28	2.65	9.1	7.08	8.16
367	Progesterone	50	4.23	7.32	7.25	22.39	9.66	4.56
368	Pregnenolone	50	3.02	5.29	4.24	19.17	12.47	5.62
369	Zaleplon	50	2.67	5.92	4.09	8.74	7.84	8.42
370	Oleandomycin	50	5.21	7.08	6.83	20.2	7.62	3.36
371	Levamisole	50	6.18	7.29	7.36	4.7	0.72	2.09
372	Levofloxacine	50	6.03	8.71	8.71	21.72	11.1	6.2
373	Zolpidem	50	5.58	6.03	7.22	12.62	3.27	0.37
374	3,5-Dinitrobenzamide	100	-	6	3.28	-	3.33	1.93
375	ez-Diethylstilbestrol	100	-	6.4	4.75	-	0.27	9.71
376	Analgin	100	-	2.69	7.76	-	3.54	7.77
377	Barbital	100	-	7.19	5.89	-	2.89	8.69
378	Phenobarbital	500	-	-	2.71	-	-	9.28
379	Ibuprofen	500	-	-	4.14	-	-	4.63
380	Dapagliflozin	100	-	7.25	2.21	-	7.58	6.71
381	Diclazuril	500	-	-	4.62	-	-	5.89
382	Diflunisal	500	-	-	2.34	-	-	6.93
383	Sodium nifurstylenate	100	-	5.26	4.68	-	3.65	4.39

Number	Compounds	SDL （μg/L）	Intra-day RSD（%）			Inter-day RDD（%）		
			50（μg/L)	100（μg/L)	500（μg/L)	50 （μg/L)	100（μg/L)	500（μg/L)
384	Furosemide	100	-	6.54	8.98	-	6.25	3.6
385	Florfenicol	100	-	3.45	5.39	-	6.04	5.83
386	Flurbiprofen	100	-	9.31	2.59	-	0.14	1.9
387	Ciglitazone	100	-	3.88	2.66	-	3.9	5.95
388	Hexestrol	500	-	-	3.48	-	-	3.14
389	Diethylstilbestrol	100	-	2.38	4.75	-	12.27	2.74
390	Narasin	500	-	-	5.84	-	-	0.71
391	Lasalocid	100	-	2.92	5.44	-	12.33	5.97
392	Monensin	500	-	-	2.67	-	-	2.9
393	Nicarbazin	100	-	8.89	3.02	-	12.68	4.56
394	Hydrochlorothiazide	100	-	8.58	7.89	-	3.09	6.53
395	Sulbactam	100	-	2.33	6.67	-	2.75	5.08
396	Dienestrol	100	-	4.89	2.44	-	11.81	0.2
397	Secobarbital	100	-	3.22	7.72	-	13.98	9.86
398	Ponazuril	100	-	7.82	3.97	-	6.64	6.45
399	Nitroxynil	100	-	6.26	5.96	-	8.66	0.54
400	Salinomycin	100	-	4.59	6.52	-	4.36	4.83
401	Amobarbital	100	-	9.22	6	-	6.01	4.26
402	4,4′-Dinitrocarbanilide	50	6.49	4.14	5.05	3.25	6.11	6.13
403	Propetamphos	50	2.84	3.47	2.62	20.08	7.78	7.5
404	Fenthion	50	4.68	5.74	3.95	21.27	9.91	8.61
405	Ricinine	50	6.15	8.74	5.9	15.72	0.96	5.29
406	Trichlorfon	50	2.73	3.27	7.98	8.2	10.35	9.08
407	Dichlorvos	50	2.39	2.01	2.65	20.08	5.39	7.46
408	Fluazuron	50	7.39	4.73	3.8	6.89	12.76	2.35
409	Diazinon	50	4.36	7.56	4.6	9.42	10.85	5.49
410	Malathion	50	2.79	4.11	4.05	4.52	9.4	9.19
411	Phoxim	100	-	8.39	8.82	-	7.4	3.19
412	Ethopabate	50	3.95	8.43	8.07	16.83	13.91	6.85
413	Salicylic acid	100	-	5.24	2.62	-	2.74	7.92
414	Sodium pentachlorophenoxide	100	-	5.88	6.52	-	6.74	6.74
415	α-Zearalenol	100	-	7.95	4.18	-	3.33	1.93
416	β-Zearalanol	100	-	3.91	8.45	-	0.27	9.71
417	β-Zearalenol	100	-	4.85	4.36	-	3.25	1.69
418	Zearalanol	100	-	4.92	2.35	-	0.78	7.7
419	Zearalanone	100	-	7.89	2.74	-	5.01	9.31
420	Zearalenone	100	-	2.77	2.53	-	2.47	8.51

Table S5. Stability test results of standard reserve solution and mixed standard intermediate solution of 420 risk substances

Number	Compounds	-18℃			+4℃			+18℃			-18℃
		1 month（%）	2 months（%）	6 months（%）	1 month（%）	2 months（%）	6 months（%）	1 month（%）	2 months（%）	6 months（%）	1 week（%）
1	2-Aminoflubendazole	97.93	98.69	98.04	100.69	99.02	99.65	101.53	99.84	99.45	99.46
2	2-Methyl-5-nitroimidazole	100.38	98.65	98.08	99.39	99.66	100.58	99.34	102.64	98.03	98.77
3	2-NP-SEM	97.65	98.27	97.41	99.52	95.67	96.31	99.51	98.73	100.72	99.93
4	3-Methyl-quinoxaline-2-carboxylic acid	96	103.55	99.93	99.02	101.41	98.47	99.31	100.45	100.3	101.11
5	Desacetyl cefotaxime	97.04	103.88	99.18	101.72	100.76	101.13	102.95	97.81	96.41	100.63
6	4-Aminoantipyrine	100.09	98.25	100.06	97.43	97.83	96.42	101.39	97.61	102.04	100.88
7	4-Formylaminoantipyrine	98.14	98.19	95.05	99.78	98.61	95.46	99.11	102.54	100.55	98.64
8	4-Isopropylaminoantipyrine	95.68	100.38	97.76	97.84	100.4	99.69	100.38	102.62	103.08	99.78
9	5-Hydroxymebendazole	98.26	103.96	101.08	101.38	103.36	103.83	103.74	100.99	101.04	99.2
10	5-Hydroxythiabendazole	102.05	99.91	100.15	101.06	102.54	100.99	99.8	101.24	100.6	102.25
11	17α-Estradiol	98.27	99.77	98.07	99.32	102.37	102.06	102	102.37	102.26	98.93
12	17α-Hydroxyprogesterone	98.08	98.15	102.94	99.3	100.24	98.68	101.67	102.47	100.47	101.56
13	(22R)-Budesonide	98.91	99.42	100.65	102.62	100.53	101.17	103.36	100.1	102.3	99.37
14	α-Trenbolone	99.65	96.86	99.21	98.3	99.76	98.36	101.48	99.76	100.71	97.95
15	N-Didesmethyl Sibutramine	100.42	100.14	101.6	100.57	103.51	100.99	100.48	102.18	102.93	100.61
16	Albendazole sulfone	102.69	98.61	97.9	98.86	102.26	98.78	99.66	98.54	97.34	98.21
18	Albendazole sulfoxide	102.7	102.23	100.08	97.35	97.57	97.45	103.01	99.81	103.07	100.72
18	Albendazolesulf oxide	98	98.35	100.26	100.55	97.45	96.84	102.19	98.33	98.73	100.16
19	Albendazole-2-aminosulfone	98.81	99.7	103.63	99.8	99.98	99.88	99.25	99.74	101.53	98.94
20	Aklomide	98.45	101.64	98.39	101.74	98.7	98.61	103.1	97.62	100.49	102.08
21	Alclomethasone dipropionate	99.65	99.1	100.76	101.01	98.07	98.94	103.51	99.5	97.95	102.03
22	Alprazolam	99	102.44	97.17	98.39	100.21	97.21	100.11	96.05	95.81	100.05
23	Atenolol	99.12	102.51	100.63	96.4	97.85	101.86	100.36	101.06	101.08	98.34
24	Atropine	96.19	97.63	101.99	99.45	98.17	98.27	99.08	98.93	102.47	102.36
25	Estazolam	100.12	97.04	96.81	96.48	98.15	96.38	103.61	97.33	101.16	101.65
26	Methaqualone	100.73	96.17	100.29	102.39	102.18	100.31	100.92	95.71	98.11	100.23
27	Antipyrine	96.97	101.98	96.14	97.9	98.06	101.13	101.46	98.42	98.84	97.97
28	Amcinonide	96.38	102.1	101.23	95.93	100.9	101.36	101.3	97.57	100.2	98.9
29	Aminophylline	104.36	97.9	103.97	97.01	97.27	98.27	99.01	99.03	100.44	100.88
30	Mebendazole amine	95.15	102.05	101.92	98.63	97.25	98.24	102.99	103.67	101.79	97.28
31	Amino tadalafil	99.66	98.11	98.05	99.6	97.81	100.41	101.22	98.3	103.55	100.72
32	Tranexamic acid	103.53	98.2	99.95	100.36	99.66	97.65	101	103.89	103.02	98.28
33	Amlodipine	97.61	99.26	98.08	99.61	96.72	101.32	99.57	97.83	100.5	99.66
34	Orbifloxacin	98.44	98.63	99.32	98.06	103.2	103.44	100.24	102.22	98.37	100.36
35	Oxfendazole	100.48	97.22	99.37	96.53	98.92	95.74	101.63	101.84	98.85	100.08
36	Olaquindox	96.17	101.14	97.29	95.42	96.36	97.66	101.58	99.37	96.94	100.96

Number	Compounds	-18℃			+4℃			+18℃			-18℃
		1 month （%）	2 months （%）	6 months （%）	1 month （%）	2 months （%）	6 months （%）	1 month （%）	2 months （%）	6 months （%）	1 week （%）
37	Oxazepam	98.86	96.58	98.01	97.54	98.92	98.21	99.47	99.28	100.2	103.26
38	Oseltamivir	98.24	103.12	103.22	100.86	102.45	102.52	99.52	97.41	97.35	97.93
39	Ornidazole	99.19	98.42	97.75	99.85	96.14	96.64	101.25	99.23	100.65	99.74
40	Baclofen	97.72	102.7	95.09	97.68	99.46	97.5	100.65	100.03	99.05	99.15
41	Beclomethasone	100.76	96.83	101.57	99.94	101.77	99.61	100.23	101.59	97.24	99.38
42	Beclomethasone dipropionate	98.67	98.54	95.24	98.27	100.4	101.8	99.81	101.05	101.64	98.55
43	Betamethasone	98.21	99.95	98.6	99.02	99.95	99.47	100.22	100.12	103.63	98.81
44	Betamethasone dipropionate	99.28	97.48	100.82	97.32	100	97.8	102.71	101.5	102.48	97.1
45	Betamethasone 17-valerate	96.98	97.84	97.25	98.05	99.77	99.47	99.97	98.83	99.14	99.52
46	Clobetasone butyrate	99.35	99.69	96.85	99.33	99.69	96.89	101.29	103.19	103.2	100.6
47	Nadrolone phenylpropionate	97.63	100.87	97.14	97.99	97.85	99.72	99.76	98.34	101.96	98.36
48	Benzimidazole	100.18	101.16	101.12	102.17	100.68	99.36	102.06	99.21	100.88	102.54
49	Sulfabenzamide	97	96.17	97.9	96.75	99.87	101.89	99.02	99.21	96.59	100.27
50	Febantel	100.85	99.91	100.66	99.77	98.04	97	101.62	96.5	101.41	102.85
51	Azlocillin	102.19	97.62	101.32	102.77	100.29	102.51	102.47	101.22	96.17	99.89
52	5-Nitrobenzimidazole	98.6	102.86	97.94	98.22	102.97	99.72	101.4	97.57	99.43	100.77
53	Isoxsuprine	101.76	103.22	98.11	97.03	98.74	100.79	100.55	99.94	102.25	100.66
54	Phenylethanolamine A	97.41	101.03	98.49	99.49	100.88	101.07	99.29	95.63	97.63	98.25
55	Phenformin	99.28	100.47	102.25	97.94	97.27	98.58	101.51	103.27	99.61	97.23
56	Oxacillin	98.36	98.74	98.07	98.84	99.86	102.67	103.75	96.32	99.82	100.17
57	Pirlimycin	95.3	103.65	100.02	97.24	102.97	97.26	100.67	96.7	100.51	99.72
58	Piroxicam	98.41	96.42	97.02	97.91	95.15	98.31	101.49	99.17	102.09	99.27
59	Epitestosterone	97.93	97.57	96.91	102.27	100.82	99.14	102.91	98.87	102.46	98.43
60	Testosterone propionate	98.1	99.78	99.38	97.86	97.87	101.38	101.28	96.53	99.63	99.39
61	Nandrolone propionate	99.39	99.85	100.3	98.14	101.93	99.21	99.4	96.14	101.33	99.72
62	Oxibendazole	98.8	100.19	99.64	97.03	101.42	102.72	100.27	96.64	97.12	101.93
63	Budesonide	98.68	98.61	103.18	97.19	96.4	103.03	99.82	101.1	100.76	102.06
64	Halofuginone	97.77	97.13	97.99	98.44	96.78	98.41	103.08	99.79	96.55	99.53
65	Estradiol	99.86	98.42	97.98	100.31	96.6	99.47	103.02	98.9	103.16	100.53
66	Estrone	99.43	100.1	99.48	96.11	99.68	101.82	102.82	102.67	98.31	101.16
67	N-Acetylsulfamethoxazole	96.79	102.92	99.59	98.05	95.57	102.25	101.31	101.55	103.29	99.32
68	Aceclofenac	96.01	99.57	99.27	98.93	101.85	96.55	99.33	101.72	100.55	99.03
69	Betamethasone 21-acetate	98.96	98.35	98.73	100.73	100.75	98.31	103.22	99.54	101.73	99.99
70	Dexamethasone 21-acetate	98.79	98.94	98.29	99.27	103.1	100.25	99.35	97.67	101.18	96.7
71	Fluorometholone 17-Acetate	97.51	102.34	97.35	98.73	98.25	98.77	101.49	98.56	96.3	98.75
72	Fludrocortisone 21-acetate	97.32	95.95	97.03	99.75	97.41	96.55	102.12	95.43	97.54	97.29
73	Flugestone acetate	101.31	98.73	102.93	98.45	98.46	95.25	101.17	100.98	102.32	97.59
74	Cyproterone acetate	97.8	102.17	102.08	102.46	100.03	100.99	103.64	101.13	99.14	99.92

Number	Compounds	-18℃			+4℃			+18℃			-18℃
		1 month （%）	2 months （%）	6 months （%）	1 month （%）	2 months （%）	6 months （%）	1 month （%）	2 months （%）	6 months （%）	1 week （%）
75	Medroxyprogesterone 17-acetate	100	96.06	95.88	99.36	100.02	96.31	101.66	103.19	97.06	102.17
76	Cortisone 21-acetate	99.21	98.25	100.94	100.83	100.81	97.26	99.29	98.46	96.05	96.83
77	Chlormadinone acetate	97.56	97.46	98.12	98.79	104.55	102.89	99.54	103.72	101.53	101.98
78	Melengestrol acetate	96.78	96.99	96.4	101.35	101.13	99.36	103.29	98.89	101.09	101.41
79	Prednisone 21-acetate	102.97	98.92	102.87	100	97.16	98.27	101.72	100.08	99.69	102.96
80	Prednisolone 21-acetate	100.24	96.71	103.37	99.88	97.87	100.81	99.3	100.23	99.11	100.48
81	17α-Hydroxyprogesterone acetate	98.29	97.58	98.12	100.14	97.06	103.99	101.21	97.61	103.9	100.65
82	Hydrocortisone acetate	97.83	95.92	97.58	98.07	100.88	98.87	103.92	99.33	97.26	98.24
83	Triamcinolone acetonide acetate	96.16	97.44	98.12	100.6	101.12	101.52	101.87	100.16	98.62	96.98
84	Triamcinolone diacetate	99.13	102.06	98.74	98.18	101.54	101.69	100.89	98.42	99.45	100.69
85	Norethisterone acetate	102.85	95.95	103.83	99.98	98.83	100.05	103.05	101.9	101.06	100.37
86	Danofloxacin	96.36	98.79	97.24	97.15	98.97	98.83	101.51	97.78	97.2	97.62
87	Azaperone	99.12	97.49	98.12	99.32	98.18	99.83	102.58	101.51	103.8	100.66
88	Deflazacort	100.06	97.42	99.88	97.45	100.24	97.59	101.22	103.59	101.94	103.11
89	Dexamethasone	96.32	100.45	97.45	99.97	102.71	103.57	99.69	100.87	103.62	101.85
90	Diazepam	99.9	102.93	102.13	103.31	99.43	102.06	102.33	99.33	98.46	101
91	Dicyclanil	99.06	101.95	101.73	99.68	99.47	102.37	100.18	98.76	102.07	98.55
92	Buquinolate	101.33	101.93	101.1	99.9	101.08	102.64	100.6	100.38	97.86	100.47
93	Sultamicillin tosilate	97.05	97.89	98.66	101.37	102.69	98	101.99	100.85	98.94	97.85
94	Acetaminophen	99.05	98.39	96.37	98.47	95.47	96.69	100.03	95.53	98.08	99.34
95	Doramectin	99.21	98.84	96.41	98.46	99.81	97.82	102.79	103.85	102.75	99.16
96	Doxycycline	96.94	101.3	101.33	100.12	97.43	102.9	101.55	99.54	99.02	100.99
97	Oxolinic acid	96.73	102.2	98.8	100.88	96.46	98.41	99.47	97.96	96.11	101.53
98	Enrofloxacin	99.1	96.24	97.45	98.82	99.07	101.89	103.82	98.96	96.67	98.94
99	Diflorasone diacetate	102.9	98.99	97.14	101.25	98.53	100.51	100.42	103.74	96.23	100.13
100	Dimetridazole	103.54	102.88	103.57	99.07	102.25	95.67	103.93	103.46	101.19	100.05
101	Dinitolmide	100.47	99.59	101.31	97.11	99.53	96.48	102.69	97.36	96.3	97.83
102	Dioxopromethazine	100.72	98.76	100.64	101.43	97.66	101.38	101.18	103.36	103.23	99.12
103	Felodipine	99.17	99.16	99.37	100.38	97.98	102.76	102.06	100.69	101.83	102.49
104	Phenacetin	96.11	98.51	97.68	102	101.76	99.62	99.8	98.25	103.08	100.95
105	Fenbendazole	97.76	97.46	97.07	100.7	102.22	98.76	100.11	101.76	100.05	97.41
106	Fenbendazole sulfone	98.44	102.35	100.18	99.88	97.75	96.84	98.93	100.95	98.12	99.98
107	Fenfluramine	98.6	99.55	101.18	101.31	98.82	97.85	101.27	98.42	99.41	99.77
108	Phenolphthalein	96.67	102.26	97.13	96.89	95.11	100.11	99.52	101.94	103.34	100.04
109	Rimsulfuron	98.17	102.01	99.35	102.5	99.15	98.54	101.9	98.81	100.36	98.12
110	Furaltadone	98.65	95.98	95.91	102.43	103.07	99.56	100.91	101.72	100.68	97.94

Number	Compounds	-18℃			+4℃			+18℃			-18℃
		1 month (%)	2 months (%)	6 months (%)	1 month (%)	2 months (%)	6 months (%)	1 month (%)	2 months (%)	6 months (%)	1 week (%)
111	2-NP-AMOZ	100.03	104.11	102.11	100.05	98.37	102.02	100.08	100.29	100.44	98.89
112	Nitrofurantoin	96.32	97.19	98.02	101.35	97.55	97.57	103.85	97.84	97.54	98.09
113	2-NP-AHD	98.36	102.85	98.51	101.95	100.47	97.54	102.5	100.6	100.13	99.14
114	Furazolidone	102.59	100.22	99.19	100.24	103.24	100.5	99.24	95.24	97.79	97.11
115	2-NP-AOZ	103.13	102.77	99.81	101.56	103.76	98.96	100.63	96.72	100.16	99.14
116	Flubendazole	100.61	98.11	99.39	99.26	96.86	97.51	100.94	103.74	98.36	98.05
117	Flufenamic acid	98.55	97.11	96.37	99.25	97.31	96.41	99.76	96.24	97.43	100.73
118	Flumequin	97.38	98.28	96.07	100.3	98.12	102.93	100.28	98.66	101.1	99.07
119	Fleroxacin	98.08	98.72	99.99	100.07	100.18	98.98	101.38	102.96	102.7	97.4
120	Flucloxacillin	98.05	98.57	96.41	102.03	96.85	98.2	100.63	98.6	100.45	98.99
121	Fluoromethalone	100.58	99.43	100.11	103.19	102.9	102.91	103.3	99.73	102.32	99.75
122	Flumethasone	98.66	97.41	98.67	97.08	96.53	96.26	99.52	100.41	98.28	102.59
123	Flunixin	101.62	96.53	101.65	100.57	100.57	99.36	99.53	98.96	101.17	96.56
124	Haloperidol	101.43	97.98	97	98.06	97.31	97.06	99.94	99.17	98.55	99.87
125	Fluprednisolone	99.12	99.05	98	99.22	96.09	98.99	102.78	102.36	103.28	98.43
126	Fluocinolone acetonide	96.5	101.91	102.2	100.27	100.72	100.55	102.76	97.64	98.93	102.52
127	Fluocinonide	100.14	101.88	101.28	99.6	97.65	99.02	99.46	97.09	102.8	98.55
128	Fludroxycortide	99.43	96.71	97.09	102.46	96.48	102.76	101.3	100.6	99.26	99.78
129	Fluticasone propionate	103.78	98.53	100.57	99.03	98.27	103.58	103.25	102.81	97.98	100.99
130	Formoterol	98.74	99.44	100.24	100.11	95.81	99.49	101.79	103.02	102.08	101.41
131	Testosterone	100.62	99.46	97.68	96.44	100.61	98.08	101.42	95.18	97.2	101.07
132	Glibenclamide	98.52	95.99	97.61	97	97.32	100.48	102.88	99.11	98.34	102.31
133	Glipizide	96.41	99.82	100.87	98.29	101.2	97.61	101.14	98.11	102.8	100.31
134	Glibornuride	96.27	97.27	99.72	99.57	101.37	102.36	99.07	102	101.55	98.04
135	Gliquidone	98.51	98.81	98.98	99.13	96.16	100.1	103.18	98.61	102.55	100.88
136	Glimepiride	99.9	101.26	98.8	99.68	100.95	101.37	103.09	100.61	101.06	99.02
137	Gliclazide	97.89	98.64	100.28	97.65	100.86	97.23	102.99	96.32	99.6	102.29
138	Guanfacine	97.17	101.35	100.79	101.99	102.3	102.44	102.71	98.06	101.82	99.85
139	Decoquinate	99.38	100	99.98	100.37	101.72	102.61	101.72	97.01	100.34	100.23
140	Halcinonide	99.27	99.92	101.56	98.58	99.94	98.72	103.9	100.85	102.85	97.92
141	Erythromycin	97.24	98.61	99.22	99.13	103.37	102.35	102.12	102.67	99.03	99.26
142	Ciprofloxacin	98.69	102.2	100.53	99.68	100.19	103.3	100.6	98.63	96.7	97.67
143	Sulfaphenazole	100.8	99.31	99.68	98.51	100.42	95.46	100.95	100.61	103.65	99.06
144	Sulfapyridine	103.5	102.26	102.74	99.1	97.26	101.65	102.15	98.19	101.42	103.27
145	Sulfapyrazole	97.2	99.04	99.67	98.85	100.53	98.83	98.95	100.87	101.65	96.55
146	Sulfacetamide	102.37	97.52	101.02	98.42	102.77	102.61	102.92	101.87	103.74	102.57
147	Sulfameter	98.32	96.63	98.16	100.7	95.57	100.81	101.61	97.5	101.19	99.88
148	Sulfamoxole	101.58	101.49	100.36	101.37	100.4	98.7	100.34	103.88	99.43	101.27
149	Sulfamethazine	98.52	97.33	98.2	100.39	99.07	103.75	102.17	103.84	100.12	101.38

Number	Compounds	-18℃			+4℃			+18℃			-18℃
		1 month （%）	2 months （%）	6 months （%）	1 month （%）	2 months （%）	6 months （%）	1 month （%）	2 months （%）	6 months （%）	1 week （%）
150	Sulfisoxazole	96.32	102.76	96.19	99.52	101.24	96.83	103.66	99.27	102.91	97.97
151	Sulfisomidine	98.17	100.02	102.66	102.47	95.87	103.13	103.33	96.41	96.65	99.53
152	Sulfamerazine	96.71	98.47	97.4	100.85	104.21	102.96	102.4	97.46	98.37	102.03
153	Sulfamethoxazole	101.36	97.94	100.33	98.24	103.32	103.62	101.54	100.03	98.4	102.42
154	Sulfamethizole	99.1	98.27	100.47	98.95	95.27	96.56	99.01	101.96	103.44	100.09
155	Sulfadimethoxine	98.8	99.72	96.83	99.76	97.29	101.25	102.29	97.45	101.1	101.26
156	Sulfamonomethoxine	97.2	98.51	100.7	98.6	99.01	100.69	102.65	101.9	103.63	100.68
157	Sulfaquinoxaline	99.24	96.08	100.8	101.86	100.99	98.55	103.47	100.25	98.14	98.73
158	Sulfadoxine	98.15	97.34	96.66	101.31	98.85	102.5	100.04	97.75	98.65	98.83
159	Sulfachloropyridazine	98.03	102.39	103.15	98.06	99.99	101.59	99.74	100.52	103.8	99.19
160	Sulfadiazine	97.35	99.04	98.99	98.36	99.38	100	99.5	98.15	97.22	99.35
161	Sulfathiazole	99.44	97.07	98.92	100.01	101.85	103.57	103.61	100.36	103.49	99.04
162	Sulfanitran	98.11	102.21	99.74	102.68	100.76	102.31	103.9	102.39	99.65	98.27
163	Gemifioxacin	101.64	99.22	97.67	98.12	100.94	97.46	103.24	99.78	101.03	100.82
164	Kitasamycin	98.47	97.04	100.23	103.08	101.95	101.74	101.38	98.68	103.65	99.71
165	Hydroxyprogesterone caproate	99.62	97.84	98.94	98	99.1	98.95	101.13	97.64	102.76	102.46
166	Gatifloxacin	98.44	96.63	96.27	99.08	99.8	95.64	100.24	98.54	102.79	101.04
167	Tolbutamide	99.2	99.57	97.85	97.72	98.33	97.72	102.24	103.8	97.3	99
168	Tosufloxacin	95.87	97.16	98	100.35	99.48	97.21	103.24	99.86	100.18	100.84
169	Mebendazole	98	98.49	96.06	103.61	95.68	95.36	103.8	97.58	99.5	101.89
170	Xylazine	96.4	100.9	100.77	100.93	96.23	101.56	101.2	98.65	97.24	101.37
171	Megestrol	99.52	98.5	98.15	102.4	95.6	96.81	101.97	98.12	96.78	101.39
172	Mefenamic acid	98.05	99.66	98.92	101.26	99.17	99.13	102.06	100.07	97.15	100.53
173	17-Methyltestosterone	97.03	99.64	96.79	99.01	99.2	99.86	102.9	99.63	103.15	102.01
174	Methylprednisolone	98.87	97.14	101.53	99.49	99.01	102.6	103.18	101.63	97.48	99.1
175	Methylprednisolone acetate	98.38	99.04	100.89	98.54	100.01	100.06	103.27	99.24	101.82	101.86
176	D-(-)-Norgestrel	97.23	99.31	100.36	97.46	98.54	97.01	102.31	103.9	97.8	99.8
177	Meclocycline	99.64	99.3	101.2	98.96	101.05	99.87	102.09	100.42	100.8	99.12
178	Methacycline	96.06	99.91	96.35	97.03	100.9	100.96	101.82	99.15	101.57	101.87
179	Metronidazole	97.8	98.72	98.94	101.9	99.31	99.4	102.28	99.89	100.77	99.73
180	Trimethoprim	99.19	100.9	98.65	96.92	99.94	100.79	99.93	98.99	98.81	98.71
181	Nequinate	99.16	97.07	97.45	97.07	99.88	102.71	101.2	102.57	98.13	101.05
182	Josamycin	99.15	98.24	99.03	100.66	102.3	101.38	103.9	96.52	102.89	100.29
183	Crystal violet	95.46	101.16	100.39	102.68	100.56	99.4	100.36	98.57	96.71	101.8
184	Amantadine	99.81	98.93	97.92	98.74	101.73	102.61	103.36	103.41	103.5	99.17
185	Rimantadine	101.42	97.16	101.63	97.46	95.17	97.9	102.7	97.47	96.08	99.61
186	Chlortetracycline	99.29	99.66	99.28	100.85	104.28	101.09	103.98	96.1	103.89	101.06
187	Metoprolol tartrate	99.21	103.93	99.91	97.9	100.81	102.86	101.69	96.32	102.23	99.51

Number	Compounds	-18℃			+4℃			+18℃			-18℃
		1 month （%）	2 months （%）	6 months （%）	1 month （%）	2 months （%）	6 months （%）	1 month （%）	2 months （%）	6 months （%）	1 week （%）
188	Carazolol	98.14	97.75	101.77	99.87	98.52	101.28	98.98	103.84	99.86	98.6
189	Caffeine	99.78	95.77	97.32	103.03	97.34	100.03	101.55	102.67	99.62	103.05
190	Carbadox	97.71	102.56	99.94	98.91	96.57	99.44	103.74	101.86	99.8	100.66
191	Carprofen	98.26	98.16	103.18	98.89	100.03	98.15	101.59	99.37	99.5	99.14
192	Captopril	98.7	97.44	99.47	101.17	104.64	103.69	99.02	103.67	96.39	97.31
193	Cambendazole	99.16	99.53	97.08	103.28	98.55	96.14	100.05	99.63	99.9	100.23
194	Cortisone	101.99	98.58	99.84	99.26	100.31	98.3	99.47	97.98	102.43	98.97
195	Clarithromycin	101.49	97.36	101.65	103.4	100.31	98.96	99.68	103.26	97.6	101.52
196	Clonidine	98.76	98.51	103.52	102.6	100.95	97.9	99.56	95.16	98.55	100.38
197	Clindamycin	95.16	98.14	98.51	101.14	99.12	100.44	99.32	96.42	98.09	102.68
198	Clinafloxacin	102.46	96.53	102.44	101.27	98.29	103.13	100.56	97.66	102.14	101.9
199	Clenhexerol	96.63	99.3	100.49	99.13	102.92	99.69	102.5	100.15	99.04	100.69
200	Clencyclohexerol	99.07	99.86	100.14	98.17	100.18	103.53	100.05	99.24	98.27	101.48
201	Clenbuterol	101.64	99.11	99.45	97.81	98.85	97.37	102.72	101.53	98.6	97.92
202	Malachite green oxalate	99.54	102.6	99.3	100.46	99.13	98.78	103.88	96.87	103.62	100.54
203	Quinoxaline-2-carboxylic acid	98.69	98.59	99.54	102.3	99.65	101.72	102.12	96.73	101.11	103.87
204	Labetalol	102.46	98.74	97.54	98.99	98.53	101.1	100.23	101.22	98.47	99.72
205	Ractopamine	96.21	100.05	101.91	98.41	99.93	102.39	103.15	95.35	97.32	99.53
206	Lorazepam	96.04	100.54	103.63	99.83	98.71	103.9	99.33	102.06	99.29	98.27
207	Rilmenidine	102.21	98.68	98.4	99.49	101.47	100.82	100.62	99.15	103.57	100.21
208	Chlordiazepoxide	99.21	102.07	99.02	101.65	100.56	102.66	101.32	96.43	97.45	98.6
209	Ritodrine	99.49	102.98	95.62	97.61	95.96	103.2	100	101.25	99.75	98.45
210	Reserpine	98.61	100.63	99.41	100.57	97.62	98.63	100.77	103.02	102.35	100.58
211	Lincomycin	98.7	100.91	102.37	103.68	98.79	103.84	101.18	101.44	103.8	99.65
212	Rosiglitazone	97.66	102.11	103.94	101.41	103.58	99.11	101.77	98.29	97.35	98.71
213	Roxithromycin	96.1	96.82	103.43	103.96	96.81	102.1	99.09	101.19	101.79	97.57
214	Tetrahydropalmatine	98.06	101.07	95.7	98.1	98.21	99.17	100.82	95.46	98.75	98.1
215	Ronidazole	99.25	99.96	99.09	104.31	95.46	100.39	99.34	97.36	103.69	100.21
216	Spiramycin	99.45	97.39	97.36	99.45	100.4	99.74	101.68	101.32	101.12	100.32
217	Lovastatin	99.77	97.87	97.16	99.93	99.93	99.06	103.47	96.6	100.23	98.11
218	Lovastatin sodium salt	99.39	96.4	99.22	98.75	99.45	99.93	103.73	95.52	98.54	102.53
219	Roxarsone	97.34	98.26	95.36	102.75	97.79	103.53	99.86	101.77	99.24	100.52
220	Lomefloxacin	99.33	97.31	99.24	99.98	97.49	102.73	102.38	97.83	100.89	101.35
221	Clobetasol 17-propionate	99.68	96.16	98.65	99.91	102.06	97.95	99.41	98.6	103.71	97.76
222	Chlorpheniramine maleate	97.36	99.27	101.46	100.48	99.19	99.11	102.85	100.39	100.33	101.86
223	Clorprenaline	102.9	97.98	99.52	98.84	97.88	101.22	102.35	102.78	97.73	98.81
224	Chlorpromazine	102.35	97.65	100.23	102.84	99.56	98.48	100.88	102.44	99.89	102.24
225	Chlormadinone	98.96	97.34	99.46	100.63	101.78	98.94	101.92	102.22	100.9	99.76

Number	Compounds	-18℃			+4℃			+18℃			-18℃
		1 month（%）	2 months（%）	6 months（%）	1 month（%）	2 months（%）	6 months（%）	1 month（%）	2 months（%）	6 months（%）	1 week（%）
226	5-Chloro-1-methyl-4-nitroimi dazole	98.01	103.29	100.67	99.79	97.13	96.53	101.13	99.64	98.92	98.48
227	Chlormezanone	97.86	101.98	96.7	102.54	103.92	99.74	101.23	99.45	100.57	98.23
228	Clopidol	99.36	97.56	97.11	100.38	104.24	99.99	100.59	95.39	102.23	98.56
229	Closantel	102.68	101.86	103.15	99.48	101.31	98.02	99.35	100.19	97.95	101.48
230	Clonazepam	100.15	98.21	99.61	95.01	97.35	95.84	99.87	100.58	103.34	98.05
231	Cloxacillin	102.91	99.15	102.76	101.24	104.85	99.05	100.94	100.09	102.94	98.75
232	Marbofloxacin	96.57	97.35	98.94	98.85	102.9	97.97	103.85	101.9	99.32	101.74
233	Maduramycin ammonium	99.31	101.64	99.49	100.54	95.31	102.38	100.7	97.03	99.92	98.21
234	Mapenterol	98.65	97.89	99.2	99.05	100.98	103.71	103.06	96.69	98.37	99.86
235	Mevastatin	101.68	97.84	101.85	102.92	104.5	97.1	102.86	95.48	97.65	100.14
236	Mesterolone	97.34	96.59	96.87	102.63	101.09	96.61	100.46	101.82	97.68	103.01
237	Melengestrol	97.45	101.71	98.57	98.46	100.84	103.27	102.05	102.44	96.25	102.18
238	Meloxicam	99.9	100.24	98.76	102.28	102.42	101.62	99.36	97.05	98.3	102.64
239	Medetomidine	97.63	96.05	102.22	98.9	100.65	95.3	102.91	100.88	96.12	98.45
240	Mestanolone	96.33	97.58	98.12	102.2	95.73	100.83	103.68	101.36	99.47	100.38
241	Methandrostenolone	96.44	101.53	99.72	101.06	96.47	103.52	100.09	102.3	103.85	99.57
242	Midazolam	97.73	102.25	96.44	101.73	99.87	95.21	100.36	98.03	101.37	98.35
243	Minocycline	96.83	98.24	99.47	101.85	102.54	100.8	100.68	98.62	101.93	102.58
244	Meprobamate	99.31	97.89	98.3	97.2	102.11	96.15	102.42	103.22	99.42	99.25
245	Muraglitazar	98.74	99.91	95.39	102.57	100	98.94	102.49	100.23	97.26	102.37
246	Mometasone	97.61	99.12	99.55	100.22	96.28	102.34	100.29	99.02	102.31	101.72
247	Moxidectin	101.29	97.02	97.15	101.99	96.75	103.83	103.88	97.49	98.6	99.27
248	Moxifloxacin	101.89	100.45	97.54	99.17	101.91	101.61	103.64	102.92	103.54	103.63
249	Nadifloxacin	101.87	101.22	98.9	99.79	97.72	99.54	101	97.87	97.54	101.09
250	Nabumetone	101.16	99.98	99.8	102.02	102.84	100.04	99.34	103.78	99.16	100.03
251	Nalidixic acid	101.59	99.34	99.14	99.46	100.56	95.98	102.28	101.2	102.27	100.11
252	Nafcillin	98.1	97	96.95	101.74	95.67	95.35	100.67	100	97.62	100.51
253	Naproxen	98.38	100.4	99.81	100.4	103.9	102.12	102.7	100.58	99.55	100.2
254	Nimodipine	102.38	99.36	98.9	103.06	97.17	99.47	100.04	96.33	96.19	99.86
255	Nitrendipine	99.3	97.5	99.96	102.96	102.05	99.21	99.77	102.09	100.75	101.88
256	Norfloxacin	95.45	98.85	97.86	100.73	98.99	96.73	100.97	100.58	99.99	102.91
257	Nortestosterone	98.78	98.98	99.96	99.04	101.23	102.72	99.54	100.45	98.49	99.18
258	Piperacillin	98.46	99.82	95.65	98.26	99.75	98.9	99.27	101.45	99.02	99.64
259	Prazosin	95.93	98.03	99.1	102.08	97.25	100.52	99.69	103.31	99.38	101.88
260	Pefloxacin	98.55	98.33	103.31	98.45	98.82	103.3	100.43	97.77	98.07	102.12
261	Penbutolol	100.96	97.93	98.57	102.59	104.59	103.44	99.66	103.88	98.8	98.58
262	Corticosterone	103.24	101.78	101.12	100.91	97.6	96.32	103.17	98.15	103.07	101.72
263	Prednicarbate	100.14	97.05	98.7	96.31	100.49	101.64	103.62	97.5	96.82	98.96

Number	Compounds	-18℃			+4℃			+18℃			-18℃
		1 month （%）	2 months （%）	6 months （%）	1 month （%）	2 months （%）	6 months （%）	1 month （%）	2 months （%）	6 months （%）	1 week （%）
264	Prednisone	97.75	99.33	98.07	95.8	96.43	101.22	103.77	97.91	98.61	98.43
265	Prednisolone	96.37	96.46	98.88	100.86	97.08	102.63	102.4	96.09	98.1	98.76
266	Propranolol	98.84	98.98	97.43	98.52	99.15	102.1	100.48	98.18	97.76	98.7
267	Hydroxy metronidazole	97.86	98.97	98.39	97.94	96.12	103.84	99.38	98.41	100.99	98.98
268	Hydroxy ipronidazole	98.05	98.83	97.75	96.11	101.73	100.52	98.9	96.88	100.07	103.69
269	Hydroxy dimetridazole	100.37	97.72	97.82	100.07	96.8	99.21	102.06	95.49	99.63	96.81
270	Oxymetholone	98.63	101.08	101.91	97.78	100.97	96.22	99.86	101.41	102.64	97.55
271	Penicillin G potassium	99.63	97.2	100.82	95.96	99.54	100.21	101.17	101.47	103.07	103.36
272	Penicillin V potassium salt	101.79	98.73	103.05	101.17	97.13	101.4	103.71	100.6	99.64	98.36
273	Hydrocortisone	99.37	102.54	99.92	101.28	103.87	103.69	99.05	97.44	98.65	97.57
274	Hydrocortisone 17-butyrate	100.89	96.4	98.28	98.86	102.37	101.8	101.96	101.23	99.78	101.64
275	Hydrocortisone 17-valerate	99.46	100.88	98.03	98.02	100.8	102.3	99.3	101.15	96.21	98.81
276	Fenoterol hydrobromide	98.86	97.44	98.28	97.16	96.72	98.42	101.48	100.94	98.84	102.21
277	Triamcinolone acetonide	100.36	102.7	101.93	98.74	103.55	100.61	100.38	95.04	98.3	102.18
278	Triamcinolone	102.54	99.12	103.94	97.74	100.22	101.35	102.6	100.62	99.29	98.09
279	Troglitazone	102.54	97.71	96.29	101.93	98.84	102.42	100.35	103.49	99.64	101
280	Desmethyl sibutramine	100.92	102.8	98.28	102.52	98.24	97.27	103.7	99.98	96.45	102.19
281	Dehydro lovastatin	97.31	101.64	98.28	99.98	97.68	103.41	99.21	99.77	100.88	98.02
282	Boldenone	97.98	97.57	97.1	97.19	97.17	103.04	101.51	101.91	97.01	103.44
283	Ethinyl estradiol	99.27	98.33	96.83	101.3	96.81	100.25	103.41	97.56	99.98	100.92
284	Quinestrol	99.66	102.75	101.67	100.75	100.27	100.36	99.99	99.21	97.62	96.26
285	Norethindrone	98.17	97.53	97.52	102.82	97.34	99.38	100.43	97.01	100.15	103.38
286	Trenbolone	102.57	99.09	98.1	100.39	101.09	102.34	101.01	102.4	102.7	97.74
287	Repaglinde	101.62	99.39	98.6	101.6	104.51	103.86	102.74	99.23	103.16	100.13
288	Secnidazole	98	98.66	101.34	102.03	102.96	97.3	103.6	97.2	100.07	98.36
289	Thiabendazole	97.18	99.85	98.19	99.14	100.77	103.71	101.21	95.7	98.89	102.04
290	Cyproheptadine	99.23	103.49	102.95	99.41	97.86	96.27	101.8	95.39	100.12	99.28
291	Diminazene	97.54	97.65	98.64	95.82	99.28	103.81	98.99	98.86	97.48	101.84
292	Ketotriclabendazole	99.32	99.36	102.91	95.03	100.15	100.48	101.96	99.89	98.93	99.86
293	Triclabendazole	99.83	97.76	96.93	97.97	104.54	100.97	100.79	96.64	102.26	103.58
294	Triclabendazole sulfone	95.99	99.43	100.5	101.52	96.03	98.18	100.06	99.51	98.15	101.33
295	Triazolam	96.98	97.26	96.66	100.41	97.13	99.63	99.16	103.12	97.55	98.65
296	Salbutamol	96.14	99.29	99.23	98.21	100.84	100.24	101.59	98.54	98.82	101.25
297	Sarafloxacin	98.4	100.52	100.77	99.56	98.54	96.63	99.54	97.67	99.62	98.66
298	Salmeterol	100.48	98.85	100.34	97.63	100.81	103.08	102.5	101.06	102.04	101.15
299	Sulindac	102.03	99.2	98.66	98.55	103.9	101.95	103.72	95.22	100.54	99.43
300	Difloxacin	101.14	96.18	99.41	101.34	97.02	103.63	99.25	100.59	99.32	103.85
301	Amitraz	102.68	97.25	101.72	99.34	100.59	98.84	102.01	95.71	102.38	103.2
302	Diclofenac acid	101.69	100.6	95.11	96.71	104.3	102.57	101.62	99.89	98.03	101.12

Number	Compounds	-18℃			+4℃			+18℃			-18℃
		1 month （%）	2 months （%）	6 months （%）	1 month （%）	2 months （%）	6 months （%）	1 month （%）	2 months （%）	6 months （%）	1 week （%）
303	Diclofenac sodium	102.88	95.58	96.64	102.95	103.06	95.34	99.65	101.06	101.37	101.03
304	Dihydrotestosterone	99.78	99.77	97.66	101.81	99.48	101.38	99.38	102.04	98.07	103.51
305	Sparfloxacin	99.41	98.1	99.31	99.64	103.11	98.69	100.63	98.51	103.28	97.05
306	Stanozolol	100.72	97.04	95.84	98.79	102.15	100.11	102.74	99.54	99.54	99.12
307	Tetracycline	98.44	99.61	100.02	97.77	102.96	102.48	102.46	95.83	99.12	99.13
308	Tylosin	99.5	100.52	103.66	100.31	100.19	101.95	101.35	102.48	100.87	99.22
309	Tiamulin	103.85	99.77	98.42	101.13	96.94	97.15	103.33	102.31	99.68	99.66
310	Terbutaline	100.89	99.47	98.56	97.89	104.84	97.17	100.22	100.89	102.67	100.14
311	Tilmicosin	102.99	98.28	97.49	102.15	97.17	97.98	99.56	98.94	102.18	98.41
312	Tenoxicam	100.74	101.71	95.6	97.63	104.25	97.59	103.49	103.62	99.03	102.95
313	Tinidazole	101.51	97.1	99.51	102.31	98.33	103.69	99	102.62	101.19	98.83
314	Tizanidine	101.48	101.9	98.29	100.68	102.81	103.07	102.32	99.65	100.28	99.01
315	Ketoprofen	100.76	98.36	99.4	98.77	103.47	97.55	102.42	99.4	101.21	98.14
316	Cephalexin	99.85	101.09	98.31	100.32	98.22	103.5	102.82	97.06	98.49	101.67
317	Cefotaxime	102.75	99.71	98.91	99.76	99.86	103.12	101.19	100.79	96.23	98.72
318	Cefaclor	96.31	99.44	99.95	100.9	101.33	96.06	99.1	99.72	103.13	99.3
319	Cefixime	96.19	98.38	98.24	102.28	101.68	100.53	101.87	103.33	103.38	103.59
320	Cefquinome	97.09	96.33	99.54	98.92	103.38	97.02	100.45	96.54	101.79	97.48
321	Cephradine	95.7	96.66	96.26	100.64	102.54	98.61	100.32	96.22	103.49	98.51
322	Cephalonium	98.6	97.16	102.93	100.44	97.5	99.6	103.53	98.29	97.76	101.34
323	Cefamandole	97.25	100.06	98.64	98.64	98.58	99.76	101.89	100.92	97.79	102.27
324	Cefminox	99.85	103.53	102.95	102.58	99.46	98.31	99.36	102.62	100.66	96.81
325	Cefoperazone	95.99	97.58	101.03	100.27	99.62	97.39	101.67	99.89	103.94	99.18
326	Cephapirin	98.91	102.8	97.5	99.17	98.29	96.74	102.53	98.95	102.57	102.06
327	Cefpirome	97.92	95.72	97.96	102.76	104.77	98.87	99.7	101.72	98.79	98.16
328	Cefadroxil	99.71	102.02	99.31	101.56	98.94	96.74	101.07	100.53	103.25	103.22
329	Ceftiofur	96.91	99.3	99.46	100.22	104.49	99.82	100.66	99.46	100.57	100.17
330	Ceftazidime	96.36	97.51	98.23	98.88	103.4	103.89	101.87	97.76	101.27	98.27
331	Cefetamet pivoxil	98.53	102.41	100.31	96.3	104.54	95.31	103.33	99.35	102.05	102.11
332	Cefazolin	99.35	102.56	98.19	99.69	100.73	102.43	103.12	98.38	99.72	102.09
333	Oxytetracycline	99.05	100.28	99.79	100.98	97.91	102.2	100.55	97.39	103.93	99.91
334	Melatonine	98.58	97.22	96.41	102.34	104.07	99.28	101.16	95.83	102.96	99.43
335	Tolmetin	97.22	99.63	99.08	99.37	98.94	100.61	99.44	97.36	97.12	101.82
336	Tolfenamic acid	99.77	95.79	98.52	98.45	102.63	99.82	101.73	98.64	99.07	101.36
337	Dehydroepiandrosterone	101.2	96.65	96.03	101.09	98.58	97.53	102.79	95.84	102.85	97.03
338	Anhydroerythromycin A	101.06	101.84	103.73	97.25	98.63	99.66	100.57	100.68	103.06	99.44
339	Desoxycarbadox	103.64	102.78	101.21	101.35	99.08	99.6	99.02	103.14	99.98	98.6
340	Cortexolone	102.09	97.15	97.93	100.24	95.72	96.14	100.23	95.43	98.37	100.84
341	Tulobuterol	100.69	97	98.24	96.59	98.32	99.92	103.38	100.76	103.68	99.99

Number	Compounds	-18℃			+4℃			+18℃			-18℃
		1 month （%）	2 months （%）	6 months （%）	1 month （%）	2 months （%）	6 months （%）	1 month （%）	2 months （%）	6 months （%）	1 week （%）
342	Toltrazuril	97.37	96.6	96	102.63	99	99.37	100.14	97.01	102.41	100.24
343	Toltrazuril sulfoxide	102.77	96.97	100.69	100.51	96.84	101.22	100.04	98.25	97.82	98.64
344	Vildagliptin	99.4	98.54	101.57	100.8	104.93	98.89	102.67	102.23	96.94	102.97
345	Virginiamycin M1	98.01	102.88	101.5	102.26	99.12	99.82	103.14	100.44	103.96	97.44
346	Venlafaxine	98.77	100.21	97.69	101.4	95.77	100.77	100.32	96.1	99.84	99.87
347	Sildenafil	96.65	99.91	102.72	98.82	103.55	103.14	101.14	99.38	97.9	103.58
348	Cimaterol	97.04	95.63	100.26	101.64	102.32	97.39	101.21	102.58	98.6	99.47
349	Cinoxacin	96.14	99.31	99.51	100.75	101.42	97.1	100.15	98.14	97.42	99.24
350	Sitagliptin	97.06	98.23	98.46	100.34	104.2	95.14	102.93	97.96	99.76	97.12
351	Nifedipine	97.86	100.77	104.85	97.13	98.36	100.86	103.38	96.89	100.39	98.25
352	Nitrazepam	102.18	101.66	98.86	99.35	97.68	95.35	100.84	103.82	96.52	102.63
353	Simvastatin	103.3	100.84	95.28	99.38	98.67	97.15	101.24	99.57	101.79	100.72
354	Androsterone	101.59	103.33	101.38	97.19	97.57	97.55	103.33	97.17	98.17	100.12
355	Bromchlorbuterol	100.26	101.78	101.38	102.01	104.16	100.65	102.94	101.36	103.15	101.83
356	Ofloxacin	101.82	102.05	100.37	98.26	99.27	98.32	103.76	102.4	100.45	100.18
357	Enoxacin	98.58	99.41	98.19	102.94	102.75	103.23	102.51	96.05	103	102.75
358	Eprinomectin	101.08	98.42	102.02	101.1	102.3	98.33	103.71	98.78	98.45	103.36
359	Megestrol acetate	99.99	97.48	98.77	98	103.73	99.53	101.21	96.68	98.49	102.19
360	Mequindox	102.2	95.91	100.38	99.23	95.2	96.78	99.26	100.6	97.73	97
361	Ipronidazole	98.33	99.3	99.24	97.5	95.59	100.15	103.01	99.83	98.58	103.5
362	Isochlortetracycline	98.36	98.58	98	97.94	104.87	99.46	103.76	97.84	97.46	101.29
363	Indomethacin	102.57	101.14	100.58	99.62	96.34	98.12	99.88	101.32	98.07	99.03
364	Leucomalachite green	98.8	100.02	98.25	101.55	104.03	102.97	99.97	103.42	96.27	102.52
365	Leucocrystal violet	97.19	104.22	98.83	98.91	101.3	101.93	101.03	95.83	103.93	98.62
366	Indoprofen	97.04	99.21	96.14	99.54	102.34	98.42	102.48	96.53	102.91	100.15
367	Progesterone	102.95	104.55	98.31	100.58	101.11	96.11	102.65	96.22	102.3	100.35
368	Pregnenolone	99.9	98.1	103.17	102.56	98.86	103.33	101.43	97.88	98.51	100.65
369	Zaleplon	97.63	98.89	97.88	102.54	98.67	100.92	103.29	95.82	102.51	99.47
370	Oleandomycin	99.87	97.2	102.66	97.39	100.59	98.03	102.05	100.02	103.88	99.55
371	Levamisole	101.73	102.51	97.41	97.84	98.33	101.3	100.33	97.21	98.99	99.3
372	Levofloxacine	102.65	96.66	104.38	97.4	104.74	102.85	99.23	96.14	99.99	97.02
373	Zolpidem	97.77	96.16	103.95	97.71	104.91	99.16	103.89	98.85	100.6	101.96
374	3,5-Dinitrobenzamide	95.46	103.64	95.71	100.58	99.09	101.79	101.1	99.91	99.62	99.37
375	ez-Diethylstilbestrol	102.91	100.19	99.98	102.99	96.55	103.43	99.07	103.13	98.08	97.47
376	Analgin	98.67	97.87	100.86	102.47	103.61	102.16	103.12	97.56	99.11	102.19
377	Barbital	99.9	101.86	102.52	100.2	99.66	100.58	102.34	102.69	101.89	99.4
378	Phenobarbital	99.45	98.67	98.57	95.1	99	95.99	100.83	102.4	103.52	102.79
379	Ibuprofen	98.99	101.14	99.75	97.22	99.47	97.97	100.65	99.59	99.73	97.06
380	Dapagliflozin	98.64	100.29	96.31	100.16	96.11	102.36	103.87	101.25	97.37	100.94

Number	Compounds	-18℃			+4℃			+18℃			-18℃
		1 month （%）	2 months （%）	6 months （%）	1 month （%）	2 months （%）	6 months （%）	1 month （%）	2 months （%）	6 months （%）	1 week （%）
381	Diclazuril	101.07	99.89	100.72	100.76	98.64	100.03	99.42	100.98	103.58	98.48
382	Diflunisal	98.72	99.13	98.7	98.57	97.21	97.14	102.43	101.76	101.65	102.25
383	Sodium nifurstylenate	99.62	98.24	99.17	99.94	101.52	102.79	101.99	97.81	98.91	100.93
384	Furosemide	98.29	99.39	99.79	99.79	101.17	103.05	101.03	97.33	98.02	102.64
385	Florfenicol	98.92	99.11	97.22	101.68	101.15	99.39	103.04	102.91	100.37	97.18
386	Flurbiprofen	99.55	100.95	99.37	101.16	98.58	101.86	101.57	103.94	101.95	99.74
387	Ciglitazone	98.06	99.67	97.72	99.51	103.28	100.9	103.05	98.88	101.72	97.63
388	Hexestrol	97.69	100.32	98.25	97.6	101.3	96.57	99.89	95.98	96.45	100.81
389	Diethylstilbestrol	98.66	97.32	99.79	97.6	103.19	102.54	101.54	96.29	96.66	96.81
390	Narasin	101.98	97.81	99.43	99.37	98.93	103.67	102.81	99.17	101.82	96.98
391	Lasalocid	97.82	98.11	96.05	98.3	103.96	99.24	101.61	100.51	97.01	102.56
392	Monensin	97.63	103.47	102.53	100.31	101.25	103.33	102.83	97.53	103.87	99.42
393	Nicarbazin	99.63	97.35	98.92	101.3	103.85	101.86	99.81	102.23	97.25	99.04
394	Hydrochlorothiazide	99.67	99.44	99.65	99.64	102.2	98.02	103.67	99.23	103.05	102.67
395	Sulbactam	97.93	101.43	99.3	99.16	102.73	97.43	103.51	96.83	103.72	99.47
396	Dienestrol	98.22	99.93	99.9	101.88	97.92	99.47	99.75	97.79	96.11	99.87
397	Secobarbital	97.2	99.56	98.92	98.75	101.52	100.1	100.32	99.24	101.58	97.02
398	Ponazuril	100.04	102.21	101.15	99.54	98.9	98.29	100.81	101.44	100.74	102.16
399	Nitroxynil	101.68	99.43	99.76	99.44	99.54	99.54	99.31	95.81	101.35	101.98
400	Salinomycin	100.35	97.78	97.12	100.03	99.05	103.41	102.7	102.88	97.02	98.96
401	Amobarbital	97.06	102.16	102.23	102.88	103.06	101.72	99.46	102.68	102.66	99.97
402	4,4′-Dinitrocarbanilide	99.67	101.73	99.24	101.4	97	99.71	98.77	102.25	100.93	97.15
403	Propetamphos	99.52	100.82	96.88	97.91	101.43	97	103.13	99.92	99.96	101.06
404	Fenthion	97.25	96.18	99.75	103.79	102.92	100.69	101.89	98.01	98.29	101.38
405	Ricinine	97.03	102.37	95.22	101.87	98.54	100.8	102.17	98.93	103.56	98.86
406	Trichlorfon	97.28	97.15	100.12	98.36	104.47	98.85	101.26	102.47	98.76	100.5
407	Dichlorvos	101.88	101.65	96.17	99.92	96.22	101.55	101.85	102.57	103.61	97.95
408	Fluazuron	97.97	100.47	97.45	96.63	97.15	102.25	100.78	95.34	101.76	101.13
409	Diazinon	101.89	97.42	98.56	98.11	102.05	98.55	100.63	101.84	99.32	100.33
410	Malathion	99.17	99.7	99.77	102.61	99.79	102	99.03	95.34	96.44	96.92
411	Phoxim	101.7	103.45	98.46	99.49	96.07	98.1	101.34	101.46	99.02	100.73
412	Ethopabate	99.57	102.07	101.17	99.35	98.11	98.33	102.2	101.27	102.18	100.83
413	Salicylic acid	101.69	99.29	96.78	102.08	97.27	96.91	102.24	102.12	97.87	99.25
414	Sodium pentachlorophenoxide	97.22	102.39	102.53	98.97	99.07	98.26	103.89	95.96	95.28	101.6
415	α-Zearalenol	101.53	103.02	95.88	99.75	100.12	99.13	99.72	100.22	103.36	99.58
416	β-Zearalanol	98.99	99.74	95.95	102.14	98.89	98.42	100.09	101.53	99.32	100.52
417	β-Zearalenol	98.53	98.09	95.53	98.57	100.24	102.49	101.12	96.56	97.15	102.56
418	Zearalanol	96.59	98.07	101.74	102.81	102.94	101.9	101.75	96.88	96.66	97.02

Number	Compounds	-18°C			+4°C			+18°C			-18°C
		1 month （%）	2 months （%）	6 months （%）	1 month （%）	2 months （%）	6 months （%）	1 month （%）	2 months （%）	6 months （%）	1 week （%）
419	Zearalanone	97.37	101.13	104.19	99.43	97.03	96.51	101.81	99.68	96.8	96.37
420	Zearalenone	101.59	98.52	99.1	100.38	100.83	100.08	100.87	97.35	98.41	99

Table S6. Information on samples containing risk substances

Number	Sample type	Drug
1	Feed materials	Dipterex, levomisole
2	Feed materials	Albendazole, Albendazole sulfoxide, Acetaminophen, Dioxopromethazine, Diclofenac acid
3	Feed materials	Florfenicol
4	Mixed feed additives	Albendazole, Tylosin, Tilmicosin
5	Mixed feed additives	Mequindox
6	Mixed feed additives	Flunixin
7	Feed materials	Albendazole, Oxfendazole, Fenbendazole, Florfenicol
8	Feed materials	Albendazole, Caffeine
9	Feed materials	Albendazole, Acetaminophen, Tylosin, Tilmicosin
10	Feed materials	Indomethacin
11	Feed materials	Mequindox
12	vitamin premix feed	Phenacetin
13	Mixed feed additives	Caffeine
14	Feed materials	Pseudoephedrine
15	Mixed feed additives	Indomethacin
16	Feed materials	Mequindoxl, Oxytetracycline, Tetracycline
17	Mixed feed additives	Enrofloxacin
18	Composite premix feed	Albendazole
19	vitamin premix feed	Albendazole
20	Feed materials	Albendazole, Mequindox, 3-Methyl-quinoxaline-2-carboxylic acid
21	Feed materials	Atropine
22	Mixed feed additives	Enrofloxacin, Sinomenine, Mequindox
23	Composite premix feed	Florfenicol
24	Composite premix feed	Phenacetin
25	Composite premix feed	Mequindox
26	Mixed feed additives	Atropine, Mequindox
27	vitamin premix feed	Phenacetin

28	Composite premix feed	Albendazole, Tilmicosin
29	Mixed feed additives	Chlorpheniramine maleate
30	Mixed feed additives	Chlorpheniramine maleate
31	Mixed feed additives	Ciprofloxacin, Mequindox, Chlorpheniramine maleate
32	Mixed feed additives	Ciprofloxacin, Chlorpheniramine maleate
33	Mixed feed additives	Mequindox
34	Mixed feed additives	Albendazole
35	Mixed feed additives	Albendazole, Mequindox
36	Feed materials	Ephedrine
37	Mixed feed additives	Trimethoprim, Mequindox
38	Feed materials	Olaquindox, Acetaminophen
39	Feed materials	Olaquindox, Enrofloxacin, Mequindox
40	Feed materials	Trimethoprim, Mequindox, Florfenicol
41	Feed materials	Mequindox
42	Mixed feed additives	Enrofloxacin, Mequindox
43	Feed materials	Albendazole, Albendazole sulfoxide, Fenbendazole, Mequindox
44	Mixed feed additives	Oxazepam
45	Mixed feed additives	Tilmicosin, Acetaminophen, Tylosin
46	Mixed feed additives	Florfenicol
47	Feed additives	Caffeine
48	Feed materials	Albendazole
49	Mixed feed additives	Caffeine, Acetaminophen, 4-Methylamino antipyrine
50	Mixed feed additives	Albendazole
51	Composite premix feed	Caffeine
52	Mixed feed additives	4,4′-Dinitrocarbanilide, Nicarbazin

Supplementary Material

a

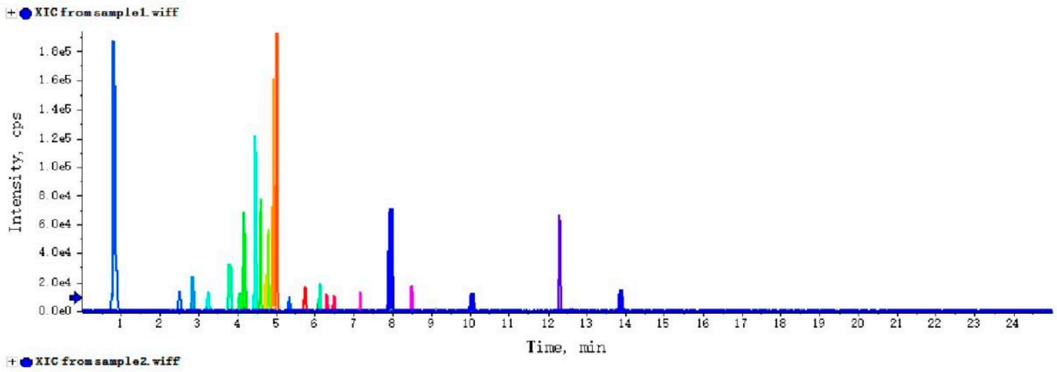
XIC from sample1.wiff
Intensity, cps
Time, min

b

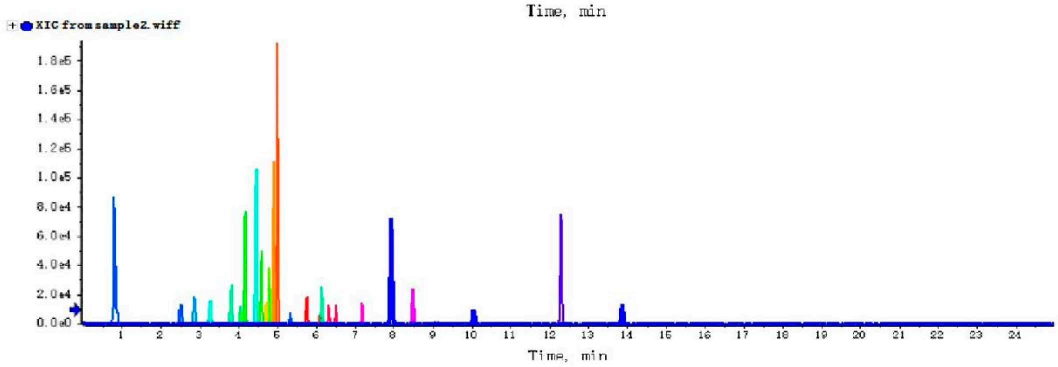
XIC from sample2.wiff
Time, min

c

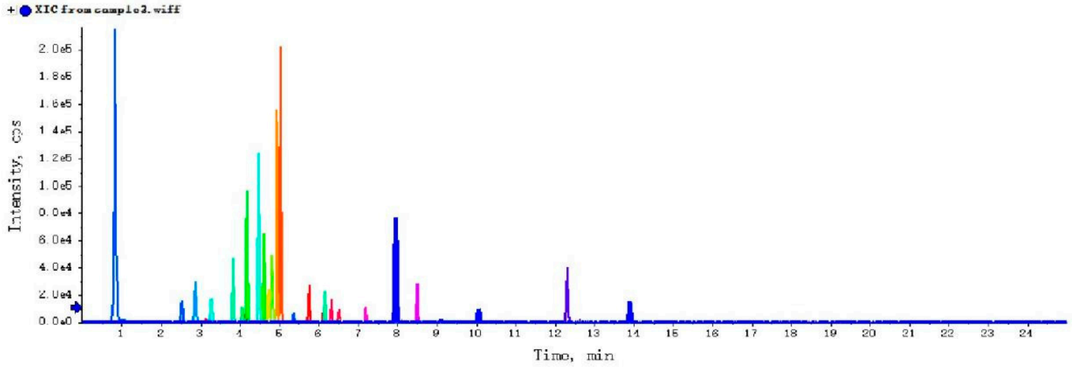
XIC from sample3.wiff
Intensity, cps
Timo, min

d

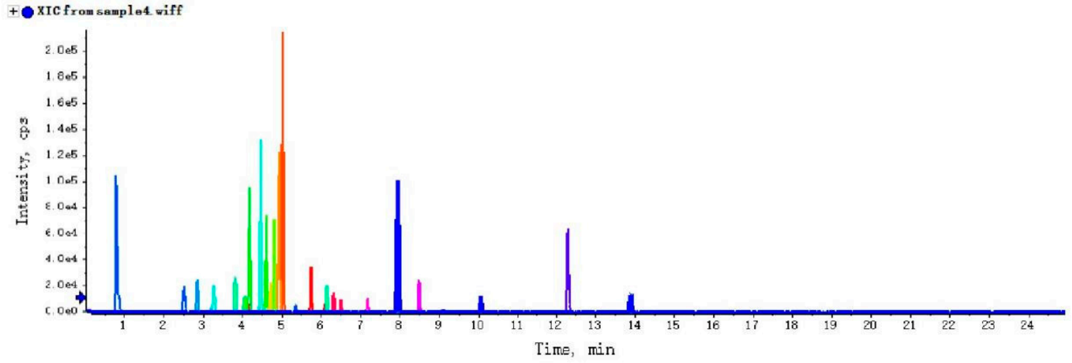
XIC from sample4.wiff
Intensity, cps
Time, min

e

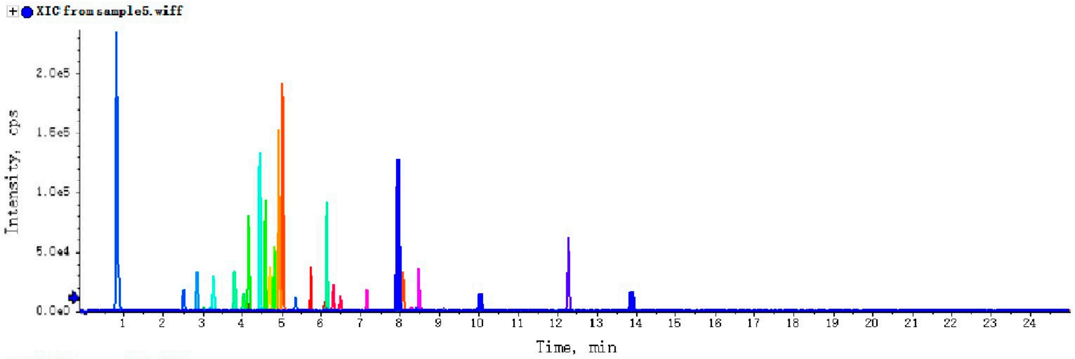
XIC from sample5.wiff
Intensity, cps
2.0e5
1.5e5
1.0e5
5.0e4
0.0e0
1 2 3 4 5 6 7 8 9 10 11 12 13 14 15 16 17 18 19 20 21 22 23 24
Time, min

f

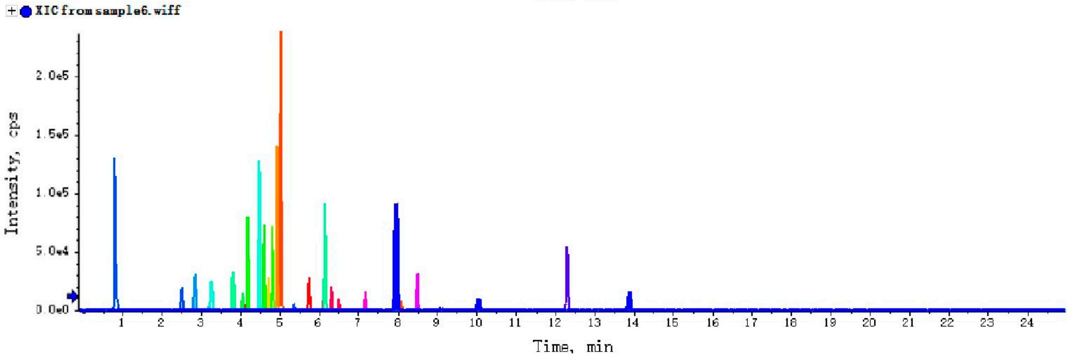
XIC from sample6.wiff
Intensity, cps
2.0e5
1.5e5
1.0e5
5.0e4
0.0e0
1 2 3 4 5 6 7 8 9 10 11 12 13 14 15 16 17 18 19 20 21 22 23 24
Time, min

g

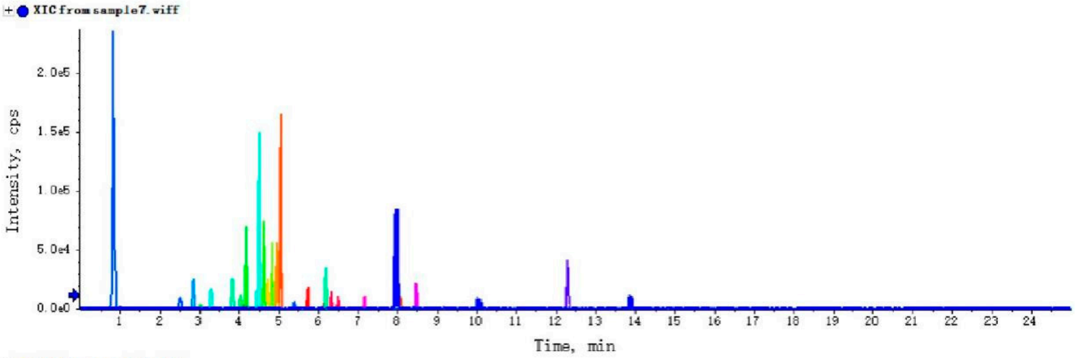
XIC from sample7.wiff
Intensity, cps
2.0e5
1.5e5
1.0e5
5.0e4
0.0e0
1 2 3 4 5 6 7 8 9 10 11 12 13 14 15 16 17 18 19 20 21 22 23 24
Time, min

h

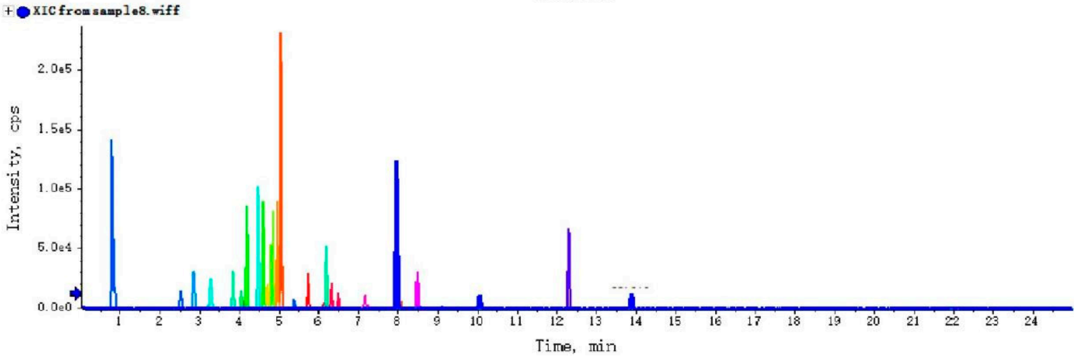
XIC from sample8.wiff
Intensity, cps
2.0e5
1.5e5
1.0e5
5.0e4
0.0e0
1 2 3 4 5 6 7 8 9 10 11 12 13 14 15 16 17 18 19 20 21 22 23 24
Time, min

i

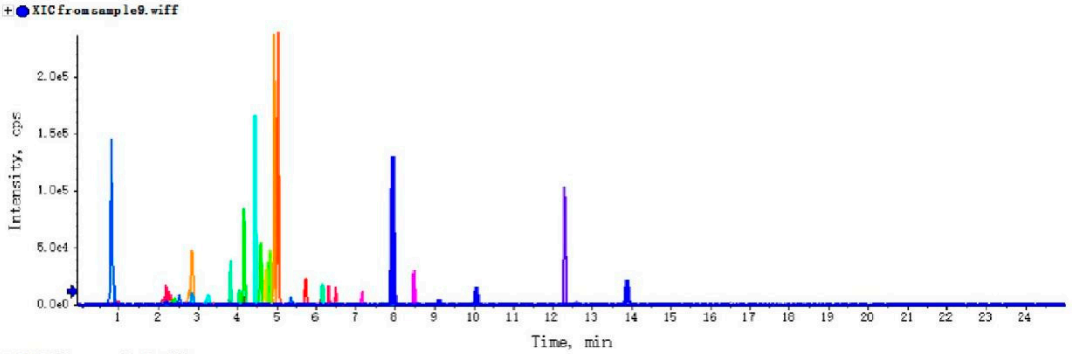
XIC from sample9.wiff
Intensity, cps
Time, min

j

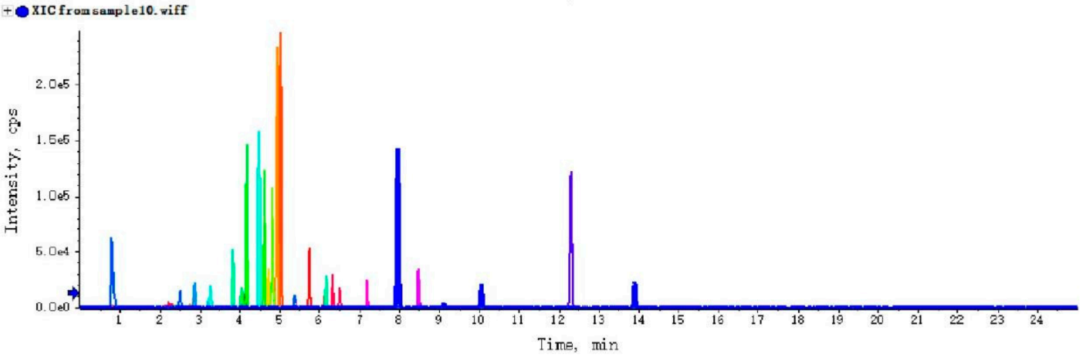
XIC from sample10.wiff
Intensity, cps
Time, min

k

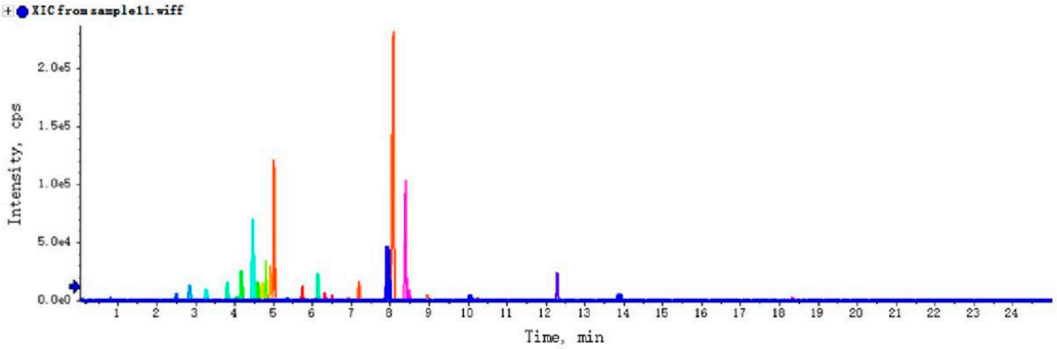
XIC from sample11.wiff
Intensity, cps
Time, min

l

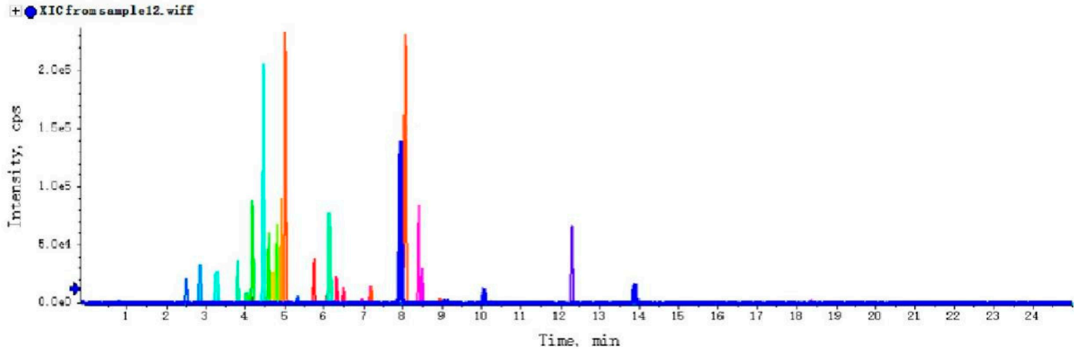
XIC from sample12.wiff
Intensity, cps
Time, min

m

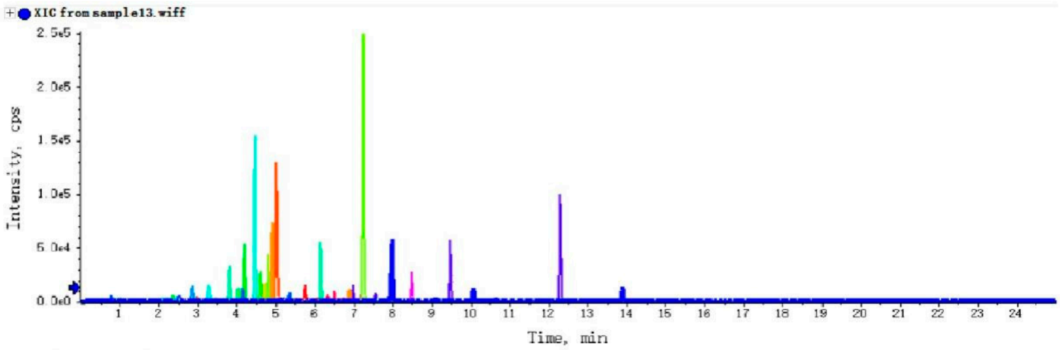
XIC from sample13.wiff
Intensity, cps
Time, min

n

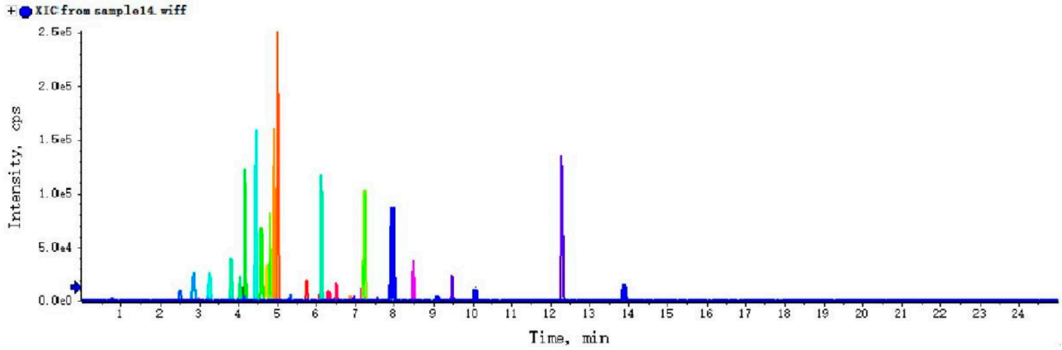
XIC from sample14.wiff
Intensity, cps
Time, min

o

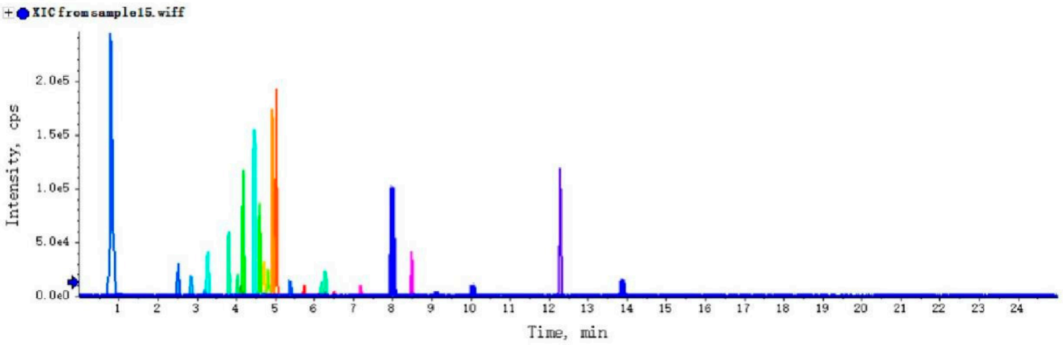
XIC from sample15.wiff
Intensity, cps
Time, min

p

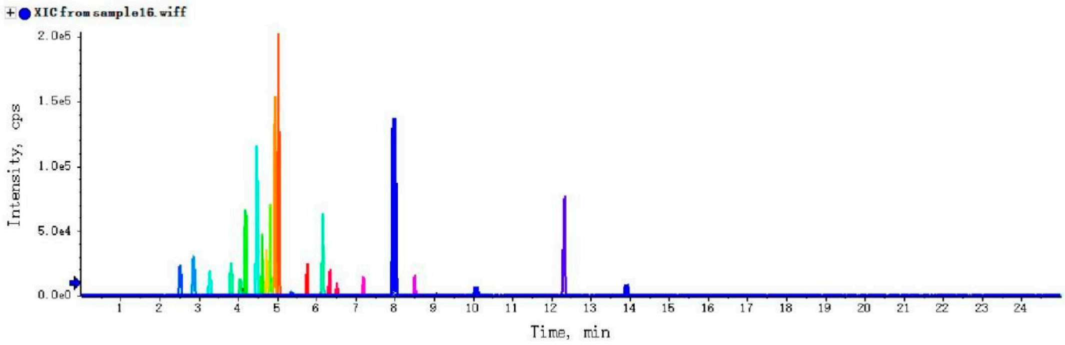
XIC from sample16.wiff
Intensity, cps
Time, min

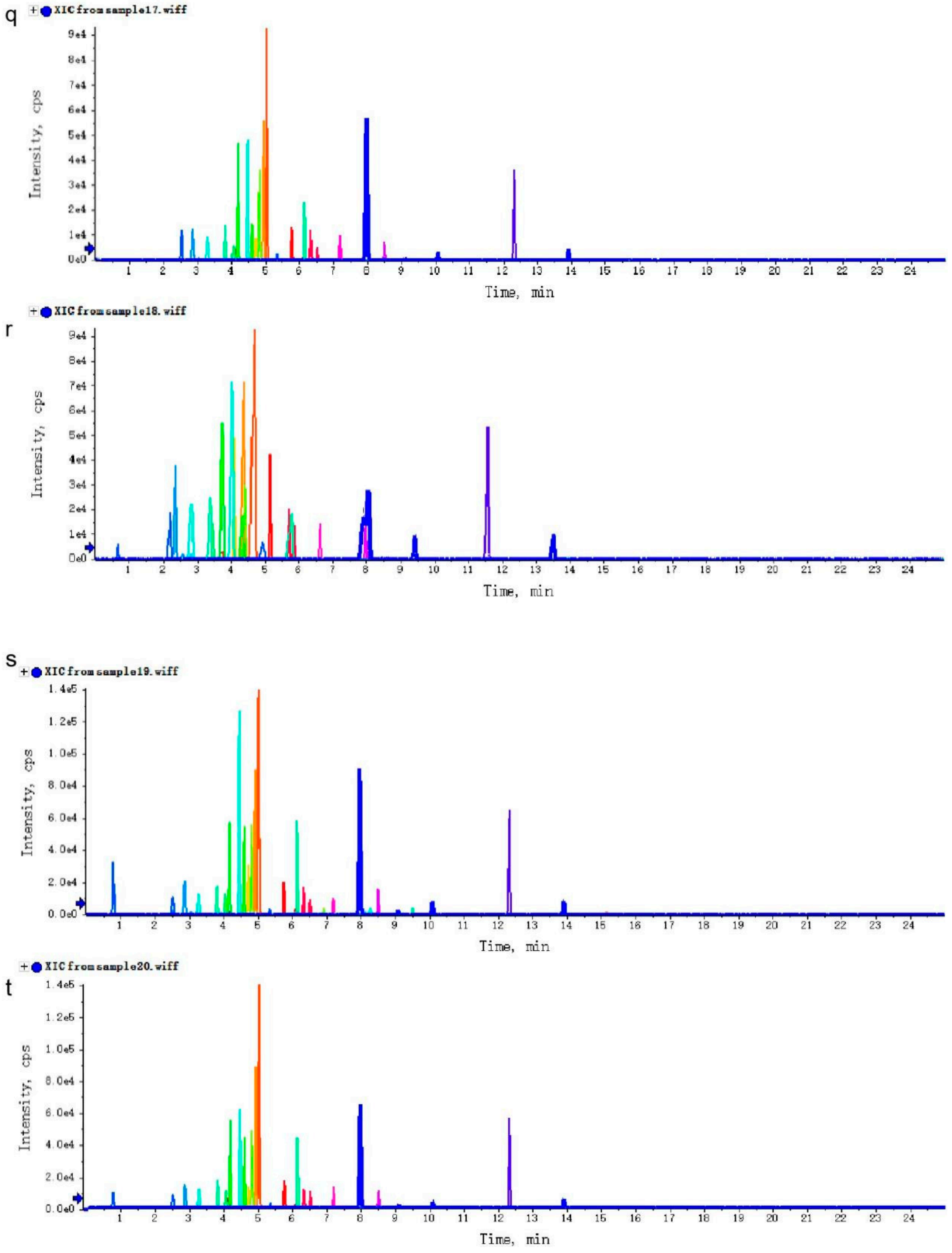

Figure S1. The XIC of 20 compounds in different matrices. (a,b):Pig formula feed; (c,d):Chicken formula feed; (e,f):Pig concentrated feed; (g,h):Chicken concentrated feed; (i,j):Cow concentrate supplement (k,l):Composite additive premix; (m,n):Food flavour; (o,p):Plant extracts (Radix Astragali); (q,r):Trace mineral feed; (s,t):Vitamin premix feed.
